# Changes in the pulmonary surfactant in patients with mild to moderate COVID-19

**DOI:** 10.1371/journal.pone.0325153

**Published:** 2025-08-07

**Authors:** Spela Kokelj, Per Larsson, Emilia Viklund, Hatice Koca, Hanna Slogén, Lowie Vanfleteren, Bo Nilsson, Karin Fromell, Johan Westin, Anna-Carin Olin

**Affiliations:** 1 Occupational and Environmental Medicine, School of Public Health and Community Medicine, Institute of Medicine, Sahlgrenska Academy, University of Gothenburg, Gothenburg, Sweden; 2 COPD Center, Department of Respiratory Medicine and Allergology, Sahlgrenska University Hospital, Gothenburg, Sweden; 3 Department of Internal Medicine and Clinical Nutrition, Institute of Medicine, Sahlgrenska Academy, University of Gothenburg, Gothenburg, Sweden; 4 Department of Immunology, Genetics and Pathology, Uppsala University, Uppsala, Sweden; 5 Department of Infectious Diseases, Institute of Biomedicine, University of Gothenburg, Gothenburg, Sweden; 6 Department of Infectious Diseases, Sahlgrenska University Hospital, Gothenburg, Sweden; Tribhuvan University, NEPAL

## Abstract

**Introduction:**

Changes in the pulmonary surfactant have been seen in severe COVID-19, but data on mild to moderate COVID-19 is scarce. The aim of this study was to explore the protein and phospholipid profiles in the small airways in patients with mild to moderate COVID-19.

**Methods:**

29 cases with COVID-19 and 17 healthy controls were examined at baseline. 22 cases were re-examined at follow-up after recovery from COVID-19. Airwave oscillometry was performed and the biological material from the respiratory tract lining fluid was collected with the PExA (Particles in Exhaled Air) method. SOMAscan was used for the analysis of proteins, and liquid chromatography with tandem mass spectrometry (LC-MS/MS) for phospholipids.

**Results:**

95 lipid species belonging to 8 lipid classes, and 46 proteins were analysed. Relative amounts of 13 lipid species differed between cases and controls at baseline, and of 24 lipid species at follow-up. At follow-up, the phosphatidylethanolamine class (PE) was significantly lower in cases at than in controls, and a significant decrease in PE, as well as a change in 20 lipid species from baseline to follow-up in cases was seen. The protein profile did not differ between cases and controls either at baseline or follow-up, or between repeated measurements in cases.

**Conclusions:**

The observed alterations in the surfactant phospholipids in the RTLF indicate that surfactant homeostasis is affected already in mild to moderate COVID-19, and these changes appear to persist over time.

## Introduction

COVID-19 is a disease caused by severe acute respiratory syndrome coronavirus 2 (SARS-CoV-2), with a wide spectrum of clinical presentations, from asymptomatic infection, to critical illness characterized by acute respiratory distress syndrome (ARDS) and multi-organ failure [1]. Following an acute infection, generally defined by the presence of symptoms and lasting up to four weeks [2], some individuals report persistent symptoms, including dyspnoea, persistent cough, cognitive impairment, fatigue and decreased quality of life, called post-acute sequalae of COVID-19 (PASC), or “long COVID”. These symptoms can be new, recurring or ongoing and are present more than four weeks after the onset of infection [3]. It has been previously shown that dyspnoea after COVID-19 is associated with small airway dysfunction, which can be present even a year after a mild infection [4].

SARS-CoV-2 enters the host cells through the angiotensin-converting enzyme 2 (ACE2) receptor, which is highly expressed on alveolar type II (AT2) cells in the distal lung. This causes severe damage to the pulmonary surfactant-producing AT2 cells, which in turn leads to the disruption of the surfactant homeostasis [5]. The pulmonary surfactant consists mainly of phospholipids (90% of weight) and proteins (10% of weight). Its major role is to stabilize the alveoli at the end of each respiratory cycle by reducing surface tension. It also plays an important role in the host defence against inhaled materials and pathogens. Dysfunction of the pulmonary surfactant is associated with numerous lung diseases, such as ARDS, interstitial lung disease, and pneumonia [6].

It has been previously reported that phosphatidylglycerols (PG) and phosphatidylinositols (PI), minor classes of surfactant phospholipids, are very potent inhibitors of the innate immune system in the lung, which act by blocking the activation of multiple Toll-like receptors [7]. These lipids have been also shown to have anti-viral effects against a broad range of respiratory RNA viruses, including SARS-CoV-2 [8], while some phospholipids render the airway epithelium more susceptible to viral entry [6]. Changes in the phospholipid profile in bronchoalveolar lavage fluid (BALF) have been observed in patients with inflammatory lung injury (ARDS and severe pneumonia), with a decrease in phosphatidylcholine (PC) and PG and increase in PI, sphingomyelin (SM), phosphatidylethanolamine (PE) and phosphatidylserine (PS) [6,9]. Furthermore, decreased dipalmitoylphosphatidylcholine (DPPC; PC 16:0/16:0)) levels in BALF of COVID-19 patients with moderate-to-severe ARDS have also been observed [10].

Although the surfactant comprises the major part of the respiratory tract lining fluid (RTLF), proteins originating from various other sources can also be found in the RTLF, e.g., proteins originating from the respiratory epithelial cells and inflammatory cells, and plasma proteins [11,12]. Previous proteomic analyses of exhaled particles collected non-invasively from hospitalized patients with COVID-19 have revealed alterations in the proteins involved in the stress response, acute phase response, as well as a decrease in Pulmonary surfactant associated protein B [11]. Furthermore, systemic activations of the complement and kallikrein-kinin systems are some of the key contributors to the COVID-19 pathogenesis and the thromboinflammation associated with it [13,14]. We have previously observed a high abundance of complement proteins in the RTLF collected with the exhaled particle method [15] and studies on BALF have reported SARS-CoV-2 induced dysregulation of the kallikrein-kinin system [16].

Increased complement activation in blood has also been observed in long COVID. Patients with long COVID already exhibited increased complement activation during the acute infection and these changes persisted at six-month follow-up [17]. Furthermore, proteomic and immunological abnormalities in the airways have been reported in these patients, with an elevated concentration of proteins associated with apoptosis, tissue repair and epithelial injury. Additionally, these changes appear to be most prominent in the airways rather than peripheral blood, highlighting the importance of studying biological changes locally in the lungs [18].

In the current study, we tested the hypothesis that SARS-CoV-2 infection causes alterations in the lipid and protein profiles in the small airway respiratory tract lining fluid in mild to moderate disease. Furthermore, we investigated whether the alterations in the lipid and protein profiles change over time, as well as whether they are associated with small airway dysfunction in COVID-19.

## Methods

### Study design and participants

This is an observational prospective cohort study, including 29 subjects with confirmed SARS-CoV-2 infection and 17 healthy controls. The study was carried out at Sahlgrenska University Hospital, Gothenburg, Sweden and the participants were recruited between the 17 February 2021 and 14 October 2022. The Regional Ethics Committee at the University of Gothenburg approved the study (2020–07184) and participants provided written informed consent prior to the measurements.

Individuals with COVID-19 (cases) were recruited through two different channels. Subjects who tested positive for SARS-CoV-2 infection at primary health centres in the Gothenburg area have been invited to participate in the study as well as hospital health care workers who tested positive for COVID-19 during routine testing for hospital staff. Mild to moderate illness was defined as a positive PCR of oro/nasopharyngeal sample and the presence of mild symptoms (e.g., cough, fever, change in taste or smell) with or without dyspnoea, and without the need for hospitalization, according to Gandhi et al. [19]. Exclusion criteria were current smoking, defined as smoking daily during the last twelve months, and the need for hospital treatment and oxygen supplementation. Healthy controls were recruited by posted notices among hospital health care workers who have never had a confirmed COVID-19, were without current symptoms of COVID-19 and had a negative rapid SARS-CoV-2 antigen test (Boson Biotech, Xiamen Boson Biotech Co., Ltd, Xiamen, China) at the time of examination. Exclusion criteria for healthy controls were current smoking and the presence of any respiratory symptoms.

The cases were examined during the acute stage of the disease at baseline (8 days of median time since symptom onset (IQR 5–12 days)). They were also invited for a follow-up examination (192 days of median follow-up (IQR 111–216 days)) after the first visit where the same protocol was carried out and information about disease progression was obtained. Seven subjects dropped-out of the study at the time of follow-up and consequently, 22 cases were re-examined at follow-up (S1 Fig). Healthy controls were examined only on one occasion at baseline.

The study protocol consisted of a detailed questionnaire about symptoms and medical history, airwave oscillometry and sampling of exhaled particles and blood.

### Airwave oscillometry (AOS)

Lung function was measured with AOS using Tremoflo C-100 (Thorasys, Montreal, Canada) according to the ERS technical standards [20]. Resistance at 5 Hz (R_5_), frequency dependence of resistance (R_5-20_) and reactance area (AX) were assessed and the results were expressed as z-scores using international reference values [21].

### Exhaled particles

Exhaled particles (PEx) were collected using the PExA instrument (PExA AB, Gothenburg, Sweden), as previously described [15,22]. In short, study participants wore a nose clip and breathed into the PExA instrument via a mouthpiece and a two-way, non-re-breathing valve, inhaling HEPA-filtered air to remove particles from ambient air. Exhaled particles in diameters between 0.4 and 7 µm were counted by an optical particle counter (Grimm Aerosol Technik GmbH & Co, Ainring, Germany) in the instrument and sampled on the impactor (Dekati Ltd, Tampere, Finland). A standardized breathing manoeuvre was used [23], starting with an exhalation at normal flow rate to residual volume, breath holding for 5 seconds, followed by a maximal inhalation to total lung capacity, immediately followed by a normal exhalation to functional residual capacity. Between breathing manoeuvres, the study participants took 3–5 tidal breaths into the instrument. Each sampling session continued until 120 ng of exhaled particles were collected or until the subject no longer wished or was unable to continue with the sampling. After collection, the hydrophilic polytetrafluorethylene (PTFE) membranes (FHLC02500, Millipore, Billerica, MA, USA) on the impactor plate were cut in half and placed in separate, 1.5 ml SC microtube PC-PT cryotubes (Sarsteds, Nümbrecht, Germany) and stored at −80°C for subsequent extraction and analysis.

### Lipid analysis and processing of data

A detailed list of all lipid species analysed in the study is provided in S1 Table. Internal standard (SPLASH ® LIPIDOMIX 330707−1EA, Avanti Polar lipids, Alabaster, AL, USA) was diluted 200x in methanol and 5 µl was spiked to each sampling membrane and allowed to dry for 10 minutes. Extraction was made in the cryotubes using 400 µl of isopropanol and a thermomixer (40°C and 700 RPM). The eluates were transferred to LC vials using a CTC-PAL robot. A second extraction was made with the same procedure using methanol that is a more polar solvent. Eluates were combined in the LC-vials (total of 800 µl solvent) and evaporated to dryness under a flow of nitrogen gas. The remaining lipid film was solvated in 90 µl of acetonitrile and isopropanol (mixed ratio 2:1) that was also the injection solvent for the analysis. The extracted lipids were analysed with a targeted LC-MSMS method using the Waters ACQUITY UPLC I-Class chromatography system coupled to an electrospray ionization source on a Waters Xevo TQ-XS triple quadrupole mass spectrometer detector (Milford, MA, USA). The mass detector was using the Multiple Reaction Monitoring (MRM) mode for data acquisition. The lipid panel was selected during method development based on screening PEx samples with targeted MRMs from Waters LipidQuan MRM database. An addition, untargeted scanning experiments for lipid class head groups was done using neutral loss scans and precursor ion scans, to verify that no major lipid species was missed. The most abundant lipids in each class were selected for further evaluation. A fit for purpose validation was done for the selected lipid panel to verify extraction, linearity repeatability for the amount of PEx mass that is used in the study (around 60 ng of PEx sample). Lipid annotations are based on the chromatographic retention times, molecular mass and fragmentation pattern in the mass spectrometer.

Each sample was analysed using two different chromatography methods, Hydrophilic Interaction Liquid Chromatography (HILIC) and reverse phase (RP), the methods were run from the same sample vial but different days. PC lipids are the major class of lipids in the sample. To minimize within lipid class interferences of this major lipid class they were separated with reverse phase chromatography before the mass spectrometer. With reverse phase chromatography, PC species are separated based on the fatty acyl chain length, number of double bonds and their position. The low abundant lipid classes were separated by a HILIC amide chromatography where the lipid classes are separated by the head group that defines the class. With the HILIC amide method interferences between lipid classes can be avoided as the lipid classes are fully separated by the elution time.

Quantification was based on the peak area of the analyte divided by the peak area of the internal standard, the response factor. The response factor was multiplied with the mol of internal standard spiked to the sample. One internal standard per lipid class was used to adjust for the extraction variability. Good linearity and precision were evaluated during method development and confirmed by the QC samples included in the run. The QC samples are prepared by spiking a reference sample of lung surfactant extracted from pooled BAL samples onto a sampling membrane to simulate a PEx sample with known lipid composition at different levels. QC samples were used to verify extraction, linearity and precision. For a large lipid panel in a screening method, it is not feasible to purchase a standard for each compound, but good selectivity, precision and linearity are sufficient for comparing samples analyzed in the same run with the same method. The amount of collected PEx sample was approximately the same but has a larger uncertainty than the chemical analysis (the median RSD% for the lipids is around 10% including the extraction and analysis). For this reason, each lipid was expressed as a relative amount expressed as a mol% $\left(mol\%=\frac{\text{analyte\textbackslash{}\_mol}}{\text{lipid\textbackslash{}\_sum\textbackslash{}\_mol}}\right)$ rather than normalized to the sampled PEx mass. Type II isotope correction (+2 isotope of one lipid can be isobaric with another lipid species with one double bond less) was done for the PE/PG/PI class of lipids as this effect varies with sample composition, and correction factors are calculated for each sample. Type II isotope correction factors were calculated with the online correction tool LICAR, which is described by Gao et al. [24].

Due to a small amount of exhaled particles (<80 ng) collected from some of the individuals (N = 8), there was not enough material available for the lipid analysis in those subjects. Therefore, we were able to analyse lipid profiles totally in 60 samples; 23 cases at baseline; 22 cases at follow-up, with 19 matched cases between baseline and follow-up; and 15 healthy controls.

### SOMAscan analysis and processing of data

SOMAscan analyses of PEx samples have been previously described in detail [15,25]. Proteins were extracted from the PEx substrate by transferring one half of the PTFE membrane to protein LoBind Eppendorf tubes and adding the sample buffer (SomaLogic SB17 buffer with addition of Tween-20 to 0.8% final concentration). The volume of sample buffer was adjusted to reach the final concentration of PEx at 0.33 µg/ml in all samples to normalize for the differences in the collected amount of PEx. After addition of sample buffer, the sample tubes were briefly vortexed and placed in a thermal rotary shaker for 45 min at 30°C and 1400 rpm. After incubation on the thermal shaker, 112 µL of protein extract was transferred to Matrix™ 500 μL ScrewTop Tubes (ThermoFisher Scientific, Waltham, Massachusetts, US) and stored at −20°C prior to being sent to SomaLogic (Boulder, Co, USA) for analysis. Blank samples were made by extraction of the material on PTFE membranes subjected to the same sampling and sample preparation procedure as all other samples, omitting the breathing manoeuvre. Four dilution samples were made by extracting the material on a PTFE membrane sampled from one individual and then diluting the extracted sample with four different buffer volumes to obtain different concentrations of PEx. SOMAscan data was reported in relative fluorescent units (RFU). The correlation of the coefficient of determination (R^2^) was calculated for each protein analyte based on three dilution samples. The limit of detection (LOD) was calculated as the mean plus three standard deviations based on six blank samples. Proteins with R2 > 80% and RFU values>LOD in more than 50% of the samples were considered for further analyses.

Due to lab error, 17 samples were lost during the analyses and therefore, 10 samples from healthy controls as well as 22 samples from cases at baseline and 17 samples at follow-up (with 13 matched cases between baseline and follow-up) were analysed.

### Statistivcal analysis

Statistical analyses of the protein and lipid data were performed using Qlucore Omics Explorer 3.8 software, Qlucore AB, Lund, Sweden). SOMAscan data and lipid data were log_2_ transformed before the analysis due to skewness of the data to achieve the distribution closer to normal. Independent samples t-test in Qlucore Omics Explorer was used to determine differences in protein and lipid abundances between the cases and controls, and a paired t-test was used for repeated measurements in COVID-19 cases. Pearson correlation test was used to assess correlations between the clinical data and the protein and lipid data. Correction for multiple testing (q-value) has been performed using the Benjamini-Hochberg method. Results with p-values <0.05 and q-values <0.30 were considered statistically significant. Descriptive statistics on clinical and demographic data were analysed using IBM SPSS Statistics for Windows, version 29 (IBM Corp., Armonk, N.Y., USA) where Mann-Whitney test for continuous clinical data was used, Chi -square test for categorical data, and related-samples Wilcoxon Signed Rank Test for repeated measurements.

## Results

### Clinical data

The demographic characteristics and the clinical data of the subjects included in the study are shown in Table 1. In general, these were young adults with normal BMI. Ten cases (34%) and one control (6%) were former smokers. There were no current smokers. The most common symptoms among cases at baseline were runny nose, cough, and fever, as shown in Table 2. Six subjects also reported chest pain, and eight subjects had difficulty breathing/shortness of breath (Table 2).

**Table 1:** General characteristics and clinical data of the subjects included in the study.

	COVID-19 cases			Healthy controls	p-value (q-value)	
	Baseline	Follow-up	p-value (q-value)	(n = 17)	Controls vs cases at baseline	Controls vs cases at follow-up
	(n = 29)	(n = 22)				
Females (%)	17 (59)			9 (53)	0.708 (0.819)	
Age; years	39 (32, 59)			42 (29, 60)	0.767 (0.819)	
BMI; kg/m^2^	22.5 (20.7, 25.3)			23.9 (20.4, 25.5)	0.707 (0.819)	
Former smokers; n (%)	10 (34)			1 (6)		
Asthma; n (%)	2 (7)			1 (6)		
Vaccinated against Covid-19; n (%)	13 (45)	19 (86)	0.002 (0.011)	17 (100)	0.001 (0.014)	0.113 (0.249)
mMRC ≥ 2; n (%)	12 (41)	1 (5)	0.004 (0.015)	1 (6)	0.010 (0.028)	0.878 (0.966)
Days since symptom onset	8 (5, 12)			NA		
Days since positive PCR-test	5 (2, 7)			NA		
Days between visit 1 and 2	NA	192 (111, 216)		NA		
**Pulmonary function**						
R_5;_ cmH_2_O.s/L	3.01 (2.48, 4.06)	2.84 (2.34, 3.52)	0.108 (0.170)	2.44 (1.78, 2.92)	0.007 (0.025)	0.087 (0.240)
R_5_ z-score	0.66 (−0.34, 1.66)	0.22 (−0.39, 1.03)	0.072 (0.132)	0.12 (−0.65, 0.42)	0.039 (0.078)	0.319 (0.501)
R_5-20_; cmH_2_O.s/L	0.11 (−0.14, 0.57)	−0.02 (−0.16, 0.50)	0.158 (0.205)	−0.02 (−0.19, 0.06)	0.038 (0.078)	0.221 (0.405)
R_5-20_ z-score	0.68 (−0.03, 1.76)	0.39 (−0.27, 1.41)	0.168 (0.205)	0.30 (0.15, 0.53)	0.086 (0.151)	0.492 (0.645)
AX; cmH_2_O/L	4.24 (1.89, 8.26)	2.85 (2.11, 4.83)	0.033 (0.073)	1.43 (1.02, 2.77)	0.002 (0.014)	0.025 (0.138)
AX z-score	1.23 (0.23, 2.03)	0.60 (−0.02, 1.40)	0.010 (0.028)	0.04 (−0.29, 0.69)	0.003 (0.014)	0.081 (0.240)
**Exhaled particles**						
PEx per liter of exhaled air; kN/L	13.65 (7.58, 24.59)	15.29 (8.90, 27.63)	0.848 (0.876)	23.92 (6.55, 38.44)	0.433 (0.674)	0.528 (0.645)
PEx per breath; kN/breath	23.70 (16.04, 65.28)	33.81 (22.78, 63.22)	0.876 (0.876)	47.80 (16.81, 63.90)	0.523 (0.732)	1.000 (1.000)
Particle mean mass; pg	0.25 (0.21, 0.28)	0.21 (0.18, 0.24)	0.001 (0.011)	0.25 (0.23, 0.29)	0.819 (0.819)	0.002 (0.022)

Median values and IQR are presented, unless specified otherwise. Mann-Whitney test was used for two group comparisons of continuous data and Chi-square test for categorical data. Related-samples Wilcoxon Signed Rank Test was used for comparison between cases at baseline and follow-up.

**Table 2:** Symptoms reported by COVID-19 cases at baseline and at follow-up.

Symptoms	n (%)
**Baseline (n = 29)**	
Chest pain	6 (21)
Difficulty breathing or shortness of breath	8 (28)
Cough	23 (79)
Fever	22 (76)
Runny nose	25 (86)
Blocked nose	23 (79)
Loss of smell and taste	21 (72)
Wheezing	5 (17)
**Follow-up (n = 22)**	
Fever episodes	1 (5)
Dry cough	2 (9)
Productive cough	3 (14)
Difficulty breathing or shortness of breath	5 (23)
Dizziness	2 (9)
Fatigue	7 (32)
Weight loss	2 (9)

At baseline, cases had more dyspnoea, defined as mMRC ≥ 2, as compared to healthy controls, as well as significantly increased total airway resistance (R_5_), peripheral airway resistance (R_5-20_) and reactance area (AX) (Table 1).

At follow-up, the most common newly debuted symptom, not present before COVID-19, was fatigue (Table 2). Five subjects reported difficulty breathing/shortness of breath and another three reported persistent cough. At follow-up, subjects had significantly lower AX and AX z-score as compared to baseline (Table 1).

When comparing cases at follow-up with healthy controls, there was no significant differences either in the small airway function or mMRC score.

### Lipid profile

**Fig 1:**
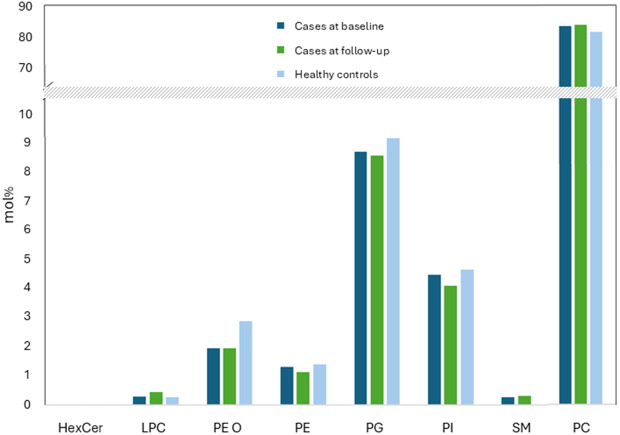
Distribution of average relative amounts of lipid classes. Expressed as mol% in cases at baseline and follow-up and healthy controls.

In all, 95 lipid species were identified in exhaled particles, belonging to eight lipid classes. A list of all lipid species identified is available in S1 Table. The distributions of average relative amounts of lipid classes for cases at baseline and follow-up and for healthy controls are shown in Fig 1.

### Baseline

No significant differences were observed in relative amounts of lipid classes between cases at baseline and controls. Significant negative correlation was observed between PE(O) class and R_5−20_ z-score (R = −0.44, p = 0.04, q = 0.29) among cases (S2 Table).

**Fig 2:**
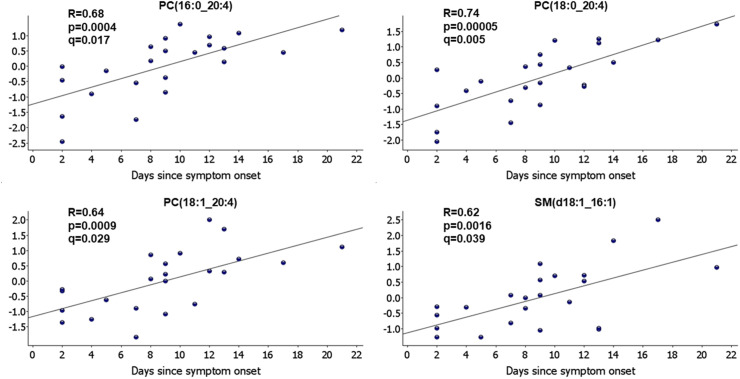
Correlation between relative amounts of lipid species and number of days since the onset of symptoms among COVID-19 cases. Only lipid species with R > 0.60 are displayed. Y-axis shows normalized amounts of lipid species (log_2_ transformation and normalization to mean 0 and variance 1).

Relative amounts of 13 lipid species were significantly different between cases and controls at baseline (Table 3). Relative amounts of 23 lipid species also significantly correlated with number of days since symptom onset (|R| between 0.43 and 0.74, p < 0.05 and q ≤ 0.176) (Fig 2). No significant correlation was observed between the amounts of lipid species and R_5-20_, AX and R_5_ z-score among cases at baseline (S2 Table).

**Table 3:** Differentially abundant lipid species in COVID-19 cases at baseline and at follow-up as compared to healthy controls.

	COVID-19 at baseline vs Healthy controls			COVID-19 at follow-up vs Healthy controls			COVID-19 at baseline vs at follow-up		
Lipid species	p-value	q-value	Difference	p-value	q-value	Difference	p-value	q-value	Difference
PC(14:0_18:1)	0.319	0.705	−0.04	**0.033**	**0.155**	**−0.08**	**0.016**	**0.207**	**−0.04**
PC(14:0_18:2)	**0.035**	**0.266**	**−0.02**	**0.048**	**0.190**	**−0.02**	0.969	0.986	0.00
PC(15:0_16:0)	0.186	0.618	0.09	**0.022**	**0.126**	**0.15**	0.369	0.558	0.03
PC(16:0_8:0;O)	**0.003**	**0.192**	**0.01**	0.060	0.205	0.01	0.600	0.740	0.00
PC(16:0/16:0)	0.158	0.601	0.06	**0.023**	**0.126**	**0.09**	**0.030**	**0.207**	**0.05**
PC(16:0_17:0)	0.512	0.771	0.03	**0.025**	**0.131**	**0.13**	**0.046**	**0.221**	**0.05**
PC(16:0_18:1)	0.943	0.977	0.00	0.290	0.552	−0.07	**0.036**	**0.213**	**−0.09**
PC(16:0_18:2)	**0.036**	**0.266**	**−0.12**	**0.003**	**0.051**	**−0.21**	0.096	0.318	−0.08
PC(16:0_18:3)_2*	0.687	0.907	−0.01	**0.043**	**0.181**	**−0.03**	**0.015**	**0.207**	**−0.03**
PC(16:0_20:4)	0.428	0.767	−0.02	0.059	0.205	−0.04	**0.029**	**0.207**	**−0.03**
PC(16:0_20:5)	**0.015**	**0.192**	**−0.01**	0.406	0.653	−0.01	0.220	0.499	0.01
PC(16:0_22:6)	**0.016**	**0.192**	**−0.01**	0.310	0.577	0.00	0.129	0.365	0.00
PC(O-16:0/16:0)	**0.018**	**0.192**	**0.03**	0.087	0.253	0.02	0.142	0.375	−0.02
PC(P-16:0/16:0)	**0.007**	**0.192**	**−0.04**	0.091	0.253	−0.03	0.624	0.759	0.01
PC(16:1_18:0)	0.946	0.977	0.00	0.214	0.452	−0.02	**0.029**	**0.207**	**−0.03**
PC(16:1_18:2)	0.095	0.476	−0.02	**0.015**	**0.121**	**−0.03**	0.100	0.318	−0.01
PC(16:0_7:0;O)	**0.027**	**0.229**	**0.04**	**0.004**	**0.054**	**0.06**	0.112	0.336	0.02
PC(16:0_9:0;O)	**0.009**	**0.192**	**0.22**	**0.002**	**0.051**	**0.26**	0.271	0.514	0.07
PC(18:0_18:1)	0.477	0.771	−0.03	0.187	0.412	−0.07	**0.041**	**0.217**	**−0.06**
PC(18:0_18:2)	**0.009**	**0.192**	**−0.11**	**0.003**	**0.051**	**−0.14**	0.096	0.318	−0.04
PC(18:0_20:4)	0.150	0.594	−0.01	**0.041**	**0.181**	**−0.02**	0.205	0.485	−0.01
PC(18:1_18:2)	0.109	0.517	−0.11	**0.015**	**0.121**	**−0.17**	0.250	0.505	−0.06
PC(18:1_20:4)	0.145	0.594	−0.01	**0.006**	**0.054**	**−0.01**	0.098	0.318	−0.01
PC(18:2/18:2)	**0.025**	**0.229**	**−0.09**	**0.018**	**0.126**	**−0.10**	0.795	0.853	0.01
PC(18:0_9:0;O)	0.052	0.355	0.04	**0.020**	**0.126**	**0.04**	0.732	0.814	0.01
PE(16:0_16:1)	0.404	0.767	0.00	0.438	0.667	0.00	**0.026**	**0.207**	**−0.01**
PE(16:0_18:1)	0.675	0.903	−0.01	**0.044**	**0.181**	**−0.03**	**0.008**	**0.207**	**−0.03**
PE(16:0_18:2)	0.961	0.977	0.00	**0.000**	**0.016**	**−0.03**	**0.015**	**0.207**	**−0.02**
PE(16:1_18:1)	0.737	0.922	0.00	**0.021**	**0.126**	**−0.01**	**0.010**	**0.207**	**−0.01**
PE(18:0_18:1)	0.652	0.900	−0.01	0.286	0.552	−0.02	**0.038**	**0.213**	**−0.02**
PE(18:0_18:2)	**0.016**	**0.192**	**−0.02**	**0.003**	**0.051**	**−0.03**	0.067	0.279	−0.01
PE(18:0_20:4)	0.638	0.900	0.00	0.269	0.532	0.00	**0.005**	**0.207**	**−0.01**
PE(18:1/18:1)	0.241	0.651	−0.03	**0.005**	**0.054**	**−0.09**	**0.016**	**0.207**	**−0.07**
PE(18:1_18:2)	**0.016**	**0.192**	**−0.04**	**0.001**	**0.028**	**−0.07**	0.087	0.318	−0.03
PE(18:1_20:0)	0.413	0.767	0.00	**0.005**	**0.054**	**−0.02**	**0.017**	**0.207**	**−0.01**
PG(18:0_20:4)	0.180	0.618	−0.01	**0.029**	**0.143**	**−0.01**	**0.022**	**0.207**	**−0.01**
PI(16:0_18:1)	0.360	0.760	0.05	0.668	0.824	−0.02	**0.028**	**0.207**	**−0.05**
PI(18:0_20:4)	0.964	0.977	0.00	0.137	0.318	−0.02	**0.037**	**0.213**	**−0.02**
SM(32:2;O2)	0.083	0.456	0.00	0.664	0.824	0.00	**0.047**	**0.221**	**0.00**

*When there are isomers with results in two peaks, the second peak is annotated with an ending of _2.

Difference is calculated based on the log_2_ data.

### Follow-up

A significantly lower relative amount of PE (p = 0.002, q = 0.016) was observed in cases at follow-up as compared to controls. Additionally, a significant decrease in PE (p = 0.016, q = 0.126) from baseline to follow-up was observed in cases. PE also positively correlated with R_5_ z-score (R = 0.49, p = 0.02, q = 0.15) in cases at follow-up (S3 Table).

The relative amounts of 24 lipid species were found to be significantly different between cases at follow-up and healthy controls (Table 3). Comparing the lipid species between cases at both visits, changes in the amounts of 20 lipid species were observed (Table 3). PG(16:1_18:0) correlated significantly with R_5-20_ z-score (R = 0.67, p = 0.001, q = 0.06) at follow-up (S2 Table).

### Protein profile

46 proteins fulfilled inclusion criteria and were included in further analyses (S3 Table). When looking at the proteins that fulfilled the inclusion criteria in each separate group (cases at baseline and follow-up, healthy controls), the majority of proteins detected overlapped between the groups (S4 Table).

### Baseline

**Fig 3:**
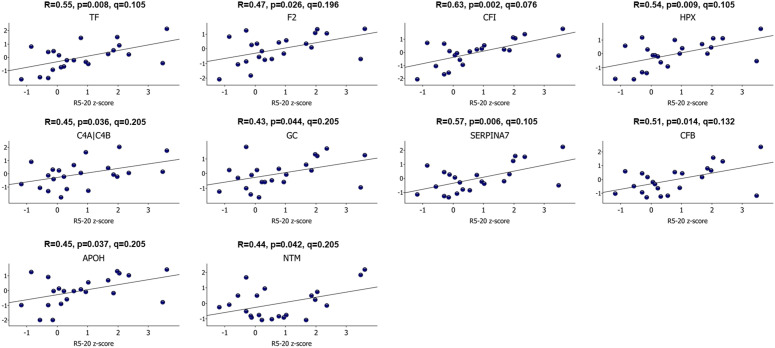
Correlation between protein abundances and R_5-20_ z-score among cases at baseline. Protein abundances, normalized to mean 0 and variance 1, are displayed on the y-axis. (*TF, Serotransferrin; F2, Prothrombin; CFI, Complement factor I; HPX, Hemopexin; C4A/C4B, Complement C4b; GC, Vitamin D-binding protein; SERPINA7, Thyroxine-binding globulin; CFB, Complement factor B; APOH, Beta-2-glycoprotein 1; NTM, Neurotrimin*).

When comparing protein abundances between the cases and controls at baseline, no differentially abundant proteins were found. Among cases, the abundance of 3 proteins (ephrin-A1, interleukin-6 receptor subunit beta, beta-1,4-galactosyltransferase 1) positively correlated with number of days since symptom onset, and the abundance of 7 proteins correlated negatively (antithrombin-III, beta-2-glycoprotein 1, lumican, hemopexin, prothrombin, vitamin D-binding protein, haptoglobin isoform 2). Abundances of 10 proteins (serotransferrin, prothrombin, complement factor I, hemopexin, complement C4b, vitamin D-binding protein, thyroxine-binding globulin, complement factor B, beta-2-glycoprotein 1, neurotrimin) significantly correlated with R_5-R20_ z-score, one of the markers of small airway dysfunction (Fig 3), however no significant correlation was observed between protein abundances and the AX z-score or the R_5_ z-score (S2 Table).

### Follow-up

No differentially abundant proteins were found between cases at follow-up and healthy controls. Additionally, among the 13 paired cases between baseline and follow-up, no significant changes in the protein abundances were found in cases between the two visits. At follow-up, no significant correlations were observed between AOS variables and protein abundances (S2 Table).

## Discussion

This study demonstrates that even mild to moderate COVID-19 exerts significant effects on the pulmonary surfactant phospholipid composition and causes small airway dysfunction. Mild to moderate SARS-CoV-2 infection seems to cause an increase in the relative amounts of oxidized lipids in the respiratory tract lining fluid of small airways and a decrease in the relative amounts of phospholipids with a linoleic acid component. These changes appear to persist over time as they were present months after an acute infection. Moreover, during acute infection, abundances of several acute phase proteins were correlated with small airway resistance, suggesting an increased inflammation in the small airways in patients with mild to moderate COVID-19.

Subjects with an ongoing SARS-CoV-2 infection exhibited more dyspnea and had higher total airway resistance (R5), small airway resistance (R_5-20_) and the reactance area, i.e., a measure of airway stiffness (AX), but the values were still within normal range in most cases. The AX (only absolute value, not z-score) remained the only variable that was found to be significantly higher in cases at follow-up as compared with controls. A study on patients with mild COVID-19 by Tamminen et al. [26] also reported increased small airway resistance and poorer lung elasticity in these patients during the acute phase as compared to healthy controls and patients with other respiratory infections. However, they did not observe any differences in the small airway function between these groups at a two-month follow-up [26]. Similarly, other follow-up studies on patients with a prior SARS-CoV-2 infection have not found any differences in lung function with spirometry, impulse oscillometry and diffusing capacity for carbon monoxide (DLCO) as compared to controls, despite some of these patients reporting persistent respiratory symptoms [4,27]. A study by Kjellberg et al. on subjects with a prior mild COVID-19 has, however, shown that ventilation inhomogeneity measured with multiple breath washout is present in half of the cases on average 15 months after acute infection and that lingering breathing difficulties are associated with increased S_acin_, an index representing ventilation heterogeneity at the entrance to the acinar zone [4].

It has been previously reported that severe COVID-19 exerts significant effects on the pulmonary surfactant and that the complement and kallikrein-kinin activation are some of the main drivers of inflammation in severe disease [13,14]. Due to the invasiveness of other methods to sample the small airway RTLF, such as bronchoalveolar lavage, studying patients with a mild to moderate infection is inherently difficult, and therefore most previous studies on the effects of SARS-CoV-2 on the pulmonary surfactant have been made on hospitalized patients with severe infection and/or ARDS. Due to the non-invasiveness of the PExA method to sample the biological material from the RTLF of small airways, we were able to study patients with a milder form of the disease and have demonstrated that the changes in the pulmonary surfactant can already be observed in mild to moderate COVID-19. Previous studies on the composition of these exhaled particles have been made and support that the origin of these particles is in small airways [28,29] and comparisons of their lipid and protein profiles with bronchoalveolar lavage fluid (BALF) have shown high similarities in the composition [12,15,28].

We observed that COVID-19 exerts significant alterations in the RTLF lipid profile, however, these changes were most prominent months after the acute infection. When looking at the lipid species, increased proportions of oxidized lipids (PC(16:0_7:0;O), PC(16:0_8:0;O), PC(16:0_9:0;O), PC(18:0_9:0;O)) were seen in COVID-19 mainly during the acute infection, but also at follow-up. Oxidized phospholipids are produced during oxidative stress as a part of the host-pathogen response and accumulate in inflamed tissues, where they have a pro-inflammatory role and induce coagulation by modulating the expression of coagulation factors [30]. Our results suggest that they also play an important role in the SARS-CoV-2 infection and that these changes may persist over time. Increased phospholipid oxidation could also contribute to COVID-19-related thromboinflammation. Furthermore, a decrease in lipid species with an 18:2 component, i.e., linoleic acid, was observed in COVID-19 and these changes also appear to persist over time. Linoleic acid is a polyunsaturated fatty acid and is considered proinflammatory as it is a precursor to arachidonic acid, which is a precursor for inflammatory mediators such as prostaglandins and leukotrienes [31]. That is, a decrease in lipid species with the linoleic acid could indicate the consumption due to the production of proinflammatory mediators in the respiratory epithelium. This represents a potential new target mechanism which should be studied further.

In the current study we found no significant differences in the RTLF protein profiles between patients and healthy controls. In contrast, a study by Hirdman et al. [11] reported 26 differentially expressed proteins in the RTLF in patients hospitalized with COVID-19. These proteins mainly belonged to the innate immune system and neutrophil and platelet degranulation. The same method (PExA method) was used to sample the RTLF in their study, however, the study population differed as compared to ours, as they included patients with a more severe COVID-19 in need of hospitalization, where changes in the protein profile may be more pronounced as compared to milder forms of the disease.

The abundances of several proteins also correlated with number of days since the onset of symptoms. Ephrin-A1, which plays a role in inflammation, particularly in the lungs, and regulates endothelial cell permeability, was highest in cases that were examined at the beginning of the disease and appeared to decrease with the number of days since the onset of symptoms. Increased levels of ephrin-A1 in blood and their association with the clinical outcome have been previously described in COVID-19 [32]. In contrast, we observed that the abundance of antithrombin III, which inhibits coagulation and has a potent anti-inflammatory activity [33], appeared to increase with the number of days since the onset of the disease. The same was observed for beta-2-glycoprotein 1, a protein with the ability to both up- or down-regulate the complement and coagulation systems [34], as well as hemopexin, prothrombin and haptoglobin. We also observed that several proteins (CFI, complement factor I; SERPINA7, thyroxine-binding globulin; TF, serotransferrin; HPX, hemopexin; CFB, complement factor B; F2, prothrombin) significantly correlated with the R_5−20_ z-score, one of the indices of small airway dysfunction. Prothrombin has been previously found to be increased in BALF of COVID-19 samples [35]. In our study, the abundance of prothrombin increased with increasing small airway dysfunction and time since symptom onset in subjects with COVID-19, suggesting an increased risk of fibrin deposition and clot formation. CFI, CFB and HPX are acute phase proteins, and their concentrations increase during inflammation [36–38]. CFI acts as a regulator of complement activation and inhibits all three complement activation pathways [36]. CFB is a specific marker of alternative pathway activation [39]. We found both CFI and CFB to be increased in patients with COVID-19 and small airway dysfunction. This suggests that the changes in the small airways in these patients could be due to increased inflammation caused by the infection and the inflammation is driven by complement activation. An increase in circulatory CFB in COVID-19 and its relation to disease severity and inflammatory markers has been reported previously [14,40]. A trend toward an increase in plasma HPX in patients with COVID-19 has been observed previously, however no correlation to clinical and laboratory markers of disease severity has been found [41]. SARS-CoV-2 can induce hemolysis through inflammation, complement activation and autoantibodies as well as through COVID-19-specific mechanisms, such as direct destruction of the red blood cells by the virus and autoantibody-induced hemolysis against viral products. HPX has an anti-inflammatory effect by clearing hemolysis-related degradation products that induce inflammation and oxidative stress as well as acting as an inhibitor of hemolysis-induced complement activation and therefore has been proposed as a therapeutic agent in COVID-19 [42].

The knowledge about the local effects of a mild SARS-CoV-2 infection on the protein and lipid profiles in the small airways is limited, as most studies have been performed on patients with a severe disease, and therefore this exploratory study is, to our knowledge, one of the first reporting the local changes in both lipid and protein profiles in the RTLF of mild to moderate COVID-19. However, several limitations need to be considered. First, the sample size is small and was furthermore decreased for the analyses of protein data as one-third of our samples were destroyed due to a lab error at SomaLogic. Second, only small amounts of exhaled particles were collected from some individuals and since the protein analyses were prioritized, we do not have lipid data available for all the subjects included in the study. Third, due to the small sample size and exploratory nature of the study, a more inclusive approach was taken, and the differences were identified as significant at p < 0.05 and q < 0.30. Fourth, the time periods from symptom onset to sampling varies between subjects with COVID-19 as well as the time period between the baseline and the follow-up. Although we have planned to examine all subjects with COVID-19 three months after the first visit, that was difficult due to practical issues. Fifth, during the time the study took place, three SARS-CoV-2 variants of concern (VOC) were dominant in Sweden (Alpha, Delta and Omicron) and it has been shown previously that the biomarkers differ between the VOCs [43]. In our study we were unfortunately not able to characterize the different VOCs. Last, both never smokers and former smokers were included in the study. Ten subjects with COVID-19 were former smokers and only one control. We have decided to include former smokers as we have seen in our previous studies that the small airway protein profiles and the amounts of two major lipids, DPPC and POPC (palmitoyloleoylphosphatidylcholine), are similar between former and never smokers [15,44].

## Conclusion

The observed alterations in the surfactant phospholipids in the RTLF indicate that surfactant homeostasis is affected already in a less severe illness, and these changes appear to persist over time as they were also observed months after acute infection. Furthermore, the correlation between small airway function and the abundance of several acute phase proteins in the RTLF suggests the infection may cause small airway inflammation, resulting in small airway dysfunction.

## Supporting information

S1 Fig Flow chart on the inclusion and drop out of patients. (TIF)

S1 Table List of all the lipid species analysed and included in the study. (PDF)

S2 Table Correlations between lipids and proteins and indices of small airway function. (PDF)

S3 Table List of proteins included in the study. (PDF)

S4 Table List of proteins detected in more than 50% of all samples for each group separately. (COVID-19 cases at baseline and follow-up and healthy controls).
(PDF)

S5 Dataset The dataset used and analysed during the current study. (PDF)

## Abbreviations

COVID-19: Coronavirus Disease 2019
SARS-CoV-2: Severe acute respiratory syndrome coronavirus 2
ARDS: acute respiratory distress syndrome
PASC: post-acute sequalae of COVID-19
AT2: alveolar type II cells
PG: phosphatidylglycerols
PI: phosphatidylinositols
PC: phosphatidylcholine
SM: sphingomyelin
PE: phosphatidylethanolamine
PS: phosphatidylserine
HexCer: hexosylceramide
DPPC: dipalmitoylphosphatidylcholine
BALF: bronchoalveolar lavage fluid
RTLF: respiratory tract lining fluid
IQR: interquartile range
AOS: airwave oscillometry
PEx: exhaled particles
RFU: relative fluorescent units
DLCO: diffusing capacity of the lungs for carbon monoxide
IOS: impulse oscillometry
LOD: limit of detection
VOC: variant of concern.
