# Supplementary figures and images for: Changes in the pulmonary surfactant in patients with mild to moderate COVID-19
Source: PLoS One. 2025 Aug 7;20(8):e0325153. doi: 10.1371/journal.pone.0325153 (PMC12331066; doi:10.1371/journal.pone.0325153)

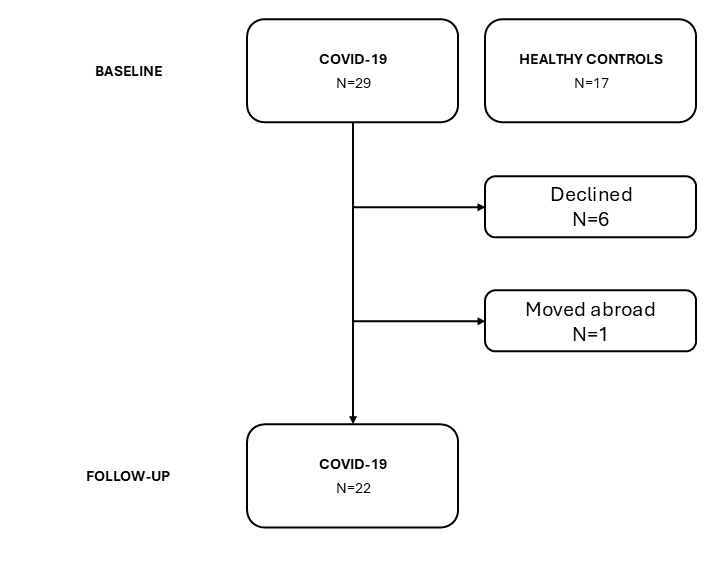

Supplement: S1 Fig — (TIF) [file pone.0325153.s001.tif]
