# Supplementary material for: Changes in the pulmonary surfactant in patients with mild to moderate COVID-19
Source: PLoS One. 2025 Aug 7;20(8):e0325153. doi: 10.1371/journal.pone.0325153 (PMC12331066; doi:10.1371/journal.pone.0325153)
Supplement: S1 Table — (PDF) [file pone.0325153.s002.pdf]

Lipid (Lipid Maps)	Class	Peak Name	Chromatography	Molecular Ion	MS1 *(m/z)*	MS2 *(m/z)*	Internal Standard
**HexCer(d18:1/16:0)**	HexCer	HexCer-d18_1-16_0-1	HILIC	$[M+H]^+$	700.6	264.3	xPG-15_0-18_1-288_1
**HexCer(d18:1/18:0)**	HexCer	HexCer-d18_1-18_0-1	HILIC	$[M+H]^+$	728.6	264.3	xPG-15_0-18_1-288_1
**HexCer(d18:1/20:0)**	HexCer	HexCer-d18_1-20_0-1	HILIC	$[M+H]^+$	756.6	264.3	xPG-15_0-18_1-288_1
**HexCer(d18:1/22:0)**	HexCer	HexCer-d18_1-22_0-1	HILIC	$[M+H]^+$	784.7	264.3	xPG-15_0-18_1-288_1
**PC(18:1(d7)_0:0)**	xLPC	xLPC-18_1-1	HILIC	$[M+H]^+$	529.4	184.1	IS_Avanti_SPLASH
**PC(18:1(d7)_0:0)**	xLPC	xLPC-18_1-2	HILIC	$[M+H]^+$	529.4	184.1	IS_Avanti_SPLASH
**PC(16:0_0:0)**	LPC	LPC-16_0-1	HILIC	$[M+H]^+$	496.3	184.1	xLPC-18_1-2
**PC(0:0_16:0)**	LPC	LPC-16_0-2	HILIC	$[M+H]^+$	496.3	184.1	xLPC-18_1-2
**PC(16:1_0:0)**	LPC	LPC-16_1-1	HILIC	$[M+H]^+$	494.3	184.1	xLPC-18_1-2
**PC(16:1_0:0)**	LPC	LPC-16_1-2	HILIC	$[M+H]^+$	494.3	184.1	xLPC-18_1-2
**PC(18:0_0:0)**	LPC	LPC-18_0-1	HILIC	$[M+H]^+$	524.4	184.1	xLPC-18_1-2
**PC(0:0_18:0)**	LPC	LPC-18_0-2	HILIC	$[M+H]^+$	524.4	184.1	xLPC-18_1-2
**PC(18:1_0:0)**	LPC	LPC-18_1-1	HILIC	$[M+H]^+$	522.4	184.1	xLPC-18_1-2
**PC(18:1_0:0)**	LPC	LPC-18_1-2	HILIC	$[M+H]^+$	522.4	184.1	xLPC-18_1-2
**PC(18:2_0:0)**	LPC	LPC-18_2-1	HILIC	$[M+H]^+$	520.3	184.1	xLPC-18_1-2
**PC(18:2_0:0)**	LPC	LPC-18_2-2	HILIC	$[M+H]^+$	520.3	184.1	xLPC-18_1-2
**PE(18:1(d7)_0:0)**	xPE	xLPE-18_1-1	HILIC	$[M+H]^+$	487.3	346.3	IS_Avanti_SPLASH
**PE(18:1(d7)_0:0)**	xPE	xLPE-18_1-2	HILIC	$[M+H]^+$	487.3	346.3	IS_Avanti_SPLASH
**PE (O-2:0_18:1)**	PE O-	PE O-2:0a/18:1	HILIC	$[M+H]^+$	508.3	367.3	xLPE-18_1-1
**PE (O-2:0_18:2)**	PE O-	PE O-2:0a/18:2	HILIC	$[M+H]^+$	506.3	365.3	xLPE-18_1-1
**PE (O-2:0_20:1)**	PE O-	PE O-2:0a/20:1	HILIC	$[M+H]^+$	536.4	395.4	xLPE-18_1-1
**PE (O-2:0_20:4)**	PE O-	PE O-2:0a/20:4	HILIC	$[M+H]^+$	530.3	389.3	xLPE-18_1-1
**PE(15:0/18:1(d7)**	xPE	xPE-15_0-18_1-288_1	HILIC	$[M-H]^-$	709.6	288.3	IS_Avanti_SPLASH
**PE(14:0_18:1)**	PE	PE-14_0-18_1-281_1	HILIC	$[M-H]^-$	688.5	281.2	xPE-15_0-18_1-288_1
**PE(16:0_16:1)**	PE	PE-16_0-16_1-253_1	HILIC	$[M-H]^-$	688.5	253.2	xPE-15_0-18_1-288_1
**PE(16:0_18:1)**	PE	PE-16_0-18_1-281_1	HILIC	$[M-H]^-$	716.5	281.2	xPE-15_0-18_1-288_1
**PE(16:0_18:2)**	PE	PE-16_0-18_2-279_1	HILIC	$[M-H]^-$	714.5	279.2	xPE-15_0-18_1-288_1
**PE(16:0_20:4)**	PE	PE-16_0-20_4-303_1	HILIC	$[M-H]^-$	738.5	303.2	xPE-15_0-18_1-288_1
**PE(16:1_18:0)**	PE	PE-16_1-18_0-283_1	HILIC	$[M-H]^-$	716.5	283.3	xPE-15_0-18_1-288_1
**PE(16:1_18:1)**	PE	PE-16_1-18_1-253_1	HILIC	$[M-H]^-$	714.5	253.2	xPE-15_0-18_1-288_1
**PE(18:0_18:1)**	PE	PE-18_0-18_1-281_1	HILIC	$[M-H]^-$	744.6	281.2	xPE-15_0-18_1-288_1
**PE(18:0_18:2)**	PE	PE-18_0-18_2-279_1	HILIC	$[M-H]^-$	742.5	279.2	xPE-15_0-18_1-288_1
**PE(18:0_20:4)**	PE	PE-18_0-20_4-303_1	HILIC	$[M-H]^-$	766.5	303.2	xPE-15_0-18_1-288_1
**PE(18:1_18:1)**	PE	PE-18_1-18_1-281_1	HILIC	$[M-H]^-$	742.5	281.2	xPE-15_0-18_1-288_1
**PE(18:1_18:2)**	PE	PE-18_1-18_2-279_1	HILIC	$[M-H]^-$	740.5	279.2	xPE-15_0-18_1-288_1
**PE(18:1_20:4)**	PE	PE-18_1-20_4-303_1	HILIC	$[M-H]^-$	764.5	303.2	xPE-15_0-18_1-288_1
**PG(15:0/18:1(d7))**	xPG	xPG-15_0-18_1-288_1	HILIC	$[M-H]^-$	740.6	288.3	IS_Avanti_SPLASH
**PG(14:0_16:0)**	PG	PG-14_0-16_0-227_1	HILIC	$[M-H]^-$	693.5	227.2	xPG-15_0-18_1-288_1
**PG(16:0_16:0)**	PG	PG-16_0-16_0-255_1	HILIC	$[M-H]^-$	721.5	255.2	xPG-15_0-18_1-288_1
**PG(16:0_16:1)**	PG	PG-16_0-16_1-253_1	HILIC	$[M-H]^-$	719.5	253.2	xPG-15_0-18_1-288_1
**PG(16:0_18:0)**	PG	PG-16_0-18_0-255_1	HILIC	$[M-H]^-$	749.5	255.2	xPG-15_0-18_1-288_1
**PG(16:0_18:1)**	PG	PG-16_0-18_1-281_1	HILIC	$[M-H]^-$	747.5	281.2	xPG-15_0-18_1-288_1
**PG(16:0_18:2)**	PG	PG-16_0-18_2-279_1	HILIC	$[M-H]^-$	745.5	279.2	xPG-15_0-18_1-288_1
**PG(16:1_18:0)**	PG	PG-16_1-18_0-253_1	HILIC	$[M-H]^-$	747.5	253.2	xPG-15_0-18_1-288_1
**PG(16:1_18:1)**	PG	PG-16_1-18_1-253_1	HILIC	$[M-H]^-$	745.5	253.2	xPG-15_0-18_1-288_1
**PG(18:0_18:1)**	PG	PG-18_0-18_1-281_1	HILIC	$[M-H]^-$	775.6	281.2	xPG-15_0-18_1-288_1
**PG(18:0_18:2)**	PG	PG-18_0-18_2-279_1	HILIC	$[M-H]^-$	773.5	279.2	xPG-15_0-18_1-288_1
**PG(18:0_20:4)**	PG	PG-18_0-20_4-303_1	HILIC	$[M-H]^-$	797.5	303.2	xPG-15_0-18_1-288_1

**PG(18:1_18:1)**	PG	PG-18_1-18_1-281_1	HILIC	$[M-H]^-$	773.5	281.2	xPG-15_0-18_1-288_1
**PG(18:1_18:2)**	PG	PG-18_1-18_2-279_1	HILIC	$[M-H]^-$	771.5	279.2	xPG-15_0-18_1-288_1
**PI(15:0/18:1(d7))**	xPI	xPI-15_0-18_1-288_1	HILIC	$[M-H]^-$	828.6	288.3	IS_Avanti_SPLASH
**PI(16:0_18:1)**	PI	PI-16_0-18_1-255_1	HILIC	$[M-H]^-$	835.5	255.2	xPI-15_0-18_1-288_1
**PI(18:0_18:1)**	PI	PI-18_0-18_1-283_1	HILIC	$[M-H]^-$	863.6	281.3	xPI-15_0-18_1-288_1
**PI(18:0_20:4)**	PI	PI-18_0-20_4-283_1	HILIC	$[M-H]^-$	885.6	283.3	xPI-15_0-18_1-288_1
**PI(18:1_18:1)**	PI	PI-18_1-18_1-281_1	HILIC	$[M-H]^-$	861.6	281.2	xPI-15_0-18_1-288_1
**SM(d18:1_18:1(d9))**	xSM	xSM-d18_1-18_1_1	HILIC	$[M+H]^+$	738.7	184.1	IS_Avanti_SPLASH
**SM(d18:1_12:0)**	SM	SM-d18_1-12_0-1	HILIC	$[M+H]^+$	647.5	184.1	xSM-d18_1-18_1_1
**SM(d18:1_14:0)**	SM	SM-d18_1-14_0-1	HILIC	$[M+H]^+$	675.5	184.1	xSM-d18_1-18_1_1
**SM(d18:1_14:1)**	SM	SM-d18_1-14_1-1	HILIC	$[M+H]^+$	673.5	184.1	xSM-d18_1-18_1_1
**SM(d18:1_16:0)**	SM	SM-d18_1-16_0-1	HILIC	$[M+H]^+$	703.6	184.1	xSM-d18_1-18_1_1
**SM(d18:1_16:1)**	SM	SM-d18_1-16_1-1	HILIC	$[M+H]^+$	701.6	184.1	xSM-d18_1-18_1_1
**SM(d18:1_17:0)**	SM	SM-d18_1-17_0-1	HILIC	$[M+H]^+$	717.6	184.1	xSM-d18_1-18_1_1
**SM(d18:1_18:0)**	SM	SM-d18_1-18_0-1	HILIC	$[M+H]^+$	731.6	184.1	xSM-d18_1-18_1_1
**SM(d18:1_18:1)**	SM	SM-d18_1-18_1-1	HILIC	$[M+H]^+$	729.6	184.1	xSM-d18_1-18_1_1
**PC(15:0/18:1(d7))**	xPC	xPCneg-15_0-18_1-288_1	RP	$[M+OAc]^-$	811.6	288.3	IS_Avanti_SPLASH
**PC(14:0_14:0)**	PC	PC 14_0-14_0	RP	$[M+OAc]^-$	736.5	227.2	xPCneg-15_0-18_1-288_1
**PC(14:0_16:0)**	PC	PC 14_0-16_0	RP	$[M+OAc]^-$	764.5	227.2	xPCneg-15_0-18_1-288_1
**PC(14:0_16:1)**	PC	PC 14_0-16_1	RP	$[M+OAc]^-$	762.5	253.2	xPCneg-15_0-18_1-288_1
**PC(14:0_18:0)**	PC	PC 14_0-18_0	RP	$[M+OAc]^-$	792.6	227.2	xPCneg-15_0-18_1-288_1
**PC(14:0_18:1)**	PC	PC 14_0-18_1	RP	$[M+OAc]^-$	790.6	281.2	xPCneg-15_0-18_1-288_1
**PC(14:0_18:2)**	PC	PC 14_0-18_2	RP	$[M+OAc]^-$	788.5	279.2	xPCneg-15_0-18_1-288_1
**PC(14:1_16:0)**	PC	PC 14_1-16_0	RP	$[M+OAc]^-$	762.5	225.2	xPCneg-15_0-18_1-288_1
**PC(15:0_16:0)**	PC	PC 15_0-16_0	RP	$[M+OAc]^-$	778.6	241.2	xPCneg-15_0-18_1-288_1
**PC(15:0_18:1)**	PC	PC 15_0-18_1	RP	$[M+OAc]^-$	804.6	241.2	xPCneg-15_0-18_1-288_1
**PC(16:0_16:0)**	PC	PC 16_0-16_0	RP	$[M+OAc]^-$	792.6	255.2	xPCneg-15_0-18_1-288_1
**PC(16:0_16:1)**	PC	PC 16_0-16_1	RP	$[M+OAc]^-$	790.6	253.2	xPCneg-15_0-18_1-288_1
**PC(16:0_17:0)**	PC	PC 16_0-17_0	RP	$[M+OAc]^-$	806.6	269.2	xPCneg-15_0-18_1-288_1
**PC(16:0_17:1)**	PC	PC 16_0-17_1 A	RP	$[M+OAc]^-$	804.6	267.2	xPCneg-15_0-18_1-288_1
**PC(16:0_17:1)**	PC	PC 16_0-17_1 B	RP	$[M+OAc]^-$	804.6	267.2	xPCneg-15_0-18_1-288_1
**PC(16:0_18:0)**	PC	PC 16_0-18_0	RP	$[M+OAc]^-$	820.6	255.2	xPCneg-15_0-18_1-288_1
**PC(16:0_18:1)**	PC	PC 16_0-18_1	RP	$[M+OAc]^-$	818.6	281.2	xPCneg-15_0-18_1-288_1
**PC(16:0_18:2)**	PC	PC 16_0-18_2	RP	$[M+OAc]^-$	816.6	279.2	xPCneg-15_0-18_1-288_1
**PC(16:0_18:3)**	PC	PC 16_0-18_3 A	RP	$[M+OAc]^-$	814.6	277.2	xPCneg-15_0-18_1-288_1
**PC(16:0_18:3)**	PC	PC 16_0-18_3 B	RP	$[M+OAc]^-$	814.6	277.2	xPCneg-15_0-18_1-288_1
**PC(16:0_20:4)**	PC	PC 16_0-20_4	RP	$[M+OAc]^-$	840.6	303.2	xPCneg-15_0-18_1-288_1
**PC(16:0_20:5)**	PC	PC 16_0-20_5	RP	$[M+OAc]^-$	838.6	301.2	xPCneg-15_0-18_1-288_1
**PC(16:0_22:6)**	PC	PC 16_0-22_6	RP	$[M+OAc]^-$	864.6	327.2	xPCneg-15_0-18_1-288_1
**PC(16:1_16:1)**	PC	PC 16_1-16_1	RP	$[M+OAc]^-$	788.5	253.2	xPCneg-15_0-18_1-288_1
**PC(16:1_18:0)**	PC	PC 16_1-18_0	RP	$[M+OAc]^-$	818.6	253.2	xPCneg-15_0-18_1-288_1
**PC(16:1_18:1)**	PC	PC 16_1-18_1	RP	$[M+OAc]^-$	816.6	253.2	xPCneg-15_0-18_1-288_1
**PC(16:1_18:2)**	PC	PC 16_1-18_2	RP	$[M+OAc]^-$	814.6	279.2	xPCneg-15_0-18_1-288_1
**PC(18:0_18:1)**	PC	PC 18_0-18_1	RP	$[M+OAc]^-$	846.6	281.2	xPCneg-15_0-18_1-288_1
**PC(18:0_18:2)**	PC	PC 18_0-18_2	RP	$[M+OAc]^-$	844.6	279.2	xPCneg-15_0-18_1-288_1
**PC(18:0_20:4)**	PC	PC 18_0-20_4	RP	$[M+OAc]^-$	868.6	303.2	xPCneg-15_0-18_1-288_1
**PC(18:1_18:1)**	PC	PC 18_1-18_1	RP	$[M+OAc]^-$	844.6	281.2	xPCneg-15_0-18_1-288_1
**PC(18:1_18:2)**	PC	PC 18_1-18_2	RP	$[M+OAc]^-$	842.6	279.2	xPCneg-15_0-18_1-288_1
**PC(18:1_20:4)**	PC	PC 18_1-20_4	RP	$[M+OAc]^-$	866.6	303.2	xPCneg-15_0-18_1-288_1

**PC(18:2_18:2)**	PC	PC 18_2-18_2	RP	$[M+OAc]^-$	840.6	279.233	xPCneg-15_0-18_1-288_1
**PC(O-16:0_16:0)**	PC O-	PC-16_0e-16_0	RP	$[M+OAc]^-$	778.6	255.2	xPCneg-15_0-18_1-288_1
**PC(P-16:0_16:0)**	PC P	PC-16_0p-16_0	RP	$[M+OAc]^-$	776.6	255.2	xPCneg-15_0-18_1-288_1
**PC(16:0_7:0;O)**	oxPC	PC16:0_7:0al	RP	$[M+OAc]^-$	680.4	143.0	xPCneg-15_0-18_1-288_1
**PC(16:0_8:0;O)**	oxPC	PC 16.0_8:0al	RP	$[M+OAc]^-$	694.4	157.0	xPCneg-15_0-18_1-288_1
**PC(16:0_9:0;O)**	oxPC	PC16:0_9:0al	RP	$[M+OAc]^-$	708.4	171.0	xPCneg-15_0-18_1-288_1
**PC(18:0_9:0;O)**	oxPC	PC18:0_9:0al	RP	$[M+OAc]^-$	736.4	171.0	xPCneg-15_0-18_1-288_1
