# Supplementary material for: Changes in the pulmonary surfactant in patients with mild to moderate COVID-19
Source: PLoS One. 2025 Aug 7;20(8):e0325153. doi: 10.1371/journal.pone.0325153 (PMC12331066; doi:10.1371/journal.pone.0325153)
Supplement: S2 Table — (PDF) [file pone.0325153.s003.pdf]

Table S2. Correlations between relative amounts of lipid classes and lipid species, as well as protein anundances, and indices of small airway function.

BASELINE	R5			R5 z-score			R5-20			R5-20 z-score			AX			AX z-score		
	p-value	q-value	R	p-value	q-value	R	p-value	q-value	R	p-value	q-value	R	p-value	q-value	R	p-value	q-value	R
**Lipid class**																		
HexCer	0.531	0.663	0.14	0.862	0.862	0.04	0.789	0.926	0.06	0.721	0.833	0.08	0.866	0.866	-0.04	0.521	0.902	-0.14
LPC	0.211	0.338	-0.27	0.260	0.519	-0.25	0.660	0.926	0.10	0.695	0.833	0.09	0.600	0.788	-0.12	0.795	0.902	-0.06
PE(O)	0.061	0.281	-0.40	0.087	0.347	-0.37	0.019	0.154	-0.48	0.036	0.288	-0.44	0.036	0.292	-0.44	0.088	0.704	-0.36
PC	0.586	0.663	0.12	0.838	0.862	-0.05	0.575	0.926	0.12	0.833	0.833	-0.05	0.494	0.788	0.15	0.902	0.902	-0.03
PE	0.663	0.663	-0.10	0.733	0.862	-0.08	0.889	0.926	-0.03	0.768	0.833	0.07	0.577	0.788	-0.12	0.891	0.902	-0.03
PG	0.142	0.338	-0.32	0.388	0.621	-0.19	0.926	0.926	-0.02	0.794	0.833	0.06	0.680	0.788	-0.09	0.595	0.902	0.12
PI	0.070	0.281	0.38	0.054	0.347	0.41	0.808	0.926	0.05	0.455	0.833	0.16	0.622	0.788	0.11	0.814	0.902	0.05
SM	0.196	0.338	0.28	0.187	0.498	0.29	0.421	0.926	0.18	0.397	0.833	0.19	0.689	0.788	0.09	0.528	0.902	0.14
**Lipid species**																		
HexCerd18_116_01	0.822	0.908	0.05	0.983	0.993	0.00	0.978	0.998	-0.01	0.989	0.989	0.00	0.670	0.974	-0.09	0.431	0.944	-0.17
HexCerd18_118_01	0.977	0.996	0.01	0.624	0.979	-0.11	0.789	0.998	0.06	0.731	0.986	0.08	0.678	0.974	-0.09	0.376	0.944	-0.19
HexCerd18_120_01	0.489	0.890	0.15	0.941	0.993	-0.02	0.554	0.998	0.13	0.417	0.986	0.18	0.856	0.974	0.04	0.785	0.944	-0.06
HexCerd18_122_01	0.352	0.859	0.20	0.566	0.979	0.13	0.676	0.998	0.09	0.470	0.986	0.16	0.796	0.974	0.06	0.737	0.944	-0.07
LPC16_01	0.673	0.890	-0.09	0.900	0.993	-0.03	0.270	0.998	0.24	0.172	0.986	0.29	0.767	0.974	0.07	0.255	0.944	0.25
LPC16_02	0.193	0.763	-0.28	0.175	0.969	-0.29	0.959	0.998	0.01	0.964	0.989	-0.01	0.422	0.974	-0.18	0.410	0.944	-0.18
LPC16_11	0.607	0.890	0.11	0.432	0.979	0.17	0.067	0.998	0.39	0.033	0.986	0.45	0.288	0.974	0.23	0.093	0.944	0.36
LPC16_12	0.478	0.890	0.16	0.743	0.993	0.07	0.025	0.998	0.46	0.059	0.986	0.40	0.084	0.974	0.37	0.123	0.944	0.33
LPC18_01	0.413	0.890	-0.18	0.628	0.979	-0.11	0.850	0.998	0.04	0.978	0.989	-0.01	0.781	0.974	-0.06	0.856	0.944	0.04
LPC18_02	0.328	0.841	-0.21	0.511	0.979	-0.14	0.809	0.998	-0.05	0.571	0.986	-0.12	0.762	0.974	-0.07	0.732	0.944	-0.08
LPC18_11	0.533	0.890	-0.14	0.756	0.993	-0.07	0.406	0.998	0.18	0.317	0.986	0.22	0.969	0.989	0.01	0.582	0.944	0.12
LPC18_12	0.377	0.883	-0.19	0.460	0.979	-0.16	0.302	0.998	0.22	0.350	0.986	0.20	0.852	0.974	0.04	0.535	0.944	0.14
LPC18_21	0.553	0.890	0.13	0.829	0.993	0.05	0.061	0.998	0.40	0.055	0.986	0.40	0.244	0.974	0.25	0.431	0.944	0.17
LPC18_22	0.586	0.890	-0.12	0.289	0.979	-0.23	0.175	0.998	0.29	0.231	0.986	0.26	0.846	0.974	0.04	0.992	0.992	0.00
PE O-2:0a/18:1	0.128	0.713	-0.33	0.140	0.969	-0.32	0.047	0.998	-0.42	0.065	0.986	-0.39	0.083	0.974	-0.37	0.175	0.944	-0.29
PE O-2:0a/18:2	0.061	0.713	-0.40	0.098	0.969	-0.35	0.017	0.998	-0.49	0.057	0.986	-0.40	0.063	0.974	-0.39	0.122	0.944	-0.33
PE O-2:0a/20:1	0.284	0.772	-0.23	0.432	0.979	-0.17	0.146	0.998	-0.31	0.191	0.986	-0.28	0.137	0.974	-0.32	0.189	0.944	-0.28
PE O-2:0a/20:4	0.049	0.713	-0.42	0.067	0.969	-0.39	0.175	0.998	-0.29	0.133	0.986	-0.32	0.216	0.974	-0.27	0.228	0.944	-0.26
PC14_014_0	0.522	0.890	-0.14	0.846	0.993	0.04	0.602	0.998	-0.11	0.835	0.989	-0.05	0.933	0.989	0.02	0.567	0.944	0.13
PC14_016_0	0.743	0.890	-0.07	0.829	0.993	0.05	0.342	0.998	-0.21	0.580	0.986	-0.12	0.732	0.974	-0.08	0.982	0.992	0.00
PC14_016_1	0.454	0.890	-0.16	0.600	0.979	-0.12	0.772	0.998	0.06	0.739	0.986	0.07	0.877	0.974	0.03	0.363	0.944	0.20
PC14_018_0	0.685	0.890	-0.09	0.843	0.993	0.04	0.992	0.998	0.00	0.818	0.986	0.05	0.729	0.974	0.08	0.547	0.944	0.13
PC14_018_1	0.080	0.713	-0.37	0.294	0.979	-0.23	0.802	0.998	-0.06	0.625	0.986	-0.11	0.996	0.996	0.00	0.314	0.944	0.22
PC14_018_2	0.157	0.713	-0.31	0.268	0.979	-0.24	0.984	0.998	0.00	0.789	0.986	-0.06	0.775	0.974	0.06	0.329	0.944	0.21
PC14_116_0	0.778	0.890	-0.06	0.975	0.993	-0.01	0.579	0.998	0.12	0.359	0.986	0.20	0.748	0.974	0.07	0.291	0.944	0.23
PC15_016_0	0.545	0.890	-0.13	0.591	0.979	-0.12	0.507	0.998	-0.15	0.615	0.986	-0.11	0.648	0.974	-0.10	0.940	0.971	-0.02
PC15_018_1	0.337	0.843	-0.21	0.442	0.979	-0.17	0.444	0.998	-0.17	0.179	0.986	-0.29	0.541	0.974	-0.13	0.925	0.971	-0.02
PC16.0_80al	0.446	0.890	0.17	0.957	0.993	-0.01	0.866	0.998	-0.04	0.507	0.986	-0.15	0.739	0.974	0.07	0.694	0.944	-0.09
PC160_70al	0.253	0.772	0.25	0.599	0.979	0.12	0.905	0.998	-0.03	0.597	0.986	-0.12	0.771	0.974	0.06	0.784	0.944	-0.06
PC160_90al	0.490	0.890	0.15	0.767	0.993	-0.07	0.610	0.998	0.11	0.894	0.989	-0.03	0.295	0.974	0.23	0.644	0.944	0.10
PC16_016_0	0.188	0.763	0.28	0.488	0.979	0.15	0.855	0.998	0.04	0.988	0.989	0.00	0.666	0.974	0.10	0.405	0.944	-0.18
PC16_016_1	0.712	0.890	0.08	0.893	0.993	0.03	0.461	0.998	0.16	0.482	0.986	0.15	0.970	0.989	-0.01	0.500	0.944	0.15
PC16_017_0	0.692	0.890	-0.09	0.374	0.979	-0.19	0.936	0.998	0.02	0.974	0.989	-0.01	0.857	0.974	-0.04	0.810	0.944	-0.05
PC16_017_1	0.753	0.890	-0.07	0.727	0.993	-0.08	0.599	0.998	0.12	0.683	0.986	0.09	0.771	0.974	0.06	0.482	0.944	0.15
PC16_017_11	0.522	0.890	-0.14	0.417	0.979	-0.18	0.711	0.998	-0.08	0.939	0.989	-0.02	0.795	0.974	-0.06	0.810	0.944	-0.05
PC16_018_0	0.633	0.890	0.11	0.749	0.993	0.07	0.348	0.998	0.21	0.487	0.986	0.15	0.276	0.974	0.24	0.581	0.944	0.12
PC16_018_1	0.141	0.713	-0.32	0.148	0.969	-0.31	0.501	0.998	0.15	0.895	0.989	0.03	0.794	0.974	-0.06	0.382	0.944	0.19
PC16_018_2	0.015	0.713	-0.50	0.072	0.969	-0.38	0.549	0.998	-0.13	0.613	0.986	-0.11	0.411	0.974	-0.18	0.865	0.944	0.04
PC16_018_3	0.502	0.890	-0.15	0.211	0.979	-0.27	0.627	0.998	-0.11	0.529	0.986	-0.14	0.723	0.974	-0.08	0.223	0.944	-0.26
PC16_018_31	0.840	0.917	-0.04	0.408	0.979	0.18	0.457	0.998	0.16	0.435	0.986	0.17	0.408	0.974	0.18	0.252	0.944	0.25
PC16_020_4	0.570	0.890	-0.12	0.950	0.993	0.01	0.150	0.998	0.31	0.249	0.986	0.25	0.418	0.974	0.18	0.113	0.944	0.34
PC16_020_5	0.224	0.763	-0.26	0.510	0.979	-0.14	0.832	0.998	-0.05	0.811	0.986	-0.05	0.586	0.974	-0.12	0.685	0.944	-0.09
PC16_022_6	0.606	0.890	-0.11	0.628	0.979	0.11	0.297	0.998	0.23	0.314	0.986	0.22	0.609	0.974	0.11	0.165	0.944	0.30
PC16_0e16_0	0.258	0.772	0.25	0.165	0.969	0.30	0.168	0.998	0.30	0.124	0.986	0.33	0.155	0.974	0.31	0.111	0.944	0.34
PC16_0p16_0	0.860	0.928	0.04	0.176	0.969	0.29	0.799	0.998	-0.06	0.818	0.986	0.05	0.766	0.974	0.07	0.429	0.944	0.17
PC16_116_1	0.988	0.996	0.00	0.767	0.993	-0.07	0.448	0.998	0.17	0.563	0.986	0.13	0.710	0.974	0.08	0.347	0.944	0.21
PC16_118_0	0.738	0.890	0.07	0.842	0.993	-0.04	0.190	0.998	0.28	0.317	0.986	0.22	0.516	0.974	0.14	0.392	0.944	0.19
PC16_118_1	0.571	0.890	-0.12	0.465	0.979	-0.16	0.934	0.998	0.02	0.722	0.986	-0.08	0.979	0.989	0.01	0.439	0.944	0.17
PC16_118_2	0.219	0.763	-0.27	0.178	0.969	-0.29	0.959	0.998	-0.01	0.803	0.986	-0.06	0.706	0.974	-0.08	0.669	0.944	0.09
PC180_90al	0.887	0.947	0.03	0.777	0.993	-0.06	0.792	0.998	0.06	0.819	0.986	-0.05	0.490	0.974	0.15	0.795	0.944	0.06
PC18_018_1	0.135	0.713	-0.32	0.361	0.979	-0.20	0.784	0.998	0.06	0.935	0.989	0.02	0.532	0.974	-0.14	0.913	0.971	0.02
PC18_018_2	0.054	0.713	-0.41	0.214	0.979	-0.27	0.649	0.998	-0.10	0.716	0.986	-0.08	0.366	0.974	-0.20	0.795	0.944	-0.06
PC18_020_4	0.721	0.890	-0.08	0.709	0.993	0.08	0.490	0.998	0.15	0.641	0.986	0.10	0.616	0.974	0.11	0.485	0.944	0.15
PC18_118_1	0.218	0.763	-0.27	0.258	0.979	-0.25	0.560	0.998	0.13	0.968	0.989	0.01	0.668	0.974	0.09	0.282	0.944	0.23
PC18_118_2	0.103	0.713	-0.35	0.170	0.969	-0.30	0.762	0.998	-0.07	0.578	0.986	-0.12	0.976	0.989	0.01	0.596	0.944	0.12
PC18_120_4	0.272	0.772	-0.24	0.597	0.979	-0.12	0.756	0.998	0.07	0.820	0.986	-0.05	0.826	0.974	0.05	0.655	0.944	0.10
PC18_218_2	0.740	0.890	0.07	0.932	0.993	-0.02	0.137	0.998	0.32	0.085	0.986	0.37	0.142	0.974	0.32	0.216	0.944	0.27
PE14_018_1281_1	0.996	0.996	0.00	0.320	0.979	0.22	0.843	0.998	-0.04	0.743	0.986	0.07	0.847	0.974	-0.04	0.434	0.944	0.17
PE16_016_1253_1	0.322	0.841	0.22	0.499	0.979	0.15	0.445	0.998	0.17	0.444	0.986	0.17	0.678	0.974	0.09	0.365	0.944	0.20
PE16_018_1281_1	0.558	0.890	0.13	0.682	0.993	0.09	0.881	0.998	0.03	0.593	0.986	0.12	0.690	0.974	-0.09	0.826	0.944	-0.05
PE16_018_2279_1	0.283	0.772	0.23	0.661	0.993	0.10	0.066	0.998	0.39	0.041	0.986	0.43	0.131	0.974	0.32	0.312	0.944	0.22
PE16_020_4303_1	0.655	0.890	-0.10	0.522	0.979	-0.14	0.725	0.998	0.08	0.887	0.989	0.03	0.826	0.974	-0.05	0.627	0.944	-0.11
PE16_118_0283_1	0.381	0.883	-0.19	0.069	0.969	-0.39	0.538	0.998	-0.14	0.399	0.986	-0.18	0.662	0.974	-0.10	0.335	0.944	-0.21
PE16_118_1253_1	0.680	0.890	0.09	0.815	0.993	0.05	0.570	0.998	0.12	0.639	0.986	0.10	0.672	0.974	0.09	0.486	0.944	0.15
PE18_018_1281_1	0.907	0.947	-0.03	0.557	0.979	-0.13	0.350	0.998	-0.20	0.552	0.986	-0.13	0.262	0.974	-0.24	0.098	0.944	-0.35
PE18_018_2279_1	0.125	0.713	-0.33	0.181	0.969	-0.29	0.232	0.998	-0.26	0.517	0.986	-0.14	0.074	0.974	-0.38	0.026	0.944	-0.46
PE18_020_4303_1	0.985	0.996	0.00	0.924	0.993	-0.02	0.358	0.998	0.20	0.370	0.986	0.20	0.871	0.974	-0.04	0.850	0.944	-0.04
PE18_118_1281_1	0.284	0.772	-0.23	0.605	0.979	-0.11	0.540	0.998	-0.13	0.796	0.986	-0.06	0.481	0.974	-0.15	0.866	0.944	0.04
PE18_118_2279_1	0.201	0.763	-0.28	0.486	0.979	-0.15	0.257	0.998	-0.25	0.472	0.986	-0.16	0.385	0.974	-0.19	0.821	0.944	-0.05
PE18_120_4303_1	0.422	0.890	-0.18	0.577	0.979	-0.12	0.764	0.998	-0.07	0.652	0.986	-0.10	0.491	0.974	-0.15	0.593	0.944	-0.12
PG14_016_0227_1	0.653	0.890	-0.10	0.993	0.993	0.00	0.390	0.998	-0.19	0.724	0.986	-0.08	0.803	0.974	-0.06	0.970	0.991	0.01
PG16_016_0255_1	0.902	0.947	0.03	0.858	0.993	-0.04	0.991	0.998	0.00	0.771	0.986	0.06	0.882	0.974	0.03	0.745	0.944	0.07
PG16_016_1253_1	0.791	0.895	0.06	0.992	0.993	0.00	0.502	0.998	0.15	0.418	0.986	0.18	0.721	0.974	0.08	0.402	0.944	0.18
PG16_018_0255_1	0.744	0.890	0.07	0.913	0.993	0.02	0.865	0.998	0.04	0.663	0.986	0.10	0.712	0.974	0.08	0.934	0.971	0.02
PG16_018_1281_1	0.461	0.890	-0.16	0.856	0.993	-0.04	0.998	0.998	0.00	0.601	0.986	0.12	0.605	0.974	-0.11	0.664	0.944	0.10
PG16_018_2279_1	0.147	0.713	-0.31	0.271	0.979	-0.24	0.762	0.998	-0.07	0.905	0.989	-0.03	0.901	0.984	-0.03	0.778	0.944	0.06
PG16_118_0253_1	0.759	0.890	0.07	0.982	0.993	0.00	0.690	0.998	0.09	0.600	0.986	0.12	0.937	0.989	0.02	0.712	0.944	0.08
PG16_118_1253_1	0.802	0.896	-0.06	0.907	0.993	-0.03	0.450	0.998	0.17	0.420	0.986	0.18	0.729	0.974	0.08	0.176	0.944	0.29
PG18_018_1281_1	0.140	0.713	-0.32	0.304	0.979	-0.22	0.821	0.998	-0.05	0.975	0.989	0.01	0.454	0.974	-0.16	0.760	0.944	-0.07
PG18_018_2279_1	0.048	0.713	-0.42	0.184	0.969	-0.29	0.463	0.998	-0.16	0.478	0.986	-0.16	0.552	0.974	-0.13	0.752	0.944	-0.07
PG18_020_4303_1	0.093	0.713	-0.36	0.071	0.969	-0.38	0.508	0.998	-0.15	0.511	0.986	-0.14	0.223	0.974	-0.26	0.190	0.944	-0.28
PG18_118_1281_1	0.079	0.713	-0.37	0.400	0.979	-0.18	0.915	0.998	-0.02	0.985	0.989	0.00	0.814	0.974	-0.05	0.416	0.944	0.18
PG18_118_2279_1	0.079	0.713	-0.37	0.222	0.979	-0.26	0.667	0.998	-0.09	0.602	0.986	-0.11	0.847	0.974	-0.04	0.703	0.944	0.08
PI16_018_1255_1	0.107	0.713	0.35	0.145	0.969	0.31	0.840	0.998	0.04	0.600	0.986	0.12	0.837	0.974	0.05	0.875	0.944	-0.03
PI18_018_1283_1	0.030	0.713	0.45	0.036	0.969	0.44	0.848	0.998	0.04	0.455	0.986	0.16	0.670	0.974	0.09	0.851	0.944	-0.04
PI18_020_4283_1	0.575	0.890	0.12	0.690	0.993	0.09	0.962	0.998	-0.01	0.910	0.989	0.02	0.967	0.989	0.01	0.616	0.944	-0.11
PI18_118_1281_1	0.158	0.713	0.30	0.076	0.969	0.38	0.877	0.998	0.03	0.534	0.986	0.14	0.655	0.974	0.10	0.639	0.944	0.10
SMd18_112_01	0.762	0.890	-0.07	0.814	0.993	-0.05	0.189	0.998	-0.28	0.280	0.986	-0.23	0.267	0.974	-0.24	0.117	0.944	-0.34
SMd18_114_01	0.232	0.763	0.26	0.481	0.979	0.15	0.184	0.998	0.29	0.257	0.986	0.25	0.240	0.974	0.26	0.283	0.944	0.23

FOLLOW-UP	R5			R5 z-score			R5-20			R5-20 z-score			AX			AX z-score		
	p-value	q-value	R	p-value	q-value	R	p-value	q-value	R	p-value	q-value	R	p-value	q-value	R	p-value	q-value	R
**Lipid class**																		
HexCer	0.856	0.921	0.04	0.486	0.702	0.16	0.515	0.606	-0.15	0.385	0.385	-0.19	0.865	0.928	0.04	0.894	0.939	0.03
LPC	0.921	0.921	0.02	0.875	0.875	0.04	0.509	0.606	-0.15	0.308	0.369	-0.23	0.653	0.928	-0.10	0.443	0.939	-0.17
PE(O)	0.728	0.921	-0.08	0.786	0.875	0.06	0.512	0.606	0.15	0.293	0.369	0.23	0.565	0.928	-0.13	0.788	0.939	0.06
PC	0.836	0.921	-0.05	0.232	0.619	-0.27	0.522	0.606	-0.14	0.208	0.369	-0.28	0.501	0.928	0.15	0.865	0.939	-0.04
PE	0.256	0.921	0.25	0.019	0.154	0.49	0.146	0.606	0.32	0.060	0.369	0.41	0.614	0.928	0.11	0.222	0.939	0.27
PG	0.717	0.921	-0.08	0.527	0.702	0.14	0.286	0.606	0.24	0.161	0.369	0.31	0.856	0.928	-0.04	0.236	0.939	0.26
PI	0.170	0.921	0.30	0.208	0.619	0.28	0.606	0.606	0.12	0.323	0.369	0.22	0.928	0.928	-0.02	0.652	0.939	-0.10
SM	0.641	0.921	0.11	0.386	0.702	0.19	0.535	0.606	-0.14	0.296	0.369	-0.23	0.744	0.928	0.07	0.939	0.939	0.02
**Lipid species**																		
HexCerd18_116_01	0.790	0.949	0.06	0.293	0.810	0.23	0.839	0.907	-0.05	0.646	0.773	-0.10	0.666	0.978	0.10	0.430	0.984	0.18
HexCerd18_118_01	0.773	0.949	0.07	0.276	0.806	0.24	0.930	0.960	0.02	0.855	0.893	-0.04	0.563	0.938	0.13	0.306	0.984	0.23
HexCerd18_120_01	0.737	0.949	0.08	0.277	0.806	0.24	0.921	0.960	0.02	0.874	0.902	-0.04	0.536	0.938	0.14	0.285	0.984	0.24
HexCerd18_122_01	0.776	0.949	-0.06	0.896	0.988	-0.03	0.216	0.670	-0.27	0.254	0.573	-0.25	0.631	0.964	-0.11	0.403	0.984	-0.19
LPC16_01	0.635	0.949	-0.11	0.804	0.962	-0.06	0.416	0.807	-0.18	0.302	0.586	-0.23	0.388	0.863	-0.19	0.360	0.984	-0.20
LPC16_02	0.737	0.949	-0.08	0.787	0.962	-0.06	0.219	0.670	-0.27	0.179	0.544	-0.30	0.557	0.938	-0.13	0.258	0.984	-0.25
LPC16_11	0.624	0.949	-0.11	0.796	0.962	-0.06	0.743	0.889	0.07	0.965	0.971	0.01	0.258	0.863	-0.25	0.906	0.996	-0.03
LPC16_12	0.271	0.773	-0.25	0.346	0.843	-0.21	0.085	0.659	-0.38	0.060	0.464	-0.41	0.297	0.863	-0.23	0.200	0.984	-0.28
LPC18_01	0.539	0.949	-0.14	0.610	0.948	-0.12	0.164	0.662	-0.31	0.138	0.507	-0.33	0.444	0.863	-0.17	0.214	0.984	-0.28
LPC18_02	0.483	0.949	-0.16	0.564	0.948	-0.13	0.156	0.662	-0.31	0.129	0.507	-0.33	0.384	0.863	-0.20	0.195	0.984	-0.29
LPC18_11	0.244	0.773	-0.26	0.260	0.806	-0.25	0.097	0.659	-0.36	0.032	0.431	-0.46	0.262	0.863	-0.25	0.212	0.984	-0.28
LPC18_12	0.649	0.949	-0.10	0.768	0.962	-0.07	0.195	0.662	-0.29	0.144	0.507	-0.32	0.553	0.938	-0.13	0.295	0.984	-0.23
LPC18_21	0.492	0.949	0.15	0.983	0.988	0.00	0.625	0.878	-0.11	0.332	0.594	-0.22	0.623	0.964	0.11	0.918	0.996	-0.02
LPC18_22	0.009	0.283	0.54	0.063	0.699	0.40	0.715	0.882	0.08	0.799	0.858	0.06	0.097	0.671	0.36	0.573	0.984	0.13
PE O-2:0a/18:1	0.593	0.949	-0.12	0.914	0.988	-0.02	0.561	0.859	0.13	0.458	0.725	0.17	0.431	0.863	-0.18	0.872	0.996	0.04
PE O-2:0a/18:2	0.664	0.949	-0.10	0.959	0.988	0.01	0.709	0.882	0.08	0.279	0.586	0.24	0.395	0.863	-0.19	0.933	0.996	0.02
PE O-2:0a/20:1	0.710	0.949	-0.08	0.944	0.988	-0.02	0.458	0.822	0.17	0.317	0.591	0.22	0.948	0.997	0.01	0.766	0.996	0.07
PE O-2:0a/20:4	0.984	0.996	0.00	0.605	0.948	0.12	0.261	0.677	0.25	0.164	0.528	0.31	0.697	0.978	0.09	0.657	0.996	0.10
PC14_014_0	0.845	0.949	0.04	0.655	0.948	-0.10	0.199	0.662	-0.28	0.167	0.528	-0.31	0.333	0.863	0.22	0.817	0.996	0.05
PC14_016_0	0.244	0.773	0.26	0.673	0.948	0.10	0.518	0.833	-0.15	0.523	0.773	-0.14	0.119	0.709	0.34	0.554	0.984	0.13
PC14_016_1	0.800	0.949	0.06	0.661	0.948	-0.10	0.611	0.878	-0.11	0.631	0.773	-0.11	0.306	0.863	0.23	0.424	0.984	0.18
PC14_018_0	0.435	0.949	-0.18	0.641	0.948	-0.11	0.066	0.659	-0.40	0.048	0.464	-0.43	0.864	0.980	0.04	0.806	0.996	0.06
PC14_018_1	0.262	0.773	-0.25	0.350	0.843	-0.21	0.149	0.662	-0.32	0.112	0.507	-0.35	0.798	0.978	-0.06	0.987	0.996	0.00
PC14_018_2	0.325	0.857	-0.22	0.662	0.948	-0.10	0.304	0.677	-0.23	0.404	0.685	-0.19	0.727	0.978	-0.08	0.894	0.996	-0.03
PC14_116_0	0.242	0.773	0.26	0.278	0.806	0.24	0.619	0.878	0.11	0.651	0.773	0.10	0.066	0.622	0.40	0.087	0.984	0.37
PC15_016_0	0.843	0.949	-0.04	0.545	0.948	-0.14	0.455	0.822	-0.17	0.627	0.773	-0.11	0.821	0.978	0.05	0.720	0.996	-0.08
PC15_018_1	0.678	0.949	-0.09	0.969	0.988	0.01	0.087	0.659	-0.37	0.267	0.576	-0.25	0.284	0.863	-0.24	0.858	0.996	-0.04
PC16.0_80al	0.051	0.773	-0.42	0.074	0.699	-0.39	0.033	0.568	-0.46	0.038	0.452	-0.44	0.332	0.863	-0.22	0.459	0.984	-0.17
PC160_70al	0.732	0.949	-0.08	0.135	0.806	-0.33	0.296	0.677	-0.23	0.209	0.549	-0.28	0.594	0.964	-0.12	0.429	0.984	-0.18
PC160_90al	0.462	0.949	-0.17	0.069	0.699	-0.39	0.166	0.662	-0.31	0.062	0.464	-0.40	0.761	0.978	-0.07	0.405	0.984	-0.19
PC16_016_0	0.677	0.949	0.09	0.383	0.867	-0.20	0.913	0.960	0.02	0.649	0.773	-0.10	0.413	0.863	0.18	0.767	0.996	-0.07
PC16_016_1	0.094	0.773	0.37	0.229	0.806	0.27	0.023	0.542	0.48	0.013	0.310	0.52	0.278	0.863	0.24	0.187	0.984	0.29
PC16_017_0	0.645	0.949	-0.10	0.791	0.962	-0.06	0.816	0.907	-0.05	0.948	0.969	0.01	0.997	0.997	0.00	0.996	0.996	0.00
PC16_017_1	0.226	0.773	0.27	0.066	0.699	0.40	0.007	0.230	0.56	0.013	0.310	0.52	0.106	0.671	0.35	0.027	0.866	0.47
PC16_017_11	0.843	0.949	-0.04	0.810	0.962	-0.05	0.689	0.882	0.09	0.494	0.757	0.15	0.428	0.863	0.18	0.684	0.996	0.09
PC16_018_0	0.235	0.773	-0.26	0.655	0.948	-0.10	0.459	0.822	-0.17	0.235	0.567	-0.26	0.704	0.978	-0.09	0.820	0.996	0.05
PC16_018_1	0.141	0.773	-0.32	0.808	0.962	-0.05	0.675	0.882	-0.09	0.622	0.773	-0.11	0.085	0.671	-0.38	0.694	0.996	-0.09
PC16_018_2	0.572	0.949	-0.13	0.669	0.948	0.10	0.977	0.988	-0.01	0.786	0.858	0.06	0.415	0.863	-0.18	0.854	0.996	-0.04
PC16_018_3	0.996	0.996	0.00	0.549	0.948	0.13	0.287	0.677	0.24	0.098	0.507	0.36	0.798	0.978	-0.06	0.916	0.996	-0.02
PC16_018_31	0.522	0.949	0.14	0.145	0.806	0.32	0.510	0.833	0.15	0.699	0.810	0.09	0.467	0.870	0.16	0.333	0.984	0.22
PC16_020_4	0.910	0.971	0.03	0.364	0.843	0.20	0.266	0.677	0.25	0.452	0.725	0.17	0.368	0.863	0.20	0.472	0.984	0.16
PC16_020_5	0.810	0.949	-0.05	0.551	0.948	0.13	0.814	0.907	0.05	0.571	0.773	0.13	0.801	0.978	0.06	0.849	0.996	0.04
PC16_022_6	0.530	0.949	0.14	0.221	0.806	0.27	0.168	0.662	0.30	0.086	0.507	0.37	0.195	0.863	0.29	0.353	0.984	0.21
PC16_0e16_0	0.095	0.773	0.37	0.227	0.806	0.27	0.387	0.790	0.19	0.526	0.773	0.14	0.287	0.863	0.24	0.382	0.984	0.20
PC16_0p16_0	0.924	0.975	0.02	0.343	0.843	0.21	0.840	0.907	-0.05	0.568	0.773	0.13	0.986	0.997	0.00	0.965	0.996	0.01
PC16_116_1	0.218	0.773	0.27	0.604	0.948	0.12	0.277	0.677	0.24	0.194	0.544	0.29	0.171	0.855	0.30	0.232	0.984	0.27
PC16_118_0	0.818	0.949	-0.05	0.507	0.948	0.15	0.592	0.878	0.12	0.619	0.773	0.11	0.960	0.997	-0.01	0.190	0.984	0.29
PC16_118_1	0.869	0.949	-0.04	0.830	0.974	0.05	0.555	0.859	0.13	0.642	0.773	0.10	0.800	0.978	0.06	0.355	0.984	0.21
PC16_118_2	0.267	0.773	-0.25	0.361	0.843	-0.20	0.526	0.833	-0.14	0.674	0.791	-0.09	0.557	0.938	-0.13	0.990	0.996	0.00
PC180_90al	0.243	0.773	-0.26	0.055	0.699	-0.41	0.075	0.659	-0.39	0.016	0.310	-0.51	0.369	0.863	-0.20	0.290	0.984	-0.24
PC18_018_1	0.069	0.773	-0.39	0.854	0.978	-0.04	0.650	0.882	-0.10	0.610	0.773	-0.12	0.039	0.534	-0.44	0.550	0.984	-0.13
PC18_018_2	0.243	0.773	-0.26	0.810	0.962	0.05	0.604	0.878	-0.12	0.815	0.860	-0.05	0.105	0.671	-0.35	0.571	0.984	-0.13
PC18_020_4	0.821	0.949	-0.05	0.446	0.948	0.17	0.430	0.817	0.18	0.568	0.773	0.13	0.775	0.978	0.06	0.811	0.996	0.05
PC18_118_1	0.236	0.773	-0.26	0.718	0.962	-0.08	0.709	0.882	-0.08	0.769	0.853	-0.07	0.329	0.863	-0.22	0.962	0.996	-0.01
PC18_118_2	0.209	0.773	-0.28	0.653	0.948	-0.10	0.509	0.833	-0.15	0.710	0.813	-0.08	0.329	0.863	-0.22	0.676	0.996	-0.09
PC18_120_4	0.714	0.949	-0.08	0.643	0.948	0.10	0.900	0.960	-0.03	0.613	0.773	-0.11	0.639	0.964	0.11	0.505	0.984	0.15
PC18_218_2	0.768	0.949	-0.07	0.455	0.948	0.17	0.834	0.907	-0.05	0.804	0.858	0.06	0.897	0.980	-0.03	0.843	0.996	0.04
PE14_018_1281_1	0.054	0.773	0.42	0.666	0.948	0.10	0.677	0.882	-0.09	0.540	0.773	-0.14	0.036	0.534	0.45	0.213	0.984	0.28
PE16_016_1253_1	0.002	0.150	0.63	0.110	0.748	0.35	0.004	0.184	0.59	0.010	0.310	0.54	0.004	0.389	0.59	0.089	0.984	0.37
PE16_018_1281_1	0.110	0.773	0.35	0.070	0.699	0.39	0.318	0.677	0.22	0.199	0.544	0.28	0.395	0.863	0.19	0.588	0.984	0.12
PE16_018_2279_1	0.369	0.921	0.20	0.104	0.748	0.36	0.644	0.882	0.10	0.308	0.586	0.23	0.824	0.978	0.05	0.570	0.984	0.13
PE16_020_4303_1	0.119	0.773	0.34	0.069	0.699	0.39	0.079	0.659	0.38	0.142	0.507	0.32	0.035	0.534	0.45	0.194	0.984	0.29
PE16_118_0283_1	0.478	0.949	0.16	0.167	0.806	0.31	0.321	0.677	0.22	0.298	0.586	0.23	0.149	0.787	0.32	0.040	0.952	0.44
PE16_118_1253_1	0.007	0.283	0.56	0.008	0.699	0.55	0.094	0.659	0.37	0.063	0.464	0.40	0.012	0.389	0.52	0.012	0.564	0.53
PE18_018_1281_1	0.627	0.949	0.11	0.143	0.806	0.32	0.143	0.662	0.32	0.093	0.507	0.37	0.877	0.980	-0.04	0.484	0.984	0.16
PE18_018_2279_1	0.955	0.987	0.01	0.231	0.806	0.27	0.739	0.889	0.08	0.338	0.595	0.21	0.442	0.863	-0.17	0.980	0.996	-0.01
PE18_020_4303_1	0.204	0.773	0.28	0.058	0.699	0.41	0.123	0.662	0.34	0.126	0.507	0.34	0.205	0.863	0.28	0.293	0.984	0.23
PE18_118_1281_1	0.583	0.949	0.12	0.108	0.748	0.35	0.311	0.677	0.23	0.187	0.544	0.29	0.985	0.997	0.00	0.330	0.984	0.22
PE18_118_2279_1	0.881	0.951	0.03	0.270	0.806	0.25	0.758	0.889	0.07	0.430	0.717	0.18	0.880	0.980	-0.03	0.600	0.984	0.12
PE18_120_4303_1	0.380	0.927	0.20	0.176	0.806	0.30	0.202	0.662	0.28	0.287	0.586	0.24	0.072	0.622	0.39	0.145	0.984	0.32
PG14_016_0227_1	0.232	0.773	0.27	0.775	0.962	0.06	0.501	0.833	0.15	0.381	0.658	0.20	0.051	0.606	0.42	0.272	0.984	0.25
PG16_016_0255_1	0.257	0.773	0.25	0.988	0.988	0.00	0.170	0.662	0.30	0.127	0.507	0.34	0.063	0.622	0.40	0.124	0.984	0.34
PG16_016_1253_1	0.075	0.773	0.39	0.442	0.948	0.17	0.036	0.568	0.45	0.026	0.410	0.47	0.016	0.389	0.51	0.086	0.984	0.37
PG16_018_0255_1	0.628	0.949	0.11	0.854	0.978	0.04	0.200	0.662	0.28	0.152	0.515	0.32	0.214	0.863	0.28	0.105	0.984	0.35
PG16_018_1281_1	0.769	0.949	0.07	0.249	0.806	0.26	0.151	0.662	0.32	0.088	0.507	0.37	0.600	0.964	0.12	0.089	0.984	0.37
PG16_018_2279_1	0.989	0.996	0.00	0.915	0.988	0.02	0.752	0.889	0.07	0.606	0.773	0.12	0.821	0.978	0.05	0.912	0.996	0.02
PG16_118_0253_1	0.038	0.773	0.45	0.084	0.722	0.38	0.003	0.184	0.60	0.001	0.065	0.67	0.009	0.389	0.54	0.006	0.558	0.57
PG16_118_1253_1	0.240	0.773	0.26	0.280	0.806	0.24	0.130	0.662	0.33	0.057	0.464	0.41	0.231	0.863	0.27	0.115	0.984	0.35
PG18_018_1281_1	0.367	0.921	-0.20	0.543	0.948	0.14	0.416	0.807	0.18	0.308	0.586	0.23	0.445	0.863	-0.17	0.417	0.984	0.18
PG18_018_2279_1	0.613	0.949	-0.11	0.926	0.988	0.02	0.996	0.996	0.00	0.772	0.853	0.07	0.720	0.978	-0.08	0.995	0.996	0.00
PG18_020_4303_1	0.625	0.949	0.11	0.202	0.806	0.28	0.064	0.659	0.40	0.078	0.507	0.38	0.678	0.978	0.09	0.289	0.984	0.24
PG18_118_1281_1	0.312	0.846	-0.23	0.879	0.988	0.03	0.940	0.960	0.02	0.736	0.833	0.08	0.219	0.863	-0.27	0.876	0.996	0.04
PG18_118_2279_1	0.499	0.949	-0.15	0.911	0.988	-0.03	0.698	0.882	-0.09	0.971	0.971	-0.01	0.461	0.870	-0.17	0.752	0.996	-0.07
PI16_018_1255_1	0.076	0.773	0.39	0.175	0.806	0.30	0.796	0.907	0.06	0.569	0.773	0.13	0.522	0.938	0.14	0.852	0.996	-0.04
PI18_018_1283_1	0.183	0.773	0.29	0.242	0.806	0.26	0.495	0.833	0.15	0.259	0.573	0.25	0.994	0.997	0.00	0.579	0.984	-0.13
PI18_020_4283_1	0.139	0.773	0.33	0.067	0.699	0.40	0.189	0.662	0.29	0.104	0.507	0.36	0.127	0.712	0.34	0.533	0.984	0.14
PI18_118_1281_1	0.277	0.773	0.24	0.322	0.843	0.22	0.790	0.907	0.06	0.453	0.725	0.17	0.685	0.978	-0.09	0.601	0.984	-0.12
SMd18_112_01	0.420	0.949	0.18	0.299	0.810	0.23	0.628	0.878	-0.11	0.469	0.731	-0.16	0.359	0.863	0.21	0.555	0.984	0.13
SMd18_114_01	0.837	0.949	0.05	0.633	0.948	0.11	0.311	0.677	-0.23	0.214	0.549	-0.28	0.873	0.980	0.04	0.791	0.996	-0.06

SMd18_114_11	0.562	0.890	0.13	0.589	0.979	0.12	0.557	0.998	0.13	0.456	0.986	0.16	0.396	0.974	0.19	0.303	0.944	0.22
SMd18_116_01	0.151	0.713	0.31	0.150	0.969	0.31	0.403	0.998	0.18	0.356	0.986	0.20	0.697	0.974	0.09	0.582	0.944	0.12
SMd18_116_11	0.497	0.890	0.15	0.335	0.979	0.21	0.192	0.998	0.28	0.177	0.986	0.29	0.657	0.974	0.10	0.181	0.944	0.29
SMd18_117_01	0.233	0.763	0.26	0.413	0.979	0.18	0.290	0.998	0.23	0.214	0.986	0.27	0.369	0.974	0.20	0.696	0.944	0.09
SMd18_118_01	0.777	0.890	0.06	0.601	0.979	0.11	0.430	0.998	0.17	0.328	0.986	0.21	0.659	0.974	0.10	0.499	0.944	0.15
SMd18_118_11	0.635	0.890	0.10	0.385	0.979	0.19	0.330	0.998	0.21	0.208	0.986	0.27	0.659	0.974	0.10	0.281	0.944	0.23
**Proteins**																		
Alpha-2-HS-glycoprotein	0.528	0.736	0.14	0.997	0.997	0.00	0.691	0.883	-0.09	0.305	0.609	-0.23	0.611	0.770	0.11	0.867	0.970	-0.04
Angiotensinogen	0.557	0.754	0.13	0.963	0.997	-0.01	0.541	0.777	0.14	0.540	0.815	0.14	0.991	0.991	0.00	0.733	0.963	-0.08
Antithrombin-III	0.128	0.336	0.33	0.472	0.805	0.16	0.026	0.135	0.47	0.052	0.216	0.42	0.288	0.675	0.24	0.677	0.933	0.09
Beta-1,4-galactosyltransferase 1	0.279	0.475	-0.24	0.273	0.805	-0.24	0.594	0.828	-0.12	0.871	0.932	-0.04	0.529	0.747	-0.14	0.626	0.933	-0.11
Beta-2-glycoprotein 1	0.054	0.256	0.42	0.180	0.805	0.30	0.011	0.071	0.53	0.037	0.205	0.45	0.161	0.675	0.31	0.555	0.933	0.13
Biotinidase	0.800	0.920	-0.06	0.648	0.877	-0.10	0.938	0.959	-0.02	0.928	0.947	-0.02	0.517	0.747	-0.15	0.480	0.933	-0.16
C-C motif chemokine 18	0.185	0.405	0.29	0.568	0.855	0.13	0.229	0.465	0.27	0.318	0.609	0.22	0.387	0.743	0.19	0.907	0.970	0.03
Cartilage acidic protein 1	0.862	0.921	0.04	0.464	0.805	-0.16	0.799	0.894	-0.06	0.947	0.947	-0.01	0.946	0.991	0.02	0.597	0.933	-0.12
Complement C3b, inactivated	0.940	0.940	-0.02	0.402	0.805	-0.19	0.373	0.660	0.20	0.368	0.651	0.20	0.906	0.991	0.03	0.571	0.933	-0.13
Complement C4b	0.036	0.256	0.45	0.442	0.805	0.17	0.068	0.244	0.40	0.036	0.205	0.45	0.114	0.675	0.35	0.488	0.933	0.16
Complement C5	0.887	0.921	0.03	0.613	0.855	-0.11	0.069	0.244	0.39	0.139	0.425	0.33	0.460	0.743	0.17	0.540	0.933	0.14
Complement decay-accelerating factor	0.730	0.861	-0.08	0.584	0.855	-0.12	0.472	0.724	-0.16	0.790	0.897	-0.06	0.685	0.808	-0.09	0.399	0.933	-0.19
Complement factor B	0.018	0.256	0.50	0.140	0.805	0.32	0.008	0.065	0.55	0.014	0.133	0.51	0.142	0.675	0.32	0.313	0.933	0.23
Complement factor D	0.250	0.475	0.26	0.496	0.815	0.15	0.119	0.366	0.34	0.252	0.526	0.26	0.027	0.675	0.47	0.255	0.933	0.25
Complement factor I	0.060	0.256	0.41	0.266	0.805	0.25	0.000	0.022	0.68	0.002	0.076	0.63	0.054	0.675	0.42	0.148	0.933	0.32
Contactin-6	0.149	0.360	0.32	0.955	0.997	-0.01	0.417	0.699	0.18	0.440	0.723	0.17	0.285	0.675	0.24	0.993	0.993	0.00
EGF-like repeat and discoidin I-like domain-containing protein 3	0.255	0.475	-0.25	0.219	0.805	-0.27	0.202	0.443	-0.28	0.353	0.650	-0.21	0.331	0.699	-0.22	0.689	0.933	-0.09
Ephrin-A1	0.659	0.838	-0.10	0.415	0.805	-0.18	0.340	0.625	-0.21	0.537	0.815	-0.14	0.445	0.743	-0.17	0.362	0.933	-0.20
Extracellular superoxide dismutase [Cu-Zn]	0.847	0.921	-0.04	0.982	0.997	-0.01	0.972	0.972	-0.01	0.892	0.933	-0.03	0.595	0.770	-0.12	0.803	0.970	-0.06
Fatty acid-binding protein, heart	0.475	0.683	0.16	0.934	0.997	-0.02	0.540	0.777	0.14	0.609	0.824	0.12	0.104	0.675	0.36	0.956	0.993	0.01
Fructose-1,6-bisphosphatase 1	0.279	0.475	0.24	0.109	0.805	0.35	0.848	0.907	0.04	0.549	0.815	0.14	0.402	0.743	0.19	0.591	0.933	0.12
Gelsolin	0.421	0.624	0.18	0.430	0.805	0.18	0.131	0.377	0.33	0.216	0.493	0.27	0.502	0.747	0.15	0.588	0.933	0.12
Haptoglobin isoform 2	0.295	0.475	0.23	0.201	0.805	0.28	0.052	0.216	0.42	0.091	0.322	0.37	0.279	0.675	0.24	0.061	0.933	0.41
Hemopexin	0.061	0.256	0.41	0.187	0.805	0.29	0.005	0.055	0.58	0.009	0.105	0.54	0.076	0.675	0.39	0.195	0.933	0.29
Inter-alpha-trypsin inhibitor heavy chain H2	0.072	0.256	0.39	0.347	0.805	0.21	0.032	0.149	0.46	0.062	0.239	0.40	0.465	0.743	0.16	0.971	0.993	0.01
Interleukin-6 receptor subunit beta	0.725	0.861	-0.08	0.298	0.805	-0.23	0.174	0.402	-0.30	0.225	0.493	-0.27	0.334	0.699	-0.22	0.168	0.933	-0.30
Low affinity immunoglobulin gamma Fc region receptor III-B	0.283	0.475	0.24	0.872	0.997	-0.04	0.645	0.848	0.10	0.593	0.824	0.12	0.989	0.991	0.00	0.241	0.933	-0.26
Lumican	0.299	0.475	0.23	0.214	0.805	0.28	0.150	0.402	0.32	0.201	0.486	0.28	0.547	0.747	0.14	0.598	0.933	0.12
Macrophage colony-stimulating factor 1 receptor	0.674	0.838	0.10	0.766	0.995	-0.07	0.778	0.894	-0.06	0.631	0.829	-0.11	0.552	0.747	-0.13	0.307	0.933	-0.23
Macrophage mannose receptor 1	0.377	0.578	0.20	0.991	0.997	0.00	0.816	0.894	0.05	0.688	0.842	0.09	0.192	0.675	0.29	0.854	0.970	0.04
Neuroligin-1	0.096	0.294	0.36	0.307	0.805	0.23	0.425	0.699	0.18	0.743	0.876	0.07	0.261	0.675	0.25	0.231	0.933	0.27
Neurotrimin	0.021	0.256	0.49	0.124	0.805	0.34	0.169	0.402	0.30	0.042	0.205	0.44	0.211	0.675	0.28	0.600	0.933	0.12
Noggin	0.059	0.256	0.41	0.327	0.805	0.22	0.778	0.894	0.06	0.420	0.715	0.18	0.987	0.991	0.00	0.884	0.970	0.03
Plasma protease C1 inhibitor	0.173	0.398	0.30	0.897	0.997	0.03	0.175	0.402	0.30	0.145	0.425	0.32	0.412	0.743	0.18	0.684	0.933	-0.09
Plexin-D1	0.615	0.808	0.11	0.398	0.805	-0.19	0.905	0.946	-0.03	0.800	0.897	-0.06	0.747	0.859	0.07	0.522	0.933	-0.14
Prothrombin	0.079	0.260	0.38	0.287	0.805	0.24	0.008	0.065	0.55	0.026	0.196	0.47	0.196	0.675	0.29	0.383	0.933	0.20
Pulmonary surfactant-associated protein D	0.007	0.256	0.56	0.064	0.805	0.40	0.279	0.535	0.24	0.195	0.486	0.29	0.468	0.743	0.16	0.891	0.970	0.03
Receptor-type tyrosine-protein phosphatase S	0.871	0.921	0.04	0.884	0.997	0.03	0.456	0.723	0.17	0.659	0.842	0.10	0.293	0.675	0.23	0.257	0.933	0.25
Retinol-binding protein 4	0.901	0.921	0.03	0.778	0.995	0.06	0.723	0.894	0.08	0.695	0.842	0.09	0.883	0.990	-0.03	0.491	0.933	-0.16
Serotransferrin	0.132	0.336	0.33	0.905	0.997	0.03	0.003	0.055	0.61	0.008	0.105	0.55	0.048	0.675	0.43	0.305	0.933	0.23
Serum albumin	0.072	0.256	0.39	0.231	0.805	0.27	0.106	0.349	0.35	0.148	0.425	0.32	0.276	0.675	0.24	0.849	0.970	0.04
Thyroxine-binding globulin	0.034	0.256	0.45	0.294	0.805	0.23	0.005	0.055	0.58	0.006	0.105	0.57	0.158	0.675	0.31	0.438	0.933	0.17
Transmembrane protein 2	0.221	0.462	0.27	0.530	0.840	0.14	0.756	0.894	-0.07	0.591	0.824	-0.12	0.672	0.808	0.10	0.664	0.933	0.10
Triggering receptor expressed on myeloid cells 2	0.040	0.256	0.44	0.376	0.805	0.20	0.625	0.846	0.11	0.865	0.932	0.04	0.619	0.770	0.11	0.825	0.970	-0.05
V-set and immunoglobulin domain-containing protein 4	0.122	0.336	0.34	0.607	0.855	0.12	0.233	0.465	0.27	0.179	0.483	0.30	0.192	0.675	0.29	0.681	0.933	0.09
Vitamin D-binding protein	0.063	0.256	0.40	0.312	0.805	0.23	0.018	0.103	0.50	0.044	0.205	0.43	0.194	0.675	0.29	0.336	0.933	0.22

SMd18_114_11	0.869	0.949	-0.04	0.697	0.959	0.09	0.251	0.677	-0.26	0.245	0.568	-0.26	0.755	0.978	-0.07	0.719	0.996	-0.08
SMd18_116_01	0.790	0.949	0.06	0.533	0.948	0.14	0.391	0.790	-0.19	0.239	0.567	-0.26	0.938	0.997	0.02	0.797	0.996	-0.06
SMd18_116_11	0.839	0.949	0.05	0.679	0.948	0.09	0.298	0.677	-0.23	0.223	0.556	-0.27	0.930	0.997	-0.02	0.581	0.984	-0.12
SMd18_117_01	0.644	0.949	-0.10	0.947	0.988	0.02	0.226	0.670	-0.27	0.136	0.507	-0.33	0.633	0.964	-0.11	0.559	0.984	-0.13
SMd18_118_01	0.956	0.987	-0.01	0.516	0.948	0.15	0.500	0.833	-0.15	0.326	0.594	-0.22	0.896	0.980	0.03	0.735	0.996	0.08
SMd18_118_11	0.857	0.949	-0.04	0.720	0.962	0.08	0.286	0.677	-0.24	0.201	0.544	-0.28	0.855	0.980	-0.04	0.785	0.996	-0.06
**Proteins**																		
Alpha-2-HS-glycoprotein	0.842	0.993	0.05	0.994	0.999	0.00	0.225	0.370	-0.31	0.133	0.346	-0.38	0.780	0.980	0.07	0.936	0.998	-0.02
Angiotensinogen	0.593	0.968	-0.14	0.898	0.999	0.03	0.121	0.319	0.39	0.153	0.346	0.36	0.885	0.980	0.04	0.585	0.998	0.14
Antithrombin-III	0.461	0.878	-0.19	0.996	0.999	0.00	0.039	0.261	0.50	0.164	0.346	0.35	0.973	0.980	-0.01	0.825	0.998	0.06
Beta-1,4-galactosyltransferase 1	0.416	0.878	-0.21	0.773	0.999	0.08	0.174	0.333	0.35	0.090	0.346	0.42	0.385	0.980	-0.23	0.906	0.998	-0.03
Beta-2-glycoprotein 1	0.448	0.878	-0.20	0.876	0.999	-0.04	0.027	0.261	0.54	0.172	0.346	0.35	0.794	0.980	0.07	0.786	0.998	0.07
Biotinidase	0.339	0.878	-0.25	0.842	0.999	0.05	0.084	0.295	0.43	0.073	0.346	0.45	0.528	0.980	-0.16	0.998	0.998	0.00
C-C motif chemokine 18	0.993	0.993	0.00	0.581	0.999	-0.14	0.497	0.586	0.18	0.785	0.840	0.07	0.871	0.980	0.04	0.978	0.998	-0.01
Cartilage acidic protein 1	0.772	0.993	0.08	0.859	0.999	-0.05	0.225	0.370	0.31	0.153	0.346	0.36	0.741	0.980	-0.09	0.806	0.998	-0.06
Complement C3b, inactivated	0.725	0.993	-0.09	0.630	0.999	0.13	0.048	0.261	0.49	0.072	0.346	0.45	0.827	0.980	-0.06	0.690	0.998	0.10
Complement C4b	0.461	0.878	-0.19	0.786	0.999	0.07	0.146	0.319	0.37	0.156	0.346	0.36	0.474	0.980	-0.19	0.987	0.998	0.00
Complement C5	0.467	0.878	-0.19	0.680	0.999	0.11	0.100	0.301	0.41	0.131	0.346	0.38	0.693	0.980	-0.10	0.837	0.998	0.05
Complement decay-accelerating factor	0.453	0.878	0.19	0.201	0.999	0.33	0.073	0.281	0.45	0.014	0.346	0.58	0.888	0.980	-0.04	0.520	0.998	0.17
Complement factor B	0.451	0.878	-0.20	0.746	0.999	0.09	0.067	0.280	0.45	0.173	0.346	0.35	0.945	0.980	-0.02	0.644	0.998	0.12
Complement factor D	0.463	0.878	-0.19	0.664	0.999	0.11	0.276	0.424	0.28	0.359	0.501	0.24	0.471	0.980	-0.19	0.949	0.998	0.02
Complement factor I	0.301	0.878	-0.27	0.996	0.999	0.00	0.090	0.295	0.42	0.159	0.346	0.36	0.641	0.980	-0.12	0.985	0.998	0.00
Contactin-6	0.858	0.993	0.05	0.521	0.999	-0.17	0.616	0.708	0.13	0.376	0.502	0.23	0.778	0.980	-0.07	0.350	0.998	-0.24
EGF-like repeat and discoidin I-like domain-containing protein 3	0.976	0.993	-0.01	0.929	0.999	0.02	0.349	0.487	0.24	0.094	0.346	0.42	0.237	0.980	-0.30	0.588	0.998	-0.14
Ephrin-A1	0.460	0.878	-0.19	0.714	0.999	-0.10	0.477	0.577	0.19	0.234	0.394	0.31	0.202	0.980	-0.33	0.494	0.998	-0.18
Extracellular superoxide dismutase [Cu-Zn]	0.210	0.878	-0.32	0.943	0.999	-0.02	0.402	0.519	0.22	0.298	0.428	0.27	0.371	0.980	-0.23	0.796	0.998	-0.07
Fatty acid-binding protein, heart	0.869	0.993	-0.04	0.679	0.999	-0.11	0.638	0.716	0.12	0.484	0.544	0.18	0.840	0.980	-0.05	0.877	0.998	-0.04
Fructose-1,6-bisphosphatase 1	0.309	0.878	-0.26	0.752	0.999	0.08	0.162	0.324	0.36	0.076	0.346	0.44	0.396	0.980	-0.22	0.995	0.998	0.00
Gelsolin	0.653	0.968	-0.12	0.665	0.999	-0.11	0.105	0.301	0.41	0.475	0.544	0.19	0.602	0.980	0.14	0.801	0.998	0.07
Haptoglobin isoform 2	0.508	0.878	-0.17	0.630	0.999	0.13	0.057	0.261	0.47	0.042	0.346	0.50	0.408	0.980	-0.21	0.998	0.998	0.00
Hemopexin	0.484	0.878	-0.18	0.867	0.999	0.04	0.055	0.261	0.47	0.153	0.346	0.36	0.913	0.980	0.03	0.633	0.998	0.12
Inter-alpha-trypsin inhibitor heavy chain H2	0.336	0.878	-0.25	0.771	0.999	0.08	0.145	0.319	0.37	0.207	0.366	0.32	0.655	0.980	-0.12	0.914	0.998	0.03
Interleukin-6 receptor subunit beta	0.363	0.878	0.24	0.790	0.999	0.07	0.153	0.320	0.36	0.063	0.346	0.46	0.949	0.980	0.02	0.804	0.998	0.07
Low affinity immunoglobulin gamma Fc region receptor III-B	0.515	0.878	-0.17	0.699	0.999	0.10	0.021	0.261	0.56	0.036	0.346	0.51	0.980	0.980	-0.01	0.482	0.998	0.18
Lumican	0.985	0.993	0.00	0.574	0.999	0.15	0.044	0.261	0.49	0.272	0.422	0.28	0.495	0.980	0.18	0.356	0.998	0.24
Macrophage colony-stimulating factor 1 receptor	0.231	0.878	-0.31	0.751	0.999	-0.08	0.207	0.370	0.32	0.275	0.422	0.28	0.958	0.980	-0.01	0.890	0.998	0.04
Macrophage mannose receptor 1	0.927	0.993	0.02	0.884	0.999	-0.04	0.976	0.976	-0.01	0.406	0.519	0.22	0.591	0.980	-0.14	0.459	0.998	-0.19
Neuroligin-1	0.036	0.878	-0.51	0.058	0.999	-0.47	0.417	0.519	-0.21	0.485	0.544	-0.18	0.471	0.980	-0.19	0.287	0.998	-0.27
Neurotrimin	0.651	0.968	-0.12	0.395	0.999	-0.22	0.225	0.370	-0.31	0.183	0.350	-0.34	0.327	0.980	-0.25	0.657	0.998	-0.12
Noggin	0.472	0.878	-0.19	0.826	0.999	-0.06	0.698	0.756	-0.10	0.753	0.825	0.08	0.411	0.980	-0.21	0.429	0.998	-0.21
Plasma protease C1 inhibitor	0.738	0.993	-0.09	0.462	0.999	0.19	0.042	0.261	0.50	0.067	0.346	0.45	0.822	0.980	-0.06	0.581	0.998	0.14
Plexin-D1	0.744	0.993	-0.09	0.264	0.999	-0.29	0.753	0.787	0.08	0.382	0.502	0.23	0.382	0.980	-0.23	0.163	0.998	-0.35
Prothrombin	0.462	0.878	-0.19	0.935	0.999	-0.02	0.056	0.261	0.47	0.297	0.428	0.27	0.888	0.980	0.04	0.803	0.998	0.07
Pulmonary surfactant-associated protein D	0.991	0.993	0.00	0.693	0.999	-0.10	0.243	0.386	0.30	0.159	0.346	0.36	0.980	0.980	0.01	0.512	0.998	-0.17
Receptor-type tyrosine-protein phosphatase S	0.137	0.878	-0.38	0.999	0.999	0.00	0.291	0.432	0.27	0.196	0.360	0.33	0.334	0.980	-0.25	0.828	0.998	-0.06
Retinol-binding protein 4	0.652	0.968	-0.12	0.395	0.999	0.22	0.129	0.319	0.38	0.159	0.346	0.36	0.953	0.980	-0.02	0.471	0.998	0.19
Serotransferrin	0.451	0.878	-0.20	0.646	0.999	-0.12	0.026	0.261	0.54	0.086	0.346	0.43	0.801	0.980	0.07	0.886	0.998	0.04
Serum albumin	0.450	0.878	0.20	0.993	0.999	0.00	0.707	0.756	0.10	0.904	0.945	-0.03	0.241	0.980	0.30	0.482	0.998	0.18
Thyroxine-binding globulin	0.407	0.878	-0.22	0.922	0.999	-0.03	0.143	0.319	0.37	0.240	0.394	0.30	0.642	0.980	-0.12	0.982	0.998	0.01
Transmembrane protein 2	0.962	0.993	-0.01	0.285	0.999	-0.28	0.302	0.434	-0.27	0.457	0.544	-0.19	0.925	0.980	0.02	0.505	0.998	-0.17
Triggering receptor expressed on myeloid cells 2	0.094	0.878	-0.42	0.327	0.999	-0.25	0.414	0.519	0.21	0.960	0.981	-0.01	0.669	0.980	0.11	0.907	0.998	0.03
V-set and immunoglobulin domain-containing protein 4	0.809	0.993	0.06	0.590	0.999	-0.14	0.856	0.875	0.05	0.482	0.544	0.18	0.683	0.980	-0.11	0.471	0.998	-0.19
Vitamin D-binding protein	0.952	0.993	0.02	0.247	0.999	-0.30	0.406	0.519	0.22	0.986	0.986	0.00	0.975	0.980	-0.01	0.458	0.998	-0.19
