# Supplementary material for: Changes in the pulmonary surfactant in patients with mild to moderate COVID-19
Source: PLoS One. 2025 Aug 7;20(8):e0325153. doi: 10.1371/journal.pone.0325153 (PMC12331066; doi:10.1371/journal.pone.0325153)
Supplement: S3 Table — (PDF) [file pone.0325153.s004.pdf]

Table S3. List of proteins included in the study.

TargetFullName	UniProt	EntrezGeneSymbol
Alpha-2-HS-glycoprotein	P02765	AHSG
Angiotensinogen	P01019	AGT
Antithrombin-III	P01008	SERPINC1
Beta-1,4-galactosyltransferase 1	P15291	B4GALT1
Beta-2-glycoprotein 1	P02749	APOH
Biotinidase	P43251	BTD
Cartilage acidic protein 1	Q9NQ79	CRTAC1
C-C motif chemokine 18	P55774	CCL18
Complement C3b, inactivated	P01024	C3
Complement C4b	P0C0L4|P0C0L5	C4A|C4B
Complement C5	P01031	C5
Complement decay-accelerating factor	P08174	CD55
Complement factor B	P00751	CFB
Complement factor D	P00746	CFD
Complement factor I	P05156	CFI
Contactin-6	Q9UQ52	CNTN6
EGF-like repeat and discoidin I-like domain-containing protein 3	O43854	EDIL3
Ephrin-A1	P20827	EFNA1
Extracellular superoxide dismutase [Cu-Zn]	P08294	SOD3
Fatty acid-binding protein, heart	P05413	FABP3
Fructose-1,6-bisphosphatase 1	P09467	FBP1
Gelsolin	P06396	GSN
Haptoglobin isoform 2	P00738	HP
Hemopexin	P02790	HPX
Inter-alpha-trypsin inhibitor heavy chain H2	P19823	ITIH2
Interleukin-6 receptor subunit beta	P40189	IL6ST
Low affinity immunoglobulin gamma Fc region receptor III-B	O75015	FCGR3B
Lumican	P51884	LUM
Macrophage colony-stimulating factor 1 receptor	P07333	CSF1R
Macrophage mannose receptor 1	P22897	MRC1
Neuroligin-1	Q8N2Q7	NLGN1
Neurotrimin	Q9P121	NTM
Noggin	Q13253	NOG
Plasma protease C1 inhibitor	P05155	SERPING1
Plexin-D1	Q9Y4D7	PLXND1
Prothrombin	P00734	F2
Pulmonary surfactant-associated protein D	P35247	SFTPD
Receptor-type tyrosine-protein phosphatase S	Q13332	PTPRS
Retinol-binding protein 4	P02753	RBP4
Serotransferrin	P02787	TF
Serum albumin	P02768	ALB
Thyroxine-binding globulin	P05543	SERPINA7
Transmembrane protein 2	Q9UHN6	CEMIP2
Triggering receptor expressed on myeloid cells 2	Q9NZC2	TREM2
Vitamin D-binding protein	P02774	GC
V-set and immunoglobulin domain-containing protein 4	Q9Y279	VSIG4
