# Supplementary material for: Changes in the pulmonary surfactant in patients with mild to moderate COVID-19
Source: PLoS One. 2025 Aug 7;20(8):e0325153. doi: 10.1371/journal.pone.0325153 (PMC12331066; doi:10.1371/journal.pone.0325153)
Supplement: S4 Table — (COVID-19 cases at baseline and follow-up and healthy controls). (PDF) [file pone.0325153.s005.pdf]

Table S4. List of proteins detected in more than 50% of all samples for each group separately (COVID-19 cases at baseline and follow-up and healthy controls).

			Detected >LOD in more than 50% of samples		
**TargetFullName**	**UniProt**	**EntrezGeneSymbol**	**COVID-19 at baseline**	**COVID-19 at follow-up**	**Healthy controls**
Contactin-6	Q9UQ52	CNTN6	YES	YES	YES
Serum albumin	P02768	ALB	YES	YES	YES
Antithrombin-III	P01008	SERPINC1	YES	YES	YES
Biotinidase	P43251	BTD	YES	YES	YES
Cartilage acidic protein 1	Q9NQ79	CRTAC1	YES	YES	YES
C-C motif chemokine 18	P55774	CCL18	YES	YES	YES
Complement factor I	P05156	CFI	YES	YES	YES
Gelsolin	P06396	GSN	YES	YES	YES
Inter-alpha-trypsin inhibitor heavy chain H2	P19823	ITIH2	YES	YES	YES
Low affinity immunoglobulin gamma Fc region receptor III-B	O75015	FCGR3B	YES	YES	YES
Plasma protease C1 inhibitor	P05155	SERPING1	YES	YES	YES
Plexin-D1	Q9Y4D7	PLXND1	YES	YES	YES
Prothrombin	P00734	F2	YES	YES	YES
Pulmonary surfactant-associated protein D	P35247	SFTPD	YES	YES	YES
Serotransferrin	P02787	TF	YES	YES	YES
Triggering receptor expressed on myeloid cells 2	Q9NZC2	TREM2	YES	YES	YES
Fatty acid-binding protein, heart	P05413	FABP3	YES	YES	YES
Lumican	P51884	LUM	YES	YES	YES
V-set and immunoglobulin domain-containing protein 4	Q9Y279	VSIG4	YES	YES	YES
Receptor-type tyrosine-protein phosphatase S	Q13332	PTPRS	YES	YES	YES
Hemopexin	P02790	HPX	YES	YES	YES
Complement C4b	P0C0L4|P0C0L5	C4A|C4B	YES	YES	YES
Ephrin-A1	P20827	EFNA1	YES	YES	YES
Macrophage mannose receptor 1	P22897	MRC1	YES	YES	YES
Angiotensinogen	P01019	AGT	YES	YES	YES
Complement C3b, inactivated	P01024	C3	YES	YES	YES
Thyroxine-binding globulin	P05543	SERPINA7	YES	YES	YES
Retinol-binding protein 4	P02753	RBP4	YES	YES	YES
Noggin	Q13253	NOG	YES	YES	YES
Vitamin D-binding protein	P02774	GC	YES	YES	YES
Complement factor B	P00751	CFB	YES	YES	YES
Beta-2-glycoprotein 1	P02749	APOH	NO	YES	YES
Beta-1,4-galactosyltransferase 1	P15291	B4GALT1	YES	YES	YES
Extracellular superoxide dismutase [Cu-Zn]	P08294	SOD3	NO	YES	YES
Alpha-2-HS-glycoprotein	P02765	AHSG	YES	YES	YES
Complement factor D	P00746	CFD	NO	YES	YES
Transmembrane protein 2	Q9UHN6	CEMIP2	NO	YES	YES
Complement C5	P01031	C5	YES	YES	YES
Complement decay-accelerating factor	P08174	CD55	YES	YES	YES
EGF-like repeat and discoidin I-like domain-containing protein 3	O43854	EDIL3	YES	YES	YES
Interleukin-6 receptor subunit beta	P40189	IL6ST	YES	YES	YES
Haptoglobin isoform 2	P00738	HP	YES	NO	YES
Sex hormone-binding globulin	P04278	SHBG	NO	NO	YES
Fetuin-B	Q9UGM5	FETUB	NO	NO	YES

Fructose-1,6-bisphosphatase 1	P09467	FBP1	NO	YES	NO
Macrophage colony-stimulating factor 1 receptor	P07333	CSF1R	YES	NO	NO
Poliovirus receptor	P15151	PVR	NO	NO	NO
Neuroligin-1	Q8N2Q7	NLGN1	YES	YES	NO
Neurotrimin	Q9P121	NTM	YES	YES	NO
Paired immunoglobulin-like type 2 receptor alpha isoform FDF03-d	Q9UKJ1	PILRA	NO	NO	NO
Frizzled-7	O75084	FZD7	NO	YES	NO
Complement C4	P0C0L4|P0C0L5	C4A|C4B	NO	NO	NO
