# Supplementary material for: Changes in the pulmonary surfactant in patients with mild to moderate COVID-19
Source: PLoS One. 2025 Aug 7;20(8):e0325153. doi: 10.1371/journal.pone.0325153 (PMC12331066; doi:10.1371/journal.pone.0325153)
Supplement: S5 Dataset — (PDF) [file pone.0325153.s006.pdf]

Subject_ID	Visit	CovidCOVI	Sex	Age_interva	Height	Weight	R5	R5_Zscore	R5R20
1	1	COVID	0	71-80	167.0	52.0	4.05500	1.48200	-0.33500
1	2	COVID	0	71-80	167.0	52.0	3.46700	0.88160	-0.39000
10	1	COVID	1	51-60	185.0	82.0	2.83600	1.37500	0.79600
10	2	COVID	1	51-60	185.0	83.0	2.42100	0.76530	0.63000
11	1	COVID	0	31-40	170.0	65.0	2.77700	-0.03139	0.10600
11	2	COVID	0	31-40	169.0	64.0	2.93500	0.16460	-0.03700
12	1	COVID	0	21-30	165.0	60.0	2.41200	-0.45980	0.06600
12	2	COVID	0	21-30	167.0	65.0	3.08900	0.34830	0.48000
13	1	Control	0	31-40	168.0	55.0	2.08900	-0.73660	-0.24700
14	1	Control	0	21-30	165.0	72.0	2.85500	-0.41540	-0.01000
16	1	Control	1	21-30	185.0	64.0	1.24800	-1.03500	0.03300
17	1	Control	1	51-60	185.0	89.0	1.52700	-1.16300	0.07800
18	1	COVID	0	31-40	172.0	57.0	4.16300	2.02000	0.14500
19	1	COVID	0	41-50	164.0	58.0	2.29700	-0.81750	-0.54400
2	1	COVID	1	71-80	172.0	68.0	4.58200	2.56600	0.43400
2	2	COVID	1	71-80	172.0	68.0	3.58700	1.70600	0.22700
20	1	Control	0	61-70	169.0	68.0	2.64200	-0.66840	-0.00600
21	1	Control	0	61-70	166.0	69.0	5.64300	1.92300	0.30400
22	1	Control	1	41-50	194.0	90.0	1.96500	0.43920	-0.06900
23	1	Control	1	51-60	187.0	85.0	1.76600	-0.22210	-0.02400
24	1	Control	0	51-60	169.0	56.0	3.61000	1.09200	0.14400
25	1	Control	1	51-60	191.0	82.0	1.79300	0.40610	0.01300
26	1	Control	0	51-60	167.0	59.0	2.44100	-0.62280	-0.71400
27	1	Control	1	71-80	185.0	99.0	2.53500	0.32680	0.14500
28	1	Control	0	31-40	162.0	64.0	3.19100	0.14110	-0.07800
29	1	Control	0	31-40	172.0	86.0	2.97700	-0.57660	-0.47700
3	1	COVID	0	41-50	169.0	59.0	3.15700	0.59020	0.44300
3	2	COVID	0	41-50	169.0	60.0	2.48200	-0.36430	-0.10900
30	1	COVID	1	31-40	172.0	61.0	2.83800	0.66130	0.19000
31	1	COVID	0	31-40	170.0	65.0	3.01400	0.35100	0.99700
31	2	COVID	0	31-40	170.0	65.0	3.49800	0.90600	0.88700
32	1	COVID	0	41-50	167.0	56.0	5.09600	2.43000	1.01600
32	2	COVID	0	41-50	167.0	60.0	3.67800	1.01200	-0.04300
33	1	COVID	1	61-70	171.0	73.0	2.04100	-0.71650	0.08400
33	2	COVID	1	61-70	171.0	71.0	1.60000	-1.47600	-0.26700
34	1	COVID	0	31-40	177.0	66.0	2.62000	0.08486	-0.11700
35	1	COVID	1	31-40	190.0	106.0	3.65700	1.29900	0.49100
35	2	COVID	1	31-40	190.0	112.0	2.56300	-0.25870	-0.13800
36	1	COVID	0	41-50	164.0	57.0	3.46800	0.81480	-0.00200
37	1	COVID	1	31-40	188.0	95.0	3.77000	1.71800	0.02100
37	2	COVID	1	31-40	187.0	96.0	3.87100	1.66200	0.47300
38	1	COVID	1	31-40	175.0	80.0	3.16600	0.46080	0.37000
38	2	COVID	1	31-40	175.0	80.0	2.38400	-0.54310	0.00900
39	1	COVID	0	41-50	169.0	88.0	3.31000	-0.60410	1.00200
39	2	COVID	0	51-60	169.0	82.0	2.95300	-0.76420	0.57800
4	1	COVID	1	41-50	189.0	74.0	2.39500	1.47700	-0.17000

4	2	COVID	1	41-50	189.0	75.0	2.37100	1.39400	-0.03000
40	1	COVID	1	31-40	182.0	75.0	2.48900	0.68840	-0.06500
40	2	COVID	1	31-40	182.0	78.0	2.05200	-0.14270	-0.00600
41	1	COVID	0	21-30	164.0	69.0	2.86000	-0.30000	-0.35400
41	2	COVID	0	21-30	164.0	69.0	2.73900	-0.46930	-0.23100
42	1	COVID	1	21-30	179.0	71.0	2.47300	0.35090	-0.19100
42	2	COVID	1	21-30	179.0	75.0	2.23900	-0.19460	-0.45100
43	1	COVID	0	31-40	168.0	54.0	2.09400	-0.68760	-0.37900
43	2	COVID	0	31-40	168.0	54.0	2.22100	-0.47110	-0.31300
44	1	COVID	0	21-30	170.0	58.0	4.06900	1.82800	-0.75600
45	1	Control	0	21-30	172.0	63.0	2.65300	0.12200	-0.13900
46	1	Control	1	21-30	198.0	59.0	1.77200	1.82100	-0.75000
47	1	Control	1	21-30	188.0	87.0	2.32600	0.34060	-0.05100
5	1	COVID	1	21-30	181.0	78.0	1.91400	-0.68760	-0.02700
5	2	COVID	1	21-30	182.0	76.0	2.14100	-0.07548	-0.03200
6	1	COVID	0	61-70	165.0	65.0	2.91900	-0.37690	0.23600
6	2	COVID	0	61-70	165.0	63.0	4.23800	1.11500	0.80100
7	1	COVID	1	61-70	174.0	89.0	4.55200	1.60400	0.93200
7	2	COVID	1	61-70	175.0	92.0	3.16100	0.26980	0.24400
8	1	COVID	0	61-70	167.0	63.5	8.16600	3.70300	0.64600
8	2	COVID	0	61-70	167.0	66.0	8.60600	3.78000	0.97900
9	1	COVID	0	51-60	170.0	75.0	6.08100	2.23300	1.38500

R5R20_Zsc	AX	AX_Zscore	Nr_breath	Tot_mass	Mass_samplec	Nr_conc	Mass_conc	Averagema
-0.29060	2.8720	0.38020	20	145.53720	123.86042	16435.04641	3.99886	0.24331
-0.44840	3.3700	0.59680	12	140.18950	119.48589	20590.13033	4.89169	0.23757
2.36000	6.2460	2.25800	15	143.47393	123.60079	8772.63992	2.49314	0.28419
1.91600	2.3870	0.90430	6	138.46693	118.65303	21072.42476	5.28298	0.25071
0.82890	2.0580	0.00135	23	97.29182	83.73358	6788.99141	1.88707	0.27796
0.45640	1.2190	-0.77560	13	142.12006	120.64232	18509.10519	4.12388	0.22280
0.94140	1.9470	0.02094	15	49.62532	42.68936	5446.33893	1.51771	0.27867
1.92200	3.2900	0.68750	21	142.75807	119.77945	9120.72221	1.77274	0.19436
0.18540	2.7340	0.72210	7	143.33153	121.63418	32639.57894	7.47342	0.22897
0.23600	2.3820	-0.22900	17	150.87502	126.35920	16650.99878	3.26983	0.19637
0.73240	0.8540	0.04464	20	63.26468	53.98214	4378.75946	1.07983	0.24661
0.36910	0.9710	-0.50820	23	96.35949	84.03409	6986.86926	2.52590	0.36152
1.26800	3.8900	1.42900	14	56.61390	47.45814	11998.93731	2.38323	0.19862
-0.85600	0.3240	-2.81600	14	47.74848	40.97582	7166.78870	2.04325	0.28510
1.02800	7.9700	1.90900	3	154.10987	134.62147	49245.26876	17.98211	0.36515
0.50330	7.1850	1.76600	14	141.80143	121.27436	11779.09746	2.74696	0.23321
0.10960	4.0630	0.46040	4	491.97428	421.84711	###########	42.59167	0.26047
0.74860	7.1840	0.99200	5	151.29667	132.49086	44238.80882	17.15754	0.38784
0.42140	0.7790	-0.08530	9	146.26601	124.64253	12808.06775	3.07185	0.23984
0.31570	1.4230	0.30260	4	177.83628	151.87519	50248.83165	12.37474	0.24627
1.01600	6.1790	1.59900	6	146.43977	125.77419	32158.23376	8.74328	0.27188
0.64060	1.1420	0.42300	7	146.58913	126.42251	15333.45413	4.45434	0.29050
-1.44400	1.4320	-0.73990	5	165.01069	140.53068	60119.36558	14.18377	0.23593
0.29700	2.8040	0.65850	6	160.08564	138.10857	23918.75390	6.95341	0.29071
0.24280	2.0970	-0.34900	7	146.53486	126.37402	25545.69837	7.21392	0.28239
-1.55000	1.4070	-1.22400	7	144.37984	120.97870	27328.89381	5.29893	0.19389
1.85300	8.5500	2.13000	6	159.00321	137.24825	24231.67782	6.88845	0.28427
0.32340	2.5990	0.36980	3	143.37224	122.27667	51886.42132	12.50126	0.24094
0.48840	5.1420	1.51000	#NULL!	#NULL!	#NULL!	#NULL!	#NULL!	#NULL!
3.30800	7.5260	1.96700	#NULL!	#NULL!	#NULL!	#NULL!	#NULL!	#NULL!
3.00500	4.7780	1.30400	18	99.80395	82.43193	7996.49524	1.38391	0.17306
3.47800	20.0580	3.37400	7	145.74726	123.15814	29022.22801	6.13808	0.21150
0.46970	4.0040	0.88170	15	147.51878	123.53916	17904.01560	3.41900	0.19096
-0.11220	5.7460	1.22900	4	139.13846	118.40165	36373.79025	8.56432	0.23545
-0.93500	3.0560	0.41830	9	152.63902	129.44811	22632.84632	5.04567	0.22294
0.31160	1.3500	-0.23790	10	151.13563	128.96974	18944.74382	4.35964	0.23012
1.20600	8.6190	2.42000	11	141.37247	119.34573	13649.39493	2.57401	0.18858
-0.60460	2.5930	0.59180	18	148.26909	121.13001	9679.77736	1.51726	0.15675
0.68150	3.5140	0.70500	20	144.38774	126.69303	9705.45514	3.86845	0.39858
0.19500	4.2440	1.59800	13	54.75638	46.62885	3707.32471	0.82957	0.22377
1.24500	5.0000	1.71000	24	98.65312	82.23175	3823.39252	0.72378	0.18930
0.54730	9.8060	2.09600	17	98.16278	82.66212	6063.74636	1.26198	0.20812
-0.36770	2.9700	0.45250	17	98.24646	81.30415	7057.80057	1.20820	0.17119
1.97900	7.6770	0.73730	14	100.74831	86.64406	9519.44790	2.64525	0.27788
1.16700	2.6570	-0.55370	17	106.75395	91.57896	8255.26406	2.18669	0.26488
0.21680	1.8280	1.13600	6	154.18443	130.67223	24440.43815	5.23242	0.21409

0.55880	1.2710	0.60720	6	162.87360	139.27691	26802.27557	6.52900	0.24360
0.13260	1.7200	0.43510	6	148.06517	127.01425	24593.77924	6.11967	0.24883
0.20910	0.8830	-0.56990	9	127.51633	106.56969	12391.24810	2.34351	0.18913
-0.56180	2.8610	0.13250	16	97.49857	81.55248	8872.66303	1.69166	0.19066
-0.23670	2.5960	-0.01515	17	145.24978	120.95817	12135.82225	2.23019	0.18377
-0.30020	1.4110	0.02718	16	96.85956	80.82170	6444.06830	1.10559	0.17157
-1.07400	1.0810	-0.45650	26	147.97858	121.81869	6055.83455	0.99630	0.16452
-0.14710	2.6920	0.73160	6	169.18173	145.94237	35427.69582	9.86536	0.27846
0.02873	2.7200	0.74120	5	144.45231	122.82388	44087.99959	10.27449	0.23304
-1.18400	1.0150	-0.60060	16	104.50233	89.90406	7576.61466	2.01129	0.26546
0.38000	1.4970	-0.09454	27	94.71699	80.67631	6118.12088	1.49660	0.24462
-0.88070	0.6290	0.86300	18	73.86707	62.69431	4921.76464	1.09479	0.22244
0.21250	1.0780	-0.05454	16	96.59928	81.48421	5872.65612	1.10943	0.18891
0.05832	1.7920	0.31960	6	139.16565	118.00069	20470.06184	4.56221	0.22287
0.16800	1.2490	-0.03519	12	148.06552	122.89653	12684.11741	2.24272	0.17681
0.78440	8.6020	1.42300	3	170.23181	144.55973	84897.83006	19.17350	0.22584
2.39600	15.1130	2.31300	6	159.51020	133.52042	48515.69962	9.33293	0.19237
1.67500	21.5670	2.83400	6	139.03740	119.46178	41667.42386	11.04807	0.26515
-0.10420	9.2930	1.67200	5	152.52530	129.40046	38458.55551	8.33914	0.21683
2.04300	6.8540	1.31500	9	153.02792	132.88880	22287.39213	7.03268	0.31555
2.83100	17.0110	2.51500	7	158.32472	135.34790	30132.72431	7.44902	0.24721
3.61500	18.8230	2.48300	18	147.51034	126.64512	11615.47660	3.11585	0.26825

Smokingst	Years_smc	Vaccinerac	Andningsb	Brostmarte	Hosta	Feber	Snuva	Nastappa	Muskel_le
1	31	0	1	0	1	1	1	0	0
1	32	1	1	0	1	1	1	0	0
0	0	0	1	0	1	1	1	0	1
0	0	1	1	0	1	1	1	0	1
0	0	1	0	0	1	1	1	1	0
0	0	1	0	0	1	1	1	1	0
0	0	1	0	0	0	0	1	1	0
0	0	1	0	0	0	0	1	1	0
0	0	1	#NULL!	#NULL!	#NULL!	#NULL!	#NULL!	#NULL!	#NULL!
0	0	1	#NULL!	#NULL!	#NULL!	#NULL!	#NULL!	#NULL!	#NULL!
0	0	1	#NULL!	#NULL!	#NULL!	#NULL!	#NULL!	#NULL!	#NULL!
0	0	1	#NULL!	#NULL!	#NULL!	#NULL!	#NULL!	#NULL!	#NULL!
0	0	0	0	0	0	1	1	1	0
1	17	1	0	1	0	0	1	1	0
1	47	0	0	0	0	0	0	0	0
1	48	1	0	0	0	0	0	0	0
0	0	1	#NULL!	#NULL!	#NULL!	#NULL!	#NULL!	#NULL!	#NULL!
0	0	1	#NULL!	#NULL!	#NULL!	#NULL!	#NULL!	#NULL!	#NULL!
0	0	1	#NULL!	#NULL!	#NULL!	#NULL!	#NULL!	#NULL!	#NULL!
0	0	1	#NULL!	#NULL!	#NULL!	#NULL!	#NULL!	#NULL!	#NULL!
1	20	1	#NULL!	#NULL!	#NULL!	#NULL!	#NULL!	#NULL!	#NULL!
0	0	1	#NULL!	#NULL!	#NULL!	#NULL!	#NULL!	#NULL!	#NULL!
0	0	1	#NULL!	#NULL!	#NULL!	#NULL!	#NULL!	#NULL!	#NULL!
0	0	1	#NULL!	#NULL!	#NULL!	#NULL!	#NULL!	#NULL!	#NULL!
0	0	1	#NULL!	#NULL!	#NULL!	#NULL!	#NULL!	#NULL!	#NULL!
0	0	1	#NULL!	#NULL!	#NULL!	#NULL!	#NULL!	#NULL!	#NULL!
0	0	0	1	1	1	1	1	1	1
0	0	1	1	1	1	1	1	1	1
0	0	1	0	0	1	1	1	1	0
0	0	1	1	0	1	1	1	1	1
0	0	1	1	0	1	1	1	1	1
0	0	1	1	0	1	1	1	0	1
0	1	1	1	0	1	1	1	0	1
1	43	1	0	0	1	1	1	1	0
1	44	1	0	0	1	1	1	1	0
1	17	0	999	1	1	1	1	1	1
0	0	0	1	0	1	0	1	1	1
0	1	0	1	0	1	0	1	1	1
1	19	0	0	0	1	1	0	1	1
0	0	0	0	0	1	1	1	0	1
0	1	0	0	0	1	1	1	0	1
0	0	0	0	0	1	1	1	1	1
0	1	1	0	0	1	1	1	1	1
1	8	1	0	0	1	1	1	1	1
1	9	1	0	0	1	1	1	1	1
0	0	0	0	1	1	0	0	0	1

0	0	1	0	1	1	0	0	0	1
0	0	1	0	1	1	1	1	1	1
0	0	1	0	1	1	1	1	1	1
0	0	1	0	0	0	2	1	1	0
0	0	1	0	0	0	2	1	1	0
0	0	1	0	0	1	0	1	1	0
0	0	1	0	0	1	0	1	1	0
0	0	1	0	0	1	1	1	1	0
0	0	1	0	0	1	1	1	1	0
0	0	1	0	0	1	1	1	1	1
0	0	1	#NULL!	#NULL!	#NULL!	#NULL!	#NULL!	#NULL!	#NULL!
0	0	1	#NULL!	#NULL!	#NULL!	#NULL!	#NULL!	#NULL!	#NULL!
0	0	1	#NULL!	#NULL!	#NULL!	#NULL!	#NULL!	#NULL!	#NULL!
0	0	0	0	0	1	1	1	1	1
0	0	0	0	0	1	1	1	1	1
1	33	0	1	0	1	1	1	1	1
1	33	1	1	0	1	1	1	1	1
1	35	0	0	0	1	1	1	1	1
1	35	1	0	0	1	1	1	1	1
1	29	0	1	1	1	1	1	1	0
1	29	1	1	1	1	1	1	1	0
0	0	0	0	0	0	1	0	1	0

Halsont	Diarre	Huvudvark	Nedsattluk	Vasandear	mMRC	Hypertens	Cardiacdis	Lungdiseas	Asthma
1	1	1	1	0	3	0	0	0	0
1	1	1	1	0	1	0	0	0	0
0	1	0	0	1	4	0	0	0	0
0	1	0	0	1	1	0	0	0	0
1	0	0	1	0	0	0	0	0	0
1	0	0	1	0	0	0	0	0	0
0	0	1	1	0	1	0	0	0	0
0	0	1	1	0	0	0	0	0	0
#NULL!	#NULL!	#NULL!	#NULL!	#NULL!	0	0	0	0	0
#NULL!	#NULL!	#NULL!	#NULL!	#NULL!	0	0	0	0	0
#NULL!	#NULL!	#NULL!	#NULL!	#NULL!	0	0	0	0	0
#NULL!	#NULL!	#NULL!	#NULL!	#NULL!	0	0	0	0	0
1	0	1	0	0	0	0	0	0	0
1	0	1	1	0	0	0	0	0	0
0	0	1	1	0	0	0	0	0	0
0	0	1	1	0	0	0	0	0	0
#NULL!	#NULL!	#NULL!	#NULL!	#NULL!	0	0	0	0	0
#NULL!	#NULL!	#NULL!	#NULL!	#NULL!	0	0	0	0	0
#NULL!	#NULL!	#NULL!	#NULL!	#NULL!	0	0	0	0	0
#NULL!	#NULL!	#NULL!	#NULL!	#NULL!	0	0	0	0	0
#NULL!	#NULL!	#NULL!	#NULL!	#NULL!	1	0	0	0	0
#NULL!	#NULL!	#NULL!	#NULL!	#NULL!	2	0	0	0	0
#NULL!	#NULL!	#NULL!	#NULL!	#NULL!	0	0	0	0	0
#NULL!	#NULL!	#NULL!	#NULL!	#NULL!	1	1	0	0	0
#NULL!	#NULL!	#NULL!	#NULL!	#NULL!	0	0	0	0	0
#NULL!	#NULL!	#NULL!	#NULL!	#NULL!	0	0	0	0	1
1	1	1	1	0	3	0	0	0	0
1	1	1	1	0	0	0	0	0	0
0	0	1	0	1	1	0	0	0	0
0	0	1	1	0	3	0	0	0	0
0	0	1	1	0	1	0	0	0	0
1	0	1	0	1	3	0	0	0	1
1	0	1	0	1	0	0	0	0	1
1	0	0	1	0	0	0	0	0	0
1	0	0	1	0	0	0	0	0	0
1	0	1	1	0	2	0	0	0	0
0	1	1	1	0	1	0	0	0	0
0	1	1	1	0	1	0	0	0	0
0	0	0	1	0	4	0	0	0	0
0	0	0	1	0	1	0	0	0	0
0	0	0	1	0	#NULL!	0	0	0	0
1	0	1	1	0	1	0	0	0	0
1	0	1	1	0	1	0	0	0	0
1	0	1	1	0	2	0	0	0	0
1	0	1	1	0	1	0	0	0	0
0	1	0	1	0	0	0	0	0	0

0	1	0	1	0	0	0	0	0	0
0	0	0	1	0	2	0	0	0	0
0	0	0	1	0	0	0	0	0	0
1	0	0	0	0	1	0	0	0	1
1	0	0	0	0	0	0	0	0	0
1	0	1	0	0	1	0	0	0	0
1	0	1	0	0	1	0	0	0	0
1	0	0	0	0	0	0	0	0	0
1	0	0	0	0	0	0	0	0	0
0	0	1	0	0	0	0	0	0	0
#NULL!	#NULL!	#NULL!	#NULL!	#NULL!	0	0	0	0	0
#NULL!	#NULL!	#NULL!	#NULL!	#NULL!	0	0	0	0	0
#NULL!	#NULL!	#NULL!	#NULL!	#NULL!	0	0	0	0	0
1	0	0	1	1	0	0	0	0	0
1	0	0	1	1	0	0	0	0	0
1	0	1	1	0	4	0	0	0	0
1	0	1	1	0	1	0	0	0	0
1	1	1	1	1	2	1	0	0	0
1	1	1	1	1	2	1	0	0	0
1	1	1	1	0	0	1	0	0	0
1	1	1	1	0	0	1	0	0	0
0	1	1	1	0	4	1	0	0	0

Diabetes	FU_febere	FU_torrho	FU_slemhc	FU_andnin	FU_brosts	FU_yrsel	FU_trotthe	FU_viktne	FU_livskva
0	#NULL!	#NULL!	#NULL!	#NULL!	#NULL!	#NULL!	#NULL!	#NULL!	#NULL!
0	1	0	1	0	0	0	1	1	0
0	#NULL!	#NULL!	#NULL!	#NULL!	#NULL!	#NULL!	#NULL!	#NULL!	#NULL!
0	0	0	0	1	0	0	0	0	1
0	#NULL!	#NULL!	#NULL!	#NULL!	#NULL!	#NULL!	#NULL!	#NULL!	#NULL!
0	0	0	0	0	0	0	0	0	0
0	#NULL!	#NULL!	#NULL!	#NULL!	#NULL!	#NULL!	#NULL!	#NULL!	#NULL!
0	0	0	0	0	0	0	0	0	0
0	#NULL!	#NULL!	#NULL!	#NULL!	#NULL!	#NULL!	#NULL!	#NULL!	#NULL!
0	#NULL!	#NULL!	#NULL!	#NULL!	#NULL!	#NULL!	#NULL!	#NULL!	#NULL!
0	#NULL!	#NULL!	#NULL!	#NULL!	#NULL!	#NULL!	#NULL!	#NULL!	#NULL!
0	#NULL!	#NULL!	#NULL!	#NULL!	#NULL!	#NULL!	#NULL!	#NULL!	#NULL!
0	#NULL!	#NULL!	#NULL!	#NULL!	#NULL!	#NULL!	#NULL!	#NULL!	#NULL!
0	#NULL!	#NULL!	#NULL!	#NULL!	#NULL!	#NULL!	#NULL!	#NULL!	#NULL!
0	#NULL!	#NULL!	#NULL!	#NULL!	#NULL!	#NULL!	#NULL!	#NULL!	#NULL!
0	0	1	1	0	0	0	1	0	0
0	#NULL!	#NULL!	#NULL!	#NULL!	#NULL!	#NULL!	#NULL!	#NULL!	#NULL!
0	#NULL!	#NULL!	#NULL!	#NULL!	#NULL!	#NULL!	#NULL!	#NULL!	#NULL!
0	#NULL!	#NULL!	#NULL!	#NULL!	#NULL!	#NULL!	#NULL!	#NULL!	#NULL!
0	#NULL!	#NULL!	#NULL!	#NULL!	#NULL!	#NULL!	#NULL!	#NULL!	#NULL!
0	#NULL!	#NULL!	#NULL!	#NULL!	#NULL!	#NULL!	#NULL!	#NULL!	#NULL!
0	#NULL!	#NULL!	#NULL!	#NULL!	#NULL!	#NULL!	#NULL!	#NULL!	#NULL!
0	#NULL!	#NULL!	#NULL!	#NULL!	#NULL!	#NULL!	#NULL!	#NULL!	#NULL!
0	#NULL!	#NULL!	#NULL!	#NULL!	#NULL!	#NULL!	#NULL!	#NULL!	#NULL!
0	#NULL!	#NULL!	#NULL!	#NULL!	#NULL!	#NULL!	#NULL!	#NULL!	#NULL!
0	#NULL!	#NULL!	#NULL!	#NULL!	#NULL!	#NULL!	#NULL!	#NULL!	#NULL!
0	#NULL!	#NULL!	#NULL!	#NULL!	#NULL!	#NULL!	#NULL!	#NULL!	#NULL!
0	0	0	0	1	0	0	1	0	0
0	#NULL!	#NULL!	#NULL!	#NULL!	#NULL!	#NULL!	#NULL!	#NULL!	#NULL!
0	#NULL!	#NULL!	#NULL!	#NULL!	#NULL!	#NULL!	#NULL!	#NULL!	#NULL!
0	0	0	0	1	0	1	1	0	0
0	#NULL!	#NULL!	#NULL!	#NULL!	#NULL!	#NULL!	#NULL!	#NULL!	#NULL!
0	0	1	0	0	0	0	1	0	0
0	#NULL!	#NULL!	#NULL!	#NULL!	#NULL!	#NULL!	#NULL!	#NULL!	#NULL!
0	0	0	0	0	0	0	0	0	0
0	#NULL!	#NULL!	#NULL!	#NULL!	#NULL!	#NULL!	#NULL!	#NULL!	#NULL!
0	#NULL!	#NULL!	#NULL!	#NULL!	#NULL!	#NULL!	#NULL!	#NULL!	#NULL!
0	0	0	0	0	0	0	0	0	0
0	#NULL!	#NULL!	#NULL!	#NULL!	#NULL!	#NULL!	#NULL!	#NULL!	#NULL!
0	#NULL!	#NULL!	#NULL!	#NULL!	#NULL!	#NULL!	#NULL!	#NULL!	#NULL!
0	0	0	0	0	0	0	0	0	0
0	#NULL!	#NULL!	#NULL!	#NULL!	#NULL!	#NULL!	#NULL!	#NULL!	#NULL!
0	0	0	0	0	0	0	0	0	0
0	#NULL!	#NULL!	#NULL!	#NULL!	#NULL!	#NULL!	#NULL!	#NULL!	#NULL!
0	0	0	0	0	0	0	0	0	0
0	#NULL!	#NULL!	#NULL!	#NULL!	#NULL!	#NULL!	#NULL!	#NULL!	#NULL!

0	0	0	0	0	0	0	0	0	0
0	#NULL!	#NULL!	#NULL!	#NULL!	#NULL!	#NULL!	#NULL!	#NULL!	#NULL!
0	0	0	0	0	0	0	0	0	0
0	#NULL!	#NULL!	#NULL!	#NULL!	#NULL!	#NULL!	#NULL!	#NULL!	#NULL!
0	0	0	0	0	0	0	0	0	0
0	#NULL!	#NULL!	#NULL!	#NULL!	#NULL!	#NULL!	#NULL!	#NULL!	#NULL!
0	0	0	0	0	0	0	0	0	0
0	#NULL!	#NULL!	#NULL!	#NULL!	#NULL!	#NULL!	#NULL!	#NULL!	#NULL!
0	0	0	0	0	0	0	0	0	0
0	#NULL!	#NULL!	#NULL!	#NULL!	#NULL!	#NULL!	#NULL!	#NULL!	#NULL!
0	#NULL!	#NULL!	#NULL!	#NULL!	#NULL!	#NULL!	#NULL!	#NULL!	#NULL!
0	#NULL!	#NULL!	#NULL!	#NULL!	#NULL!	#NULL!	#NULL!	#NULL!	#NULL!
0	#NULL!	#NULL!	#NULL!	#NULL!	#NULL!	#NULL!	#NULL!	#NULL!	#NULL!
0	#NULL!	#NULL!	#NULL!	#NULL!	#NULL!	#NULL!	#NULL!	#NULL!	#NULL!
0	0	0	0	0	0	0	0	0	0
0	#NULL!	#NULL!	#NULL!	#NULL!	#NULL!	#NULL!	#NULL!	#NULL!	#NULL!
0	0	0	1	1	0	0	1	1	0
0	#NULL!	#NULL!	#NULL!	#NULL!	#NULL!	#NULL!	#NULL!	#NULL!	#NULL!
0	0	0	0	1	0	1	1	0	0
0	#NULL!	#NULL!	#NULL!	#NULL!	#NULL!	#NULL!	#NULL!	#NULL!	#NULL!
0	0	0	0	0	0	0	0	0	0
0	#NULL!	#NULL!	#NULL!	#NULL!	#NULL!	#NULL!	#NULL!	#NULL!	#NULL!

HexCerd18	HexCerd18	HexCerd18	HexCerd18	LPC16_01	LPC16_02	LPC16_11	LPC16_12	LPC18_01	LPC18_02
1.00500	1.00290	1.0020	1.0054	1.0464	1.0819	1.0042	1.0009	1.0050	1.0323
1.01470	1.00320	1.0020	1.0055	1.0693	1.0779	1.0048	1.0012	1.0036	1.0297
1.00890	1.00290	1.0018	1.0054	1.1085	1.1846	1.0068	1.0022	1.0077	1.0542
1.00390	1.00140	1.0012	1.0039	1.0647	1.0467	1.0058	1.0008	1.0027	1.0246
1.00920	1.01050	1.0034	1.0115	1.0848	1.1575	1.0057	1.0007	1.0032	1.0332
1.01130	1.00220	1.0016	1.0047	1.0822	1.0489	1.0043	1.0000	1.0032	1.0175
#NULL!	#NULL!	#NULL!	#NULL!	#NULL!	#NULL!	#NULL!	#NULL!	#NULL!	#NULL!
1.00440	1.00190	1.0017	1.0041	1.0592	1.0633	1.0037	1.0000	1.0039	1.0397
1.00270	1.00160	1.0012	1.0032	1.0859	1.0422	1.0061	1.0007	1.0026	1.0148
1.00620	1.00340	1.0019	1.0057	1.0701	1.0532	1.0031	1.0000	1.0031	1.0277
#NULL!	#NULL!	#NULL!	#NULL!	#NULL!	#NULL!	#NULL!	#NULL!	#NULL!	#NULL!
1.00650	1.00450	1.0017	1.0051	1.0720	1.1292	1.0060	1.0014	1.0046	1.0376
#NULL!	#NULL!	#NULL!	#NULL!	#NULL!	#NULL!	#NULL!	#NULL!	#NULL!	#NULL!
#NULL!	#NULL!	#NULL!	#NULL!	#NULL!	#NULL!	#NULL!	#NULL!	#NULL!	#NULL!
1.00810	1.00390	1.0020	1.0058	1.0858	1.0926	1.0087	1.0007	1.0060	1.0336
1.06840	1.05100	1.0265	1.0090	1.1440	1.1461	1.0077	1.0014	1.0088	1.0546
1.00380	1.00250	1.0016	1.0055	1.0678	1.0558	1.0048	1.0013	1.0032	1.0191
1.00590	1.00170	1.0019	1.0043	1.0453	1.1057	1.0025	1.0017	1.0032	1.0292
1.00320	1.00170	1.0011	1.0034	1.0425	1.0451	1.0027	1.0006	1.0017	1.0208
1.00440	1.00200	1.0012	1.0038	1.0425	1.0407	1.0021	1.0004	1.0023	1.0133
1.00520	1.00240	1.0017	1.0048	1.0411	1.0640	1.0056	1.0015	1.0046	1.0332
1.09990	1.02990	1.0205	1.0412	1.0824	1.2056	1.0180	1.0037	1.0171	1.0843
1.00320	1.00130	1.0011	1.0033	1.1343	1.0530	1.0048	1.0016	1.0043	1.0166
1.00460	1.00280	1.0018	1.0050	1.0687	1.0571	1.0051	1.0009	1.0049	1.0250
1.00470	1.00250	1.0016	1.0048	1.0417	1.0354	1.0027	1.0000	1.0024	1.0192
1.00660	1.00260	1.0016	1.0048	1.0765	1.0475	1.0040	1.0000	1.0028	1.0246
1.00720	1.00350	1.0030	1.0070	1.1445	1.0947	1.0119	1.0000	1.0065	1.0382
1.00760	1.00350	1.0037	1.0106	1.0514	1.0420	1.0043	1.0013	1.0015	1.0201
#NULL!	#NULL!	#NULL!	#NULL!	#NULL!	#NULL!	#NULL!	#NULL!	#NULL!	#NULL!
#NULL!	#NULL!	#NULL!	#NULL!	#NULL!	#NULL!	#NULL!	#NULL!	#NULL!	#NULL!
1.00430	1.00190	1.0012	1.0041	1.1625	1.0858	1.0071	1.0005	1.0049	1.0383
1.00330	1.00160	1.0011	1.0037	1.0577	1.0432	1.0028	1.0000	1.0019	1.0287
1.00320	1.00170	1.0012	1.0037	1.0956	1.0478	1.0055	1.0004	1.0036	1.0343
1.00490	1.00130	1.0012	1.0034	1.0700	1.0533	1.0029	1.0010	1.0032	1.0226
1.00540	1.00150	1.0010	1.0037	1.0954	1.0775	1.0046	1.0015	1.0070	1.0512
1.00450	1.00190	1.0014	1.0035	1.0662	1.0795	1.0027	1.0005	1.0035	1.0365
1.00560	1.00260	1.0018	1.0042	1.0652	1.0595	1.0046	1.0017	1.0024	1.0259
1.00380	1.00230	1.0015	1.0032	1.1017	1.0477	1.0053	1.0005	1.0027	1.0234
1.00510	1.00210	1.0016	1.0050	1.1012	1.0469	1.0047	1.0000	1.0034	1.0192
#NULL!	#NULL!	#NULL!	#NULL!	#NULL!	#NULL!	#NULL!	#NULL!	#NULL!	#NULL!
1.00520	1.00260	1.0017	1.0043	1.1861	1.1222	1.0071	1.0011	1.0094	1.0625
1.00540	1.00290	1.0013	1.0045	1.1019	1.1141	1.0043	1.0014	1.0090	1.0830
1.00800	1.00290	1.0019	1.0046	1.1134	1.1074	1.0081	1.0010	1.0065	1.0466
1.00650	1.00370	1.0020	1.0042	1.0746	1.1012	1.0049	1.0012	1.0047	1.0387
1.00560	1.00200	1.0014	1.0037	1.1234	1.0850	1.0070	1.0007	1.0047	1.0372
1.00470	1.00150	1.0012	1.0037	1.1575	1.0912	1.0078	1.0009	1.0076	1.0404

1.00520	1.00160	1.0009	1.0030	1.0633	1.0618	1.0028	1.0000	1.0038	1.0274
1.00690	1.00230	1.0013	1.0039	1.0561	1.0996	1.0031	1.0000	1.0057	1.0286
1.00950	1.00780	1.0035	1.0080	1.0920	1.1737	1.0037	1.0012	1.0105	1.0550
1.00960	1.00390	1.0021	1.0049	1.0615	1.1755	1.0034	1.0009	1.0069	1.0493
1.00370	1.00220	1.0017	1.0046	1.0395	1.0722	1.0023	1.0001	1.0025	1.0333
1.00830	1.00350	1.0015	1.0053	1.0649	1.0836	1.0033	1.0000	1.0036	1.0509
1.00940	1.00280	1.0018	1.0109	1.5336	3.0982	1.0050	1.0063	1.1772	1.8570
1.00730	1.00200	1.0014	1.0040	1.0600	1.0496	1.0028	1.0007	1.0035	1.0200
1.00370	1.00200	1.0013	1.0036	1.0730	1.0431	1.0056	1.0005	1.0022	1.0121
1.00880	1.00270	1.0021	1.0069	1.0560	1.0969	1.0029	1.0006	1.0047	1.0561
1.00440	1.00180	1.0013	1.0053	1.0688	1.0786	1.0031	1.0010	1.0051	1.0340
#NULL!	#NULL!	#NULL!	#NULL!	#NULL!	#NULL!	#NULL!	#NULL!	#NULL!	#NULL!
1.00480	1.00180	1.0000	1.0037	1.0669	1.0739	1.0013	1.0000	1.0045	1.0512
1.00410	1.00160	1.0014	1.0031	1.1138	1.1885	1.0039	1.0008	1.0091	1.0555
1.00350	1.00100	1.0008	1.0024	1.0546	1.0641	1.0028	1.0006	1.0043	1.0454
1.00770	1.00370	1.0026	1.0066	1.1266	1.1314	1.0069	1.0012	1.0061	1.0377
1.00340	1.00230	1.0015	1.0035	1.0464	1.0438	1.0020	1.0006	1.0021	1.0166
1.00530	1.00210	1.0017	1.0051	1.0493	1.0495	1.0024	1.0007	1.0032	1.0255
1.00710	1.00220	1.0017	1.0041	1.0518	1.0648	1.0021	1.0000	1.0059	1.0287
1.00590	1.00250	1.0018	1.0051	1.0701	1.0403	1.0027	1.0000	1.0031	1.0181
1.00550	1.00230	1.0018	1.0048	1.0688	1.1568	1.0032	1.0000	1.0046	1.0212
1.01030	1.00410	1.0025	1.0079	1.1415	1.1595	1.0121	1.0045	1.0086	1.0496

LPC18_11	LPC18_12	LPC18_21	LPC18_22	PEO20_181	PEO20:182	PEO20_201
1.0066	1.0045	1.0038	1.0026	1.7813	1.4027	1.0283
1.0142	1.0081	1.0071	1.0086	1.7857	1.3134	1.0000
1.0215	1.0303	1.0213	1.0464	1.9614	1.4009	1.0373
1.0094	1.0058	1.0056	1.0031	2.1118	1.3885	1.0359
1.0111	1.0094	1.0067	1.0116	2.2396	1.6866	1.0482
1.0084	1.0041	1.0045	1.0035	2.0295	1.4837	1.0341
#NULL!	#NULL!	#NULL!	#NULL!	#NULL!	#NULL!	#NULL!
1.0107	1.0076	1.0070	1.0065	2.5276	1.9737	1.0635
1.0082	1.0018	1.0059	1.0013	2.9215	2.2374	1.0561
1.0336	1.0096	1.0151	1.0062	2.7569	1.8395	1.0530
#NULL!	#NULL!	#NULL!	#NULL!	#NULL!	#NULL!	#NULL!
1.0195	1.0201	1.0446	1.0670	2.1152	1.5716	1.0435
#NULL!	#NULL!	#NULL!	#NULL!	#NULL!	#NULL!	#NULL!
#NULL!	#NULL!	#NULL!	#NULL!	#NULL!	#NULL!	#NULL!
1.0134	1.0102	1.0074	1.0045	1.8212	1.3682	1.0000
1.0221	1.0381	1.0108	1.0121	2.0443	1.3866	1.0402
1.0109	1.0066	1.0074	1.0073	1.9596	1.5250	1.0325
1.0082	1.0124	1.0083	1.0200	1.6630	1.3159	1.0000
1.0057	1.0039	1.0035	1.0026	1.8624	1.5350	1.0351
1.0096	1.0050	1.0047	1.0029	1.6132	1.2696	1.0272
1.0107	1.0106	1.0077	1.0061	1.8225	1.4710	1.0427
1.2391	1.0699	1.1316	1.0559	11.6010	5.9150	1.2305
1.0103	1.0075	1.0059	1.0056	2.0158	1.5075	1.0312
1.0102	1.0050	1.0037	1.0037	1.8264	1.3925	1.0276
1.0107	1.0051	1.0045	1.0035	2.5114	1.6919	1.0505
1.0098	1.0052	1.0063	1.0039	2.1115	1.5745	1.0465
1.0190	1.0135	1.0078	1.0081	1.5780	1.3251	1.0000
1.0092	1.0088	1.0054	1.0060	2.2829	1.9234	1.0476
#NULL!	#NULL!	#NULL!	#NULL!	#NULL!	#NULL!	#NULL!
#NULL!	#NULL!	#NULL!	#NULL!	#NULL!	#NULL!	#NULL!
1.0109	1.0087	1.0050	1.0038	3.0676	1.9652	1.0844
1.0085	1.0057	1.0042	1.0031	2.1946	1.7046	1.0384
1.0109	1.0053	1.0062	1.0041	1.9589	1.5182	1.0364
1.0086	1.0078	1.0063	1.0065	2.1093	1.5316	1.0361
1.0293	1.0187	1.0248	1.0120	1.5052	1.2313	1.0234
1.0097	1.0105	1.0080	1.0106	2.2859	1.7436	1.0338
1.0078	1.0058	1.0041	1.0025	2.7547	1.7870	1.0438
1.0083	1.0039	1.0045	1.0010	2.2838	1.5818	1.0488
1.0124	1.0063	1.0067	1.0033	2.0058	1.4475	1.0399
#NULL!	#NULL!	#NULL!	#NULL!	#NULL!	#NULL!	#NULL!
1.0162	1.0155	1.0080	1.0047	2.0832	1.4363	1.0000
1.0173	1.0188	1.0073	1.0066	2.4369	1.6417	1.0549
1.0335	1.0154	1.0182	1.0109	3.0491	1.7064	1.0417
1.0130	1.0103	1.0100	1.0296	1.9513	1.4298	1.0302
1.0120	1.0092	1.0064	1.0031	1.7011	1.2383	1.0000
1.0136	1.0095	1.0107	1.0061	1.9337	1.4988	1.0477

1.0086	1.0078	1.0042	1.0030	2.0499	1.6710	1.0377
1.0125	1.0083	1.0049	1.0075	2.3811	1.4543	1.0375
1.0194	1.0261	1.0131	1.0163	2.1151	1.3811	1.0585
1.0192	1.0211	1.0151	1.0290	2.6857	1.5596	1.0344
1.0040	1.0034	1.0019	1.0018	1.9850	1.3829	1.0300
1.0079	1.0080	1.0031	1.0045	2.1038	1.5402	1.0324
1.0549	1.1955	1.0231	1.0744	2.1280	1.4836	1.0397
1.0079	1.0049	1.0040	1.0032	2.9109	2.0035	1.0497
1.0087	1.0036	1.0050	1.0028	2.2683	1.5516	1.0380
1.0123	1.0086	1.0060	1.0038	2.2404	1.7211	1.0444
1.0086	1.0062	1.0056	1.0034	1.5172	1.3047	1.0000
#NULL!	#NULL!	#NULL!	#NULL!	#NULL!	#NULL!	#NULL!
1.0083	1.0071	1.0088	1.0034	1.6871	1.5204	1.0307
1.0111	1.0164	1.0232	1.0609	1.8793	1.5093	1.0375
1.0091	1.0075	1.0050	1.0032	2.0223	1.5691	1.0561
1.0104	1.0129	1.0075	1.0126	1.8295	1.4424	1.0107
1.0055	1.0043	1.0038	1.0030	1.7820	1.3871	1.0379
1.0042	1.0047	1.0031	1.0029	1.5792	1.2131	1.0194
1.0073	1.0080	1.0033	1.0034	2.2110	1.4037	1.0546
1.0052	1.0031	1.0027	1.0018	1.9018	1.3576	1.0332
1.0084	1.0176	1.0210	1.0825	1.8877	1.4042	1.0373
1.0212	1.0216	1.0500	1.0497	1.7660	1.2821	1.0254

PEO20_204	PC14_014	PC14_016	PC14_016	PC14_018	PC14_018	PC14_018	PC14_116	PC15_016
1.2785	1.3114	8.0024	1.2355	1.2003	1.4899	1.0488	1.0644	2.4378
1.1007	1.4104	9.2967	1.1581	1.2048	1.4028	1.0446	1.0583	2.8377
1.2505	1.2086	6.2696	1.1770	1.1389	1.4887	1.0426	1.0441	2.0765
1.2312	1.1175	5.1582	1.0996	1.1040	1.3714	1.0278	1.0213	1.9267
1.3414	1.4612	9.1087	1.2063	1.2261	1.4721	1.0598	1.0641	2.8607
1.2190	1.2173	7.1221	1.1383	1.1718	1.3553	1.0503	1.0321	2.6162
#NULL!	#NULL!	#NULL!	#NULL!	#NULL!	#NULL!	#NULL!	#NULL!	#NULL!
1.4416	1.1948	6.6533	1.1167	1.1539	1.3275	1.0426	1.0352	2.4253
1.2739	1.2420	6.9887	1.1635	1.1347	1.3602	1.0466	1.0625	2.5441
1.4758	1.1654	6.3575	1.0976	1.1418	1.4912	1.0511	1.0450	2.4357
#NULL!	#NULL!	#NULL!	#NULL!	#NULL!	#NULL!	#NULL!	#NULL!	#NULL!
1.2835	1.5981	9.6563	1.2218	1.2484	1.6629	1.0875	1.0445	2.0803
#NULL!	#NULL!	#NULL!	#NULL!	#NULL!	#NULL!	#NULL!	#NULL!	#NULL!
#NULL!	#NULL!	#NULL!	#NULL!	#NULL!	#NULL!	#NULL!	#NULL!	#NULL!
1.2411	1.3317	7.5781	1.1834	1.1944	1.5282	1.0667	1.0490	2.1945
1.2341	1.3269	8.7268	1.1345	1.1775	1.4177	1.0454	1.0554	2.5253
1.2449	1.5014	9.2529	1.2059	1.3373	1.7318	1.0926	1.0641	2.3175
1.2297	1.4965	8.1092	1.2688	1.2294	1.7302	1.1282	1.0730	2.3957
1.2528	1.2727	7.2610	1.1284	1.2210	1.4205	1.0654	1.0371	2.1342
1.2466	1.2258	6.5463	1.1330	1.2033	1.4639	1.0815	1.0438	2.0779
1.2882	1.1899	6.0403	1.1138	1.1335	1.3819	1.0545	1.0285	1.9199
1.8993	1.1466	4.6781	1.0962	1.0856	1.3097	1.0854	1.0225	1.6612
1.2200	1.1640	6.6566	1.1070	1.1458	1.3409	1.0324	1.0292	2.1561
1.1963	1.3466	8.0067	1.1785	1.2054	1.4715	1.0505	1.0614	2.5400
1.3070	1.2312	6.7713	1.1451	1.1572	1.4642	1.0443	1.0434	2.3066
1.2769	1.1784	7.3704	1.1014	1.1825	1.3752	1.0308	1.0467	2.5926
1.2049	1.1988	6.2664	1.1557	1.1373	1.4113	1.0588	1.0343	2.0983
1.5504	1.2348	5.9624	1.1755	1.1636	1.4961	1.1001	1.0274	1.8885
#NULL!	#NULL!	#NULL!	#NULL!	#NULL!	#NULL!	#NULL!	#NULL!	#NULL!
#NULL!	#NULL!	#NULL!	#NULL!	#NULL!	#NULL!	#NULL!	#NULL!	#NULL!
1.3068	1.1826	7.4376	1.1146	1.1448	1.2435	1.0168	1.0332	2.6040
1.3214	1.4336	8.6677	1.1617	1.2408	1.4680	1.0569	1.0697	2.4254
1.2608	1.3128	7.5212	1.1313	1.1997	1.3818	1.0526	1.0493	2.2098
1.3284	1.3247	7.0863	1.1757	1.1383	1.7505	1.1039	1.0298	2.2630
1.0717	1.4550	7.9086	1.1718	1.2381	1.5681	1.0747	1.0351	2.4788
1.3148	1.1331	5.7874	1.0777	1.1867	1.3783	1.0437	1.0153	1.8871
1.2423	1.3196	8.2101	1.1547	1.1509	1.3836	1.0389	1.0619	2.5730
1.1459	1.2024	6.4720	1.1416	1.1275	1.3653	1.0355	1.0218	2.3422
1.3034	1.1371	5.5213	1.0854	1.1349	1.4062	1.0396	1.0157	2.0714
#NULL!	#NULL!	#NULL!	#NULL!	#NULL!	#NULL!	#NULL!	#NULL!	#NULL!
1.2076	1.1570	6.3866	1.0963	1.1700	1.2707	1.0310	1.0390	2.3985
1.3661	1.2093	6.7345	1.1424	1.1930	1.5189	1.0736	1.0199	2.4181
1.2365	1.1464	6.1345	1.1002	1.1731	1.4102	1.0383	1.0381	2.3721
1.2497	1.2210	6.6083	1.1645	1.1706	1.3644	1.0457	1.0568	2.2634
1.1928	1.3377	7.8086	1.1614	1.1333	1.3729	1.0468	1.0602	2.4202
1.2231	1.5673	10.8320	1.1676	1.3299	1.5362	1.0689	1.0703	2.7117

1.3340	1.3031	6.9815	1.1584	1.2324	1.5364	1.1124	1.0536	2.2429
1.4434	1.1035	5.5492	1.0614	1.0817	1.2736	1.0019	1.0000	2.3431
1.2700	1.1372	6.7101	1.0616	1.1266	1.2163	1.0170	1.0151	2.5106
1.2870	1.2168	7.7872	1.1543	1.1323	1.3893	1.0270	1.0312	2.5491
1.1580	1.3902	10.1100	1.1304	1.1909	1.3149	1.0176	1.0387	2.9374
1.2649	1.2142	7.5889	1.1078	1.1591	1.3081	1.0258	1.0322	2.4777
1.2930	1.3521	8.6635	1.1039	1.2039	1.3380	1.0363	1.0310	2.5439
1.2537	1.3232	8.5949	1.1373	1.1813	1.3958	1.0382	1.0536	2.5030
1.1678	1.2319	7.5822	1.1517	1.1347	1.3285	1.0316	1.0513	2.6901
1.2401	1.2934	7.6622	1.0801	1.1269	1.2884	1.0361	1.0121	2.1068
1.1452	1.3186	8.2478	1.0768	1.1089	1.2794	1.0345	1.0000	1.5315
#NULL!	#NULL!	#NULL!	#NULL!	#NULL!	#NULL!	#NULL!	#NULL!	#NULL!
1.1864	1.2645	7.1670	1.0996	1.1535	1.3557	1.0636	1.0000	2.1248
1.2773	1.1520	5.8730	1.0568	1.1915	1.3167	1.0460	1.0160	1.8286
1.3466	1.1616	5.8171	1.0826	1.1523	1.3607	1.0503	1.0261	2.1303
1.3108	1.3231	8.1519	1.1877	1.2052	1.4431	1.0548	1.0627	2.7126
1.3274	1.3881	8.7742	1.1598	1.2022	1.4056	1.0622	1.0618	2.9085
1.2307	1.1947	5.7728	1.0820	1.1837	1.3744	1.0386	1.0127	1.9566
1.3867	1.3071	7.9886	1.1578	1.2224	1.3734	1.0499	1.0486	2.1789
1.2230	1.1374	6.4138	1.0696	1.1488	1.2487	1.0179	1.0219	2.1694
1.2274	1.2455	7.9841	1.1284	1.1258	1.3101	1.0257	1.0393	2.1572
1.1822	1.1850	7.0706	1.1088	1.1356	1.2581	1.0233	1.0250	2.2729

PC15_018	PC16.0_80	PC16_016	PC16_016	PC16_017	PC16_017	PC16_017	PC16_018	PC16_018	PC16_018
1.0595	1.0082	48.885	7.7042	1.9855	1.2372	1.0813	2.1934	7.7223	2.6777
1.0522	1.0240	57.041	5.3109	2.2875	1.1761	1.0945	2.2731	6.1273	2.2950
1.0438	1.0107	49.403	7.1459	1.7507	1.2270	1.0515	2.1388	9.4580	3.0580
1.0379	1.0134	53.345	6.0221	1.7544	1.2176	1.0564	2.1729	9.9090	3.0143
1.0556	1.0133	51.186	5.9460	2.1962	1.2221	1.1224	2.2426	7.4869	2.8710
1.0536	1.0140	54.169	5.7297	2.2164	1.1949	1.0905	2.3269	7.8210	3.0617
#NULL!	#NULL!	#NULL!	#NULL!	#NULL!	#NULL!	#NULL!	#NULL!	#NULL!	#NULL!
1.0396	1.0084	51.519	5.5466	2.0591	1.2417	1.1166	2.2160	7.5640	3.1891
1.0429	1.0095	51.750	6.6218	2.2373	1.2855	1.1101	1.9939	7.9140	3.2189
1.0629	1.0124	48.619	5.7696	2.0978	1.2469	1.1083	2.1169	10.4160	3.6057
#NULL!	#NULL!	#NULL!	#NULL!	#NULL!	#NULL!	#NULL!	#NULL!	#NULL!	#NULL!
1.0444	1.0097	49.130	5.8295	1.8535	1.2484	1.0681	2.2053	8.2589	3.3029
#NULL!	#NULL!	#NULL!	#NULL!	#NULL!	#NULL!	#NULL!	#NULL!	#NULL!	#NULL!
#NULL!	#NULL!	#NULL!	#NULL!	#NULL!	#NULL!	#NULL!	#NULL!	#NULL!	#NULL!
1.0553	1.0117	50.918	6.3589	1.8074	1.2205	1.0428	2.2107	8.4952	3.0658
1.0300	1.0193	53.449	5.6529	2.0611	1.2450	1.0958	2.2078	7.5589	2.7829
1.0632	1.0097	45.902	5.8156	1.9223	1.2438	1.0924	2.7527	9.1066	3.0771
1.0669	1.0132	49.084	6.5208	1.9483	1.2188	1.0675	2.4157	8.0977	3.2947
1.0463	1.0083	54.404	4.8867	1.9174	1.1877	1.0804	2.5513	7.2798	3.1283
1.0599	1.0075	53.511	5.1599	1.9113	1.1990	1.0626	2.6762	7.5204	3.4948
1.0357	1.0079	54.477	5.7911	1.8139	1.1877	1.0646	2.2317	7.6032	3.2506
1.0672	1.0029	39.354	4.2321	1.5013	1.1669	1.0412	1.7806	7.8500	3.5088
1.0303	1.0148	58.137	5.5278	1.9659	1.1772	1.0909	2.3304	7.6102	2.5729
1.0489	1.0199	51.554	5.9382	2.1918	1.2785	1.0932	2.4804	7.6361	2.5119
1.0356	1.0107	51.754	6.2662	2.0343	1.2531	1.0790	2.0932	8.5049	3.1274
1.0439	1.0142	51.738	5.9092	2.1958	1.2334	1.1251	2.4005	8.2944	2.9465
1.0299	1.0177	54.649	6.3293	1.8101	1.1609	1.0636	2.1874	8.0397	3.0420
1.0583	1.0144	51.829	5.6079	1.7076	1.1570	1.0414	2.4281	7.6883	3.5387
#NULL!	#NULL!	#NULL!	#NULL!	#NULL!	#NULL!	#NULL!	#NULL!	#NULL!	#NULL!
#NULL!	#NULL!	#NULL!	#NULL!	#NULL!	#NULL!	#NULL!	#NULL!	#NULL!	#NULL!
1.0334	1.0228	55.129	6.3215	2.3906	1.2207	1.1415	2.0731	7.0003	2.2627
1.0424	1.0000	52.695	4.9408	1.9525	1.2205	1.0667	2.3724	6.8771	3.0618
1.0479	1.0000	54.742	5.1649	1.9047	1.1614	1.0725	2.3422	7.0456	2.8325
1.0786	1.0265	50.919	5.5008	1.7840	1.1432	1.0381	2.1136	8.8839	3.8520
1.0531	1.0397	58.213	4.5803	2.0270	1.1466	1.0757	2.6497	7.8334	2.8060
1.0336	1.0085	55.715	4.3710	1.7021	1.1222	1.0505	2.9194	8.2610	3.1633
1.0452	1.0253	52.102	6.2907	1.9462	1.2126	1.0941	1.9486	7.8012	2.9164
1.0399	1.0238	56.628	5.7840	1.9831	1.1860	1.0784	2.1570	7.5805	2.6113
1.0588	1.0205	55.175	4.7552	1.8888	1.1737	1.0530	2.3717	8.6621	3.3473
#NULL!	#NULL!	#NULL!	#NULL!	#NULL!	#NULL!	#NULL!	#NULL!	#NULL!	#NULL!
1.0403	1.0240	55.712	5.3589	2.3152	1.2651	1.0777	2.5101	8.1073	2.6018
1.0621	1.0218	51.893	5.9604	2.1078	1.2583	1.0737	2.5636	9.9045	2.9771
1.0486	1.0000	54.019	5.4437	2.1309	1.2318	1.0778	2.5898	9.2569	2.8299
1.0375	1.0181	56.126	6.5480	2.0550	1.2357	1.0520	2.2337	7.8956	2.6533
1.0185	1.0000	58.576	6.0900	1.9532	1.1992	1.0693	2.0206	5.9551	2.4258
1.0470	1.0196	51.229	5.2048	2.0651	1.2040	1.1105	2.3895	6.8384	3.1415

1.0576	1.0105	50.081	5.0511	2.0153	1.1955	1.0818	2.6217	8.3197	3.7549
1.0452	1.0122	58.531	5.7541	2.1557	1.1974	1.0825	1.8518	7.5823	2.4272
1.0337	1.0292	60.011	5.1556	2.1547	1.2057	1.1095	1.9886	7.0236	2.4413
1.0497	1.0160	51.301	7.4375	1.9187	1.2383	1.0914	1.7824	7.9540	2.8424
1.0216	1.0390	59.114	5.2558	2.2316	1.1932	1.1529	1.9698	5.9357	1.9478
1.0580	1.0274	56.256	5.8364	2.1821	1.2524	1.1061	2.2032	7.5956	2.5664
1.0267	1.0346	52.582	4.4909	2.1364	1.1898	1.1090	2.2260	7.8575	2.4221
1.0301	1.0259	52.896	5.4419	2.0092	1.2112	1.1014	1.9394	7.5236	2.7970
1.0506	1.0278	55.328	6.5137	2.2545	1.2325	1.1035	1.8357	7.3163	2.4240
1.0295	1.0421	60.945	4.1937	1.7006	1.1450	1.0397	2.0771	5.6661	2.5996
1.0193	1.0181	62.254	3.9628	1.3728	1.0679	1.0138	1.8586	5.5473	2.7253
#NULL!	#NULL!	#NULL!	#NULL!	#NULL!	#NULL!	#NULL!	#NULL!	#NULL!	#NULL!
1.0569	1.0225	61.669	3.8792	1.7915	1.1210	1.0632	2.3377	5.9412	3.3614
1.0322	1.0351	58.954	3.5184	1.8485	1.1400	1.0578	2.8579	7.5802	3.3778
1.0336	1.0202	57.810	4.4023	2.1034	1.1840	1.0782	2.6403	8.0584	3.0729
1.0595	1.0318	52.101	6.6631	2.2686	1.2757	1.1278	2.3231	7.3588	2.7493
1.0193	1.0212	57.138	5.1843	2.4331	1.2483	1.1658	2.3699	6.0222	2.4847
1.0427	1.0333	60.680	4.1740	1.8413	1.1515	1.0508	2.9814	7.4642	2.3662
1.0385	1.0226	56.801	5.4482	1.9405	1.2019	1.0971	2.5390	6.8374	2.4575
1.0420	1.0317	59.512	5.7439	1.9693	1.1646	1.0589	2.4527	7.1905	2.2864
1.0426	1.0000	56.833	6.1902	1.8246	1.2119	1.0598	1.9665	6.8047	2.6607
1.0269	1.0387	55.803	6.2450	1.9668	1.2032	1.1133	2.0776	6.7895	2.6621

PC16_018	PC16_018	PC16_020	PC16_020	PC16_022	PC16_116	PC16_118	PC16_118	PC16_118	PC18_018
1.0836	1.0754	1.1612	1.0223	1.0000	1.2358	1.2360	1.3952	1.0809	1.7187
1.0519	1.0547	1.0764	1.0122	1.0084	1.0969	1.1329	1.1505	1.0449	1.4601
1.0710	1.1171	1.1844	1.0173	1.0209	1.1753	1.1860	1.3250	1.0615	1.8769
1.1173	1.0761	1.1583	1.0297	1.0175	1.1199	1.1493	1.2914	1.0574	1.8649
1.1195	1.0553	1.1274	1.0323	1.0162	1.1116	1.1701	1.2131	1.0486	1.7021
1.1074	1.0656	1.1549	1.0313	1.0124	1.0949	1.1642	1.1930	1.0471	1.7315
#NULL!	#NULL!	#NULL!	#NULL!	#NULL!	#NULL!	#NULL!	#NULL!	#NULL!	#NULL!
1.1472	1.0668	1.1125	1.0161	1.0136	1.0908	1.1490	1.1862	1.0558	1.7489
1.1175	1.0700	1.0955	1.0122	1.0039	1.1539	1.1789	1.2719	1.0783	1.6871
1.1025	1.0828	1.2615	1.0159	1.0086	1.0833	1.1829	1.2224	1.0637	1.9493
#NULL!	#NULL!	#NULL!	#NULL!	#NULL!	#NULL!	#NULL!	#NULL!	#NULL!	#NULL!
1.0885	1.0851	1.1447	1.0376	1.0130	1.1175	1.1811	1.2492	1.0742	1.8543
#NULL!	#NULL!	#NULL!	#NULL!	#NULL!	#NULL!	#NULL!	#NULL!	#NULL!	#NULL!
#NULL!	#NULL!	#NULL!	#NULL!	#NULL!	#NULL!	#NULL!	#NULL!	#NULL!	#NULL!
1.0877	1.1273	1.1883	1.0206	1.0157	1.1587	1.1881	1.3312	1.0715	1.7014
1.0601	1.0767	1.1283	1.0067	1.0073	1.0708	1.1526	1.1657	1.0472	1.6821
1.1328	1.0788	1.2105	1.0350	1.0227	1.1120	1.2438	1.2976	1.0852	1.9768
1.0737	1.1710	1.2490	1.0455	1.0202	1.1856	1.2144	1.3608	1.0990	1.7409
1.0833	1.1241	1.1510	1.0431	1.0152	1.0884	1.1400	1.1997	1.0685	1.6351
1.1387	1.1088	1.1793	1.0261	1.0116	1.0986	1.1804	1.2795	1.1068	1.7340
1.1140	1.0821	1.1678	1.0245	1.0213	1.1244	1.1682	1.2065	1.0908	1.6611
1.1007	1.1129	1.2012	1.0220	1.0115	1.0896	1.1309	1.1919	1.1473	2.0765
1.0689	1.0406	1.1061	1.0231	1.0094	1.0903	1.1459	1.1904	1.0554	1.7679
1.0815	1.0896	1.0983	1.0146	1.0068	1.1162	1.1918	1.2331	1.0538	1.8564
1.1138	1.0626	1.1503	1.0154	1.0128	1.0926	1.1747	1.2191	1.0715	1.8184
1.0582	1.0616	1.1830	1.0244	1.0128	1.0758	1.1961	1.1560	1.0460	1.8784
1.0294	1.0778	1.1696	1.0166	1.0046	1.1566	1.1670	1.3070	1.1076	1.6553
1.1254	1.0921	1.1588	1.0535	1.0179	1.1673	1.1633	1.2777	1.1232	1.6077
#NULL!	#NULL!	#NULL!	#NULL!	#NULL!	#NULL!	#NULL!	#NULL!	#NULL!	#NULL!
#NULL!	#NULL!	#NULL!	#NULL!	#NULL!	#NULL!	#NULL!	#NULL!	#NULL!	#NULL!
1.0592	1.0235	1.0658	1.0085	1.0067	1.0803	1.1647	1.1427	1.0271	1.6937
1.0403	1.1429	1.1610	1.0131	1.0071	1.0881	1.1512	1.1017	1.0611	1.7168
1.0555	1.1066	1.1236	1.0112	1.0067	1.0959	1.1601	1.2002	1.0449	1.6586
1.1201	1.1101	1.1800	1.0261	1.0113	1.1582	1.1438	1.3829	1.1343	1.6423
1.0620	1.0308	1.0601	1.0140	1.0056	1.0767	1.1443	1.1986	1.0687	1.7106
1.1145	1.0635	1.1497	1.0107	1.0042	1.0529	1.1651	1.1568	1.0519	1.9883
1.0275	1.0515	1.0800	1.0082	1.0067	1.1027	1.1301	1.1768	1.0374	1.6776
1.0597	1.0486	1.0487	1.0027	1.0001	1.1018	1.1273	1.2121	1.0643	1.6212
1.1175	1.0840	1.1682	1.0125	1.0038	1.0689	1.1373	1.2127	1.0626	1.7230
#NULL!	#NULL!	#NULL!	#NULL!	#NULL!	#NULL!	#NULL!	#NULL!	#NULL!	#NULL!
1.0648	1.0570	1.1222	1.0085	1.0000	1.0567	1.1958	1.1469	1.0380	1.9341
1.0651	1.0329	1.1782	1.0068	1.0050	1.1062	1.2389	1.3199	1.0560	1.9829
1.0669	1.0406	1.1557	1.0089	1.0002	1.0766	1.1974	1.2308	1.0468	1.9494
1.0702	1.1093	1.1630	1.0173	1.0002	1.1497	1.2088	1.1466	1.0719	1.6974
1.0592	1.0830	1.1244	1.0061	1.0094	1.1299	1.1178	1.1556	1.0432	1.4738
1.0777	1.1519	1.1950	1.0278	1.0094	1.0501	1.1544	1.0573	1.0458	1.7133

1.1096	1.1345	1.1991	1.0627	1.0245	1.0966	1.1647	1.2356	1.0674	2.0065
1.0526	1.0473	1.1438	1.0041	1.0023	1.0640	1.1205	1.1306	1.0180	2.0012
1.0701	1.0342	1.1256	1.0287	1.0082	1.0325	1.1314	1.1050	1.0270	1.6496
1.0510	1.0943	1.1112	1.0114	1.0019	1.1190	1.1631	1.2393	1.0471	1.6681
1.0370	1.0088	1.0708	1.0079	1.0050	1.0544	1.1066	1.1041	1.0186	1.4924
1.0687	1.0644	1.1388	1.0189	1.0059	1.0797	1.1818	1.1254	1.0463	1.7629
1.0681	1.0535	1.0872	1.0074	1.0068	1.0502	1.1261	1.1098	1.0216	1.8189
1.1161	1.0317	1.0840	1.0091	1.0004	1.0982	1.1380	1.1701	1.0328	1.6713
1.0660	1.0214	1.0750	1.0080	1.0040	1.1362	1.1603	1.1681	1.0471	1.5954
1.0737	1.0819	1.0536	1.0106	1.0051	1.0541	1.0843	1.0881	1.0186	1.3904
1.0581	1.0417	1.0966	1.0053	1.0003	1.0211	1.0580	1.0607	1.0069	1.3814
#NULL!	#NULL!	#NULL!	#NULL!	#NULL!	#NULL!	#NULL!	#NULL!	#NULL!	#NULL!
1.0549	1.0733	1.0686	1.0046	1.0039	1.0314	1.0900	1.1017	1.0260	1.4722
1.0820	1.0819	1.1309	1.0124	1.0033	1.0356	1.1133	1.1668	1.0534	1.8033
1.0798	1.0375	1.1526	1.0102	1.0000	1.0734	1.1465	1.2267	1.0486	1.8083
1.1065	1.0664	1.1509	1.0143	1.0134	1.1557	1.2154	1.2520	1.0591	1.6119
1.0806	1.0571	1.1736	1.0325	1.0210	1.1100	1.1409	1.1866	1.0531	1.4829
1.0969	1.1041	1.1658	1.0147	1.0070	1.0718	1.1381	1.2101	1.0363	1.6990
1.0632	1.1355	1.2301	1.0368	1.0167	1.1114	1.1659	1.2229	1.0407	1.6258
1.0577	1.0500	1.1246	1.0056	1.0021	1.0772	1.1615	1.1615	1.0430	1.6655
1.0863	1.0670	1.1117	1.0185	1.0117	1.1336	1.1340	1.1970	1.0311	1.4779
1.1220	1.0469	1.1130	1.0138	1.0058	1.0978	1.1764	1.1249	1.0000	1.5791

PC18_018	PC18_020	PC18_118	PC18_118	PC18_120	PC18_218	PC16_0e1(	PC16_0p1(	PC160_70a	PC160_90a
1.4434	1.0712	2.1266	1.4535	1.0277	1.1418	1.1540	1.0718	1.0492	1.3562
1.3312	1.0302	1.5277	1.2798	1.0113	1.1027	1.1512	1.0510	1.1303	1.9692
1.4827	1.0593	2.5975	1.5310	1.0290	1.1221	1.1783	1.0291	1.0531	1.4866
1.4504	1.0540	2.7155	1.4678	1.0219	1.1311	1.1129	1.0298	1.0870	1.7251
1.5207	1.0553	2.0042	1.4424	1.0177	1.1270	1.1466	1.0264	1.0654	1.5210
1.5620	1.0619	1.9947	1.4742	1.0190	1.1615	1.1367	1.0949	1.0893	1.7050
#NULL!	#NULL!	#NULL!	#NULL!	#NULL!	#NULL!	#NULL!	#NULL!	#NULL!	#NULL!
1.5963	1.0460	2.2129	1.5519	1.0121	1.2148	1.1811	1.0975	1.0684	1.3954
1.5477	1.0354	2.0166	1.4888	1.0183	1.2107	1.1602	1.0595	1.0736	1.6435
1.6251	1.0830	2.6140	1.5969	1.0294	1.2125	1.1909	1.0407	1.0684	1.8078
#NULL!	#NULL!	#NULL!	#NULL!	#NULL!	#NULL!	#NULL!	#NULL!	#NULL!	#NULL!
1.6534	1.0688	2.2031	1.5895	1.0261	1.2445	1.1906	1.0891	1.0533	1.5136
#NULL!	#NULL!	#NULL!	#NULL!	#NULL!	#NULL!	#NULL!	#NULL!	#NULL!	#NULL!
#NULL!	#NULL!	#NULL!	#NULL!	#NULL!	#NULL!	#NULL!	#NULL!	#NULL!	#NULL!
1.5368	1.0795	2.5157	1.6465	1.0334	1.1695	1.2026	1.0475	1.0532	1.5017
1.4491	1.0378	1.8234	1.3911	1.0198	1.1346	1.1838	1.0360	1.0801	1.7552
1.6967	1.0931	2.4120	1.6517	1.0424	1.2785	1.1553	1.1188	1.0476	1.5557
1.6336	1.0840	2.4608	1.8604	1.0458	1.2706	1.1745	1.0914	1.0537	1.4175
1.6526	1.0509	2.0346	1.6626	1.0243	1.3538	1.1475	1.0653	1.0530	1.4230
1.8342	1.0870	2.3868	1.8795	1.0303	1.3171	1.1518	1.0933	1.0401	1.3744
1.6048	1.0699	2.3420	1.6882	1.0382	1.3206	1.1659	1.1085	1.0532	1.4864
1.8515	1.0693	3.2810	2.2267	1.0433	1.5178	1.1173	1.0718	1.0283	1.3430
1.3717	1.0363	1.8388	1.3729	1.0137	1.1484	1.1461	1.0028	1.1152	2.0891
1.4813	1.0508	2.0664	1.3912	1.0142	1.1220	1.1686	1.0543	1.0847	1.6771
1.4811	1.0416	2.1602	1.4765	1.0243	1.1425	1.1629	1.0559	1.0714	1.5855
1.5264	1.0660	2.0072	1.4073	1.0192	1.1721	1.1300	1.0675	1.0571	1.5662
1.3978	1.0473	2.3837	1.6118	1.0248	1.1646	1.1510	1.0233	1.1022	1.8549
1.5644	1.0488	2.6338	1.9766	1.0257	1.2793	1.1639	1.1021	1.0733	1.6533
#NULL!	#NULL!	#NULL!	#NULL!	#NULL!	#NULL!	#NULL!	#NULL!	#NULL!	#NULL!
#NULL!	#NULL!	#NULL!	#NULL!	#NULL!	#NULL!	#NULL!	#NULL!	#NULL!	#NULL!
1.3604	1.0316	1.6172	1.2106	1.0034	1.0503	1.1779	1.0183	1.1634	2.1541
1.5424	1.0565	1.8874	1.5619	1.0130	1.2572	1.2356	1.0752	1.0582	1.5873
1.5003	1.0536	1.7694	1.4596	1.0172	1.2304	1.2353	1.0341	1.0605	1.6190
1.6036	1.0463	3.1041	2.1911	1.0431	1.2626	1.1512	1.0000	1.1231	2.2474
1.4542	1.0251	2.0918	1.4504	1.0080	1.1146	1.1651	1.0000	1.1907	2.6267
1.6921	1.0696	2.3103	1.6160	1.0244	1.2028	1.1478	1.0348	1.0880	1.7789
1.4134	1.0270	1.9058	1.3729	1.0012	1.0982	1.1760	1.0405	1.1235	1.9887
1.3828	1.0215	2.0784	1.3842	1.0072	1.0809	1.2086	1.0016	1.2009	2.3274
1.5361	1.0570	2.4942	1.6595	1.0306	1.1747	1.1611	1.0037	1.0746	1.8257
#NULL!	#NULL!	#NULL!	#NULL!	#NULL!	#NULL!	#NULL!	#NULL!	#NULL!	#NULL!
1.5133	1.0417	1.7767	1.2539	1.0091	1.0874	1.1658	1.0222	1.1176	1.8934
1.4830	1.0582	2.5799	1.5186	1.0212	1.1531	1.1813	1.0101	1.1060	2.1875
1.5006	1.0664	2.3980	1.4809	1.0335	1.1979	1.1507	1.0120	1.0933	2.0043
1.4284	1.0483	1.9531	1.3355	1.0236	1.1114	1.1638	1.0256	1.0697	1.7352
1.3184	1.0473	1.4364	1.3337	1.0030	1.0649	1.2346	1.0000	1.1123	1.9056
1.6521	1.0710	1.6483	1.3849	1.0312	1.1743	1.2730	1.1094	1.0687	1.7684

1.8110	1.0937	2.4722	1.9351	1.0295	1.4226	1.2168	1.0999	1.0393	1.5517
1.2959	1.0360	1.7867	1.2366	1.0182	1.0673	1.1193	1.0264	1.1663	1.8883
1.3372	1.0472	1.5977	1.2080	1.0133	1.1053	1.1415	1.0241	1.1479	2.1536
1.3253	1.0442	1.9749	1.3126	1.0075	1.0734	1.1643	1.0446	1.1008	1.8084
1.1646	1.0145	1.5123	1.1472	1.0008	1.0139	1.1618	1.0000	1.2091	2.7392
1.4089	1.0492	1.7677	1.3422	1.0144	1.1057	1.2452	1.0183	1.1265	1.9920
1.4339	1.0412	1.7986	1.2822	1.0134	1.1046	1.1606	1.0461	1.1251	2.2025
1.4323	1.0222	1.9111	1.4422	1.0065	1.1271	1.1804	1.0432	1.1028	1.9541
1.3198	1.0247	1.8759	1.3151	1.0069	1.0912	1.1516	1.0427	1.1581	2.1001
1.3222	1.0171	1.5849	1.3767	1.0107	1.0949	1.1477	1.0136	1.1340	1.9819
1.3070	1.0246	1.6557	1.3047	1.0159	1.0484	1.1206	1.0102	1.1486	2.0877
#NULL!	#NULL!	#NULL!	#NULL!	#NULL!	#NULL!	#NULL!	#NULL!	#NULL!	#NULL!
1.5472	1.0412	1.7411	1.4776	1.0148	1.1719	1.1746	1.0000	1.1429	2.1390
1.7190	1.0635	2.2267	1.7389	1.0274	1.3031	1.2315	1.0194	1.1104	2.4969
1.5699	1.0476	2.4736	1.6493	1.0285	1.2773	1.1692	1.0506	1.1112	2.2007
1.4231	1.0459	1.7906	1.4276	1.0220	1.2153	1.2151	1.0262	1.0805	1.9888
1.2957	1.0619	1.6974	1.3997	1.0202	1.2219	1.1634	1.0558	1.0669	1.8905
1.3681	1.0717	2.4552	1.5570	1.0398	1.0805	1.1782	1.0417	1.1457	2.5712
1.3764	1.0774	1.9307	1.4040	1.0385	1.1124	1.1770	1.0624	1.0952	1.9114
1.3358	1.0387	1.7312	1.2704	1.0114	1.1081	1.2106	1.0259	1.1718	2.4585
1.3105	1.0366	1.8455	1.2539	1.0134	1.1343	1.2144	1.0374	1.1358	1.9749
1.3487	1.0365	1.6909	1.2264	1.0094	1.3874	1.2520	1.0136	1.1260	2.1477

PC180_90:	PE14_018_	PE16_016_	PE16_018_	PE16_018_	PE16_020_	PE16_118_	PE16_118_	PE18_018_	PE18_018_
1.0408	1.0123	1.0434	1.3311	1.0919	1.0382	1.0031	1.0363	1.1557	1.0644
1.0969	1.0139	1.0164	1.2222	1.0742	1.0172	1.0000	1.0147	1.1081	1.0577
1.0624	1.0114	1.0297	1.2762	1.1021	1.0356	1.0000	1.0290	1.1364	1.0617
1.0677	1.0053	1.0176	1.2402	1.0747	1.0308	1.0019	1.0199	1.1499	1.0538
1.0605	1.0079	1.0227	1.2934	1.0924	1.0395	1.0000	1.0236	1.1770	1.0784
1.0929	1.0083	1.0178	1.2622	1.0716	1.0302	1.0013	1.0167	1.1832	1.0726
#NULL!	#NULL!	#NULL!	#NULL!	#NULL!	#NULL!	#NULL!	#NULL!	#NULL!	#NULL!
1.0451	1.0067	1.0195	1.2026	1.0861	1.0272	1.0015	1.0194	1.1634	1.0859
1.0762	1.0090	1.0291	1.2673	1.0847	1.0176	1.0000	1.0256	1.1660	1.0777
1.0899	1.0119	1.0186	1.2990	1.1097	1.0326	1.0029	1.0183	1.1709	1.0744
#NULL!	#NULL!	#NULL!	#NULL!	#NULL!	#NULL!	#NULL!	#NULL!	#NULL!	#NULL!
1.0874	1.0103	1.0195	1.2719	1.0756	1.0202	1.0000	1.0180	1.1630	1.0940
#NULL!	#NULL!	#NULL!	#NULL!	#NULL!	#NULL!	#NULL!	#NULL!	#NULL!	#NULL!
#NULL!	#NULL!	#NULL!	#NULL!	#NULL!	#NULL!	#NULL!	#NULL!	#NULL!	#NULL!
1.0510	1.0170	1.0295	1.2699	1.0836	1.0420	1.0027	1.0269	1.1478	1.0547
1.0895	1.0091	1.0242	1.2732	1.0861	1.0346	1.0036	1.0205	1.1289	1.0542
1.0752	1.0111	1.0195	1.2557	1.0736	1.0383	1.0046	1.0228	1.1571	1.0749
1.0535	1.0142	1.0229	1.2504	1.0972	1.0516	1.0015	1.0302	1.1513	1.0671
1.0561	1.0097	1.0176	1.2279	1.0788	1.0229	1.0011	1.0132	1.1469	1.0797
1.0576	1.0074	1.0150	1.2092	1.0899	1.0331	1.0039	1.0221	1.1520	1.0918
1.0522	1.0094	1.0211	1.2228	1.0861	1.0317	1.0034	1.0209	1.1710	1.0918
1.0550	1.0134	1.0305	1.3812	1.1181	1.0355	1.0104	1.0327	1.3171	1.1588
1.0988	1.0104	1.0221	1.2278	1.0699	1.0257	1.0009	1.0226	1.0989	1.0611
1.0855	1.0082	1.0231	1.2764	1.0810	1.0288	1.0027	1.0260	1.1637	1.0639
1.0624	1.0115	1.0256	1.3020	1.0895	1.0379	1.0033	1.0300	1.1691	1.0628
1.0666	1.0105	1.0173	1.2920	1.0858	1.0365	1.0042	1.0252	1.2115	1.0884
1.0855	1.0092	1.0224	1.2469	1.0741	1.0275	1.0017	1.0191	1.1387	1.0597
1.0635	1.0113	1.0157	1.2232	1.0995	1.0354	1.0027	1.0167	1.1185	1.0792
#NULL!	#NULL!	#NULL!	#NULL!	#NULL!	#NULL!	#NULL!	#NULL!	#NULL!	#NULL!
#NULL!	#NULL!	#NULL!	#NULL!	#NULL!	#NULL!	#NULL!	#NULL!	#NULL!	#NULL!
1.1283	1.0058	1.0226	1.2271	1.0488	1.0108	1.0026	1.0115	1.2035	1.0584
1.0679	1.0128	1.0212	1.2517	1.0846	1.0240	1.0000	1.0173	1.1581	1.0730
1.0722	1.0084	1.0223	1.2354	1.0696	1.0252	1.0031	1.0214	1.1595	1.0670
1.1259	1.0074	1.0171	1.2308	1.0877	1.0278	1.0043	1.0169	1.1253	1.0511
1.2055	1.0130	1.0157	1.1840	1.0529	1.0113	1.0013	1.0043	1.0998	1.0434
1.1301	1.0064	1.0081	1.1786	1.0622	1.0258	1.0041	1.0128	1.2198	1.0932
1.1111	1.0100	1.0325	1.2327	1.0871	1.0171	1.0000	1.0199	1.1476	1.0674
1.1690	1.0055	1.0170	1.1803	1.0617	1.0111	1.0013	1.0172	1.1385	1.0550
1.0978	1.0090	1.0154	1.2591	1.0817	1.0287	1.0031	1.0098	1.1896	1.0730
#NULL!	#NULL!	#NULL!	#NULL!	#NULL!	#NULL!	#NULL!	#NULL!	#NULL!	#NULL!
1.1131	1.0083	1.0143	1.2547	1.0614	1.0206	1.0041	1.0169	1.2240	1.0805
1.1254	1.0047	1.0227	1.2312	1.0613	1.0207	1.0013	1.0232	1.1898	1.0613
1.1254	1.0115	1.0181	1.2375	1.0548	1.0223	1.0000	1.0207	1.1887	1.0607
1.0709	1.0101	1.0249	1.2792	1.0868	1.0362	1.0022	1.0221	1.1578	1.0790
1.0837	1.0087	1.0308	1.2288	1.0625	1.0291	1.0018	1.0149	1.1071	1.0546
1.1305	1.0190	1.0122	1.2134	1.0673	1.0191	1.0000	1.0175	1.1347	1.0719

1.0602	1.0046	1.0119	1.2034	1.0795	1.0289	1.0029	1.0147	1.1641	1.0910
1.0790	1.0092	1.0262	1.3138	1.0638	1.0365	1.0013	1.0218	1.1819	1.0661
1.1090	1.0049	1.0160	1.2792	1.0647	1.0258	1.0000	1.0113	1.1587	1.0532
1.0744	1.0107	1.0312	1.2892	1.0910	1.0482	1.0034	1.0305	1.1703	1.0632
1.1374	1.0097	1.0173	1.2162	1.0427	1.0161	1.0000	1.0135	1.1139	1.0359
1.0992	1.0065	1.0207	1.2353	1.0821	1.0290	1.0041	1.0204	1.1555	1.0811
1.1553	1.0086	1.0145	1.2267	1.0558	1.0224	1.0035	1.0131	1.1483	1.0638
1.1083	1.0140	1.0201	1.2771	1.0746	1.0143	1.0021	1.0232	1.1765	1.0652
1.1102	1.0096	1.0224	1.2591	1.0576	1.0160	1.0043	1.0236	1.1489	1.0528
1.1022	1.0079	1.0126	1.2254	1.0734	1.0147	1.0000	1.0114	1.1463	1.0705
1.1203	1.0058	1.0109	1.1869	1.0595	1.0162	1.0013	1.0076	1.1193	1.0547
#NULL!	#NULL!	#NULL!	#NULL!	#NULL!	#NULL!	#NULL!	#NULL!	#NULL!	#NULL!
1.1434	1.0079	1.0126	1.1750	1.0887	1.0213	1.0015	1.0103	1.1083	1.0714
1.2561	1.0049	1.0072	1.1475	1.0544	1.0168	1.0000	1.0066	1.1330	1.0567
1.1780	1.0044	1.0113	1.1625	1.0444	1.0161	1.0048	1.0159	1.1555	1.0582
1.0907	1.0135	1.0365	1.2492	1.1323	1.0346	1.0064	1.0302	1.1457	1.0698
1.0731	1.0099	1.0230	1.2262	1.0618	1.0358	1.0041	1.0221	1.1399	1.0573
1.2220	1.0055	1.0109	1.1603	1.0399	1.0262	1.0000	1.0172	1.1358	1.0448
1.1005	1.0094	1.0181	1.1950	1.0484	1.0380	1.0037	1.0174	1.1415	1.0498
1.1589	1.0080	1.0242	1.2407	1.0551	1.0184	1.0000	1.0189	1.1627	1.0504
1.1000	1.0141	1.0308	1.2641	1.0747	1.0331	1.0026	1.0260	1.1481	1.0558
1.1146	1.0091	1.0285	1.3033	1.2468	1.0286	1.0039	1.0260	1.1556	1.0629

PE18_020	PE18_118	PE18_118	PE18_120	PG14_016	PG16_016	PG16_016	PG16_018	PG16_018	PG16_018
1.0311	1.5246	1.2121	1.0460	1.0624	1.6903	1.2033	1.1619	3.6526	1.1324
1.0287	1.2810	1.1155	1.0153	1.0750	1.7644	1.1249	1.1796	3.3263	1.1248
1.0329	1.5926	1.1972	1.0410	1.0501	1.8545	1.1922	1.2174	4.1932	1.1237
1.0210	1.5256	1.1635	1.0296	1.0301	1.6800	1.1222	1.1933	4.0581	1.0967
1.0387	1.4375	1.1858	1.0343	1.0977	1.9558	1.1730	1.2880	3.7371	1.1571
1.0308	1.4143	1.1513	1.0228	1.0516	1.7028	1.1350	1.2256	3.3673	1.1357
#NULL!	#NULL!	#NULL!	#NULL!	#NULL!	#NULL!	#NULL!	#NULL!	#NULL!	#NULL!
1.0270	1.4910	1.2131	1.0271	1.0738	1.9060	1.1220	1.3246	3.7154	1.1290
1.0166	1.4849	1.1961	1.0183	1.0618	1.7780	1.1952	1.1987	3.9350	1.1430
1.0435	1.5839	1.1970	1.0361	1.0596	1.7890	1.1631	1.2099	4.3620	1.1420
#NULL!	#NULL!	#NULL!	#NULL!	#NULL!	#NULL!	#NULL!	#NULL!	#NULL!	#NULL!
1.0300	1.4939	1.2037	1.0272	1.0776	1.7071	1.1504	1.1984	3.9088	1.1701
#NULL!	#NULL!	#NULL!	#NULL!	#NULL!	#NULL!	#NULL!	#NULL!	#NULL!	#NULL!
#NULL!	#NULL!	#NULL!	#NULL!	#NULL!	#NULL!	#NULL!	#NULL!	#NULL!	#NULL!
1.0370	1.4775	1.1898	1.0390	1.0610	1.7987	1.1761	1.2073	3.8000	1.1634
1.0202	1.3750	1.1405	1.0293	1.0775	1.8531	1.1662	1.2292	4.0328	1.1449
1.0304	1.4665	1.2064	1.0408	1.1011	2.0000	1.1940	1.3181	4.8195	1.2093
1.0520	1.4630	1.2050	1.0575	1.0843	1.9567	1.2051	1.2964	3.8379	1.2230
1.0244	1.4551	1.2049	1.0320	1.0572	1.7108	1.1041	1.2230	3.7482	1.1716
1.0372	1.4893	1.3042	1.0548	1.0461	1.6661	1.1195	1.2408	3.4483	1.1895
1.0322	1.4693	1.2109	1.0391	1.0383	1.6020	1.1286	1.2105	3.0525	1.1309
1.0417	1.4965	1.2108	1.0429	1.0337	1.5700	1.0805	1.1411	2.9467	1.1532
1.0138	1.3724	1.1399	1.0274	1.0562	1.9665	1.1820	1.2085	3.8640	1.1262
1.0237	1.4476	1.1464	1.0276	1.0664	1.7441	1.1422	1.2461	3.6470	1.1206
1.0247	1.5036	1.1899	1.0340	1.0591	1.8833	1.1770	1.2791	4.0744	1.1422
1.0325	1.4998	1.1956	1.0316	1.0533	1.6936	1.1433	1.2418	3.8105	1.1295
1.0264	1.3737	1.1345	1.0268	1.0524	2.0336	1.1748	1.2791	3.5283	1.1676
1.0301	1.3684	1.2109	1.0372	1.0607	2.0855	1.1950	1.3241	3.8308	1.2777
#NULL!	#NULL!	#NULL!	#NULL!	#NULL!	#NULL!	#NULL!	#NULL!	#NULL!	#NULL!
#NULL!	#NULL!	#NULL!	#NULL!	#NULL!	#NULL!	#NULL!	#NULL!	#NULL!	#NULL!
1.0235	1.3005	1.0861	1.0043	1.0682	1.9252	1.1758	1.2860	3.4707	1.0839
1.0301	1.4560	1.2160	1.0271	1.0908	1.8091	1.1501	1.2539	4.0639	1.1845
1.0284	1.4291	1.1709	1.0227	1.0568	1.7571	1.1483	1.2405	3.7755	1.1604
1.0170	1.3816	1.1880	1.0308	1.0480	1.7759	1.1145	1.2095	3.3362	1.1982
1.0096	1.1828	1.0915	1.0093	1.0678	1.9319	1.1012	1.3051	3.2628	1.1215
1.0329	1.4859	1.1873	1.0334	1.0327	1.6000	1.0666	1.2450	3.4696	1.1276
1.0137	1.4326	1.1815	1.0159	1.0899	2.0606	1.1950	1.2526	4.3563	1.1476
1.0121	1.3944	1.1580	1.0096	1.0426	1.8030	1.1177	1.2189	3.4144	1.0929
1.0296	1.4309	1.1739	1.0336	1.0422	1.8248	1.1017	1.2095	3.6641	1.1513
#NULL!	#NULL!	#NULL!	#NULL!	#NULL!	#NULL!	#NULL!	#NULL!	#NULL!	#NULL!
1.0362	1.4038	1.1132	1.0141	1.0555	1.9360	1.1443	1.3351	4.3474	1.1301
1.0304	1.5113	1.1744	1.0370	1.0401	1.6641	1.1333	1.1965	3.2503	1.1033
1.0298	1.4766	1.1464	1.0273	1.0360	1.6178	1.1106	1.1838	3.3662	1.0966
1.0460	1.3496	1.1405	1.0338	1.0524	1.8715	1.1870	1.2227	3.9337	1.1728
1.0290	1.2643	1.1089	1.0217	1.0849	2.0540	1.2598	1.2152	3.8345	1.2176
1.0442	1.3193	1.1433	1.0294	1.1236	1.9368	1.1799	1.2953	4.4429	1.2230

1.0355	1.4307	1.2459	1.0359	1.0537	1.6605	1.1157	1.2364	3.8951	1.2114
1.0478	1.4489	1.1098	1.0345	1.0293	1.4969	1.0904	1.1097	2.9778	1.0686
1.0240	1.3021	1.0914	1.0165	1.0380	1.5683	1.0926	1.1596	3.0926	1.0827
1.0377	1.4793	1.1813	1.0439	1.0931	2.0479	1.2496	1.2627	4.0114	1.1490
1.0136	1.2188	1.0683	1.0122	1.1226	2.1393	1.1615	1.3057	3.4435	1.0843
1.0290	1.3416	1.1493	1.0321	1.0610	1.8078	1.1534	1.2494	3.7044	1.1253
1.0242	1.3016	1.0934	1.0139	1.0610	1.6541	1.0855	1.2025	3.3084	1.0881
1.0163	1.5496	1.1716	1.0193	1.0790	1.8219	1.1336	1.1991	3.8203	1.1158
1.0180	1.4282	1.1231	1.0127	1.0698	1.9499	1.2366	1.2059	3.8575	1.0982
1.0097	1.3499	1.1646	1.0181	1.0791	2.0658	1.1023	1.2794	3.4468	1.1456
1.0162	1.3122	1.1318	1.0183	1.0665	1.7569	1.0582	1.2112	2.5211	1.0906
#NULL!	#NULL!	#NULL!	#NULL!	#NULL!	#NULL!	#NULL!	#NULL!	#NULL!	#NULL!
1.0179	1.2529	1.1546	1.0129	1.0713	2.0084	1.0867	1.2836	3.4183	1.1981
1.0219	1.3286	1.1338	1.0163	1.0357	1.6829	1.0546	1.2805	3.0539	1.1180
1.0247	1.3809	1.1572	1.0233	1.0291	1.5967	1.0696	1.2533	2.8653	1.0743
1.0460	1.3920	1.1727	1.0366	1.1038	2.2005	1.2858	1.2647	4.1862	1.2117
1.0352	1.3211	1.1400	1.0392	1.1188	2.2223	1.2576	1.3336	4.0257	1.1799
1.0258	1.3124	1.0929	1.0254	1.0359	1.6792	1.0695	1.2559	2.7386	1.0940
1.0355	1.3449	1.1192	1.0380	1.0566	1.7295	1.1453	1.2522	3.2032	1.1111
1.0332	1.3216	1.1034	1.0221	1.0417	1.7703	1.1291	1.2574	3.1540	1.0708
1.0272	1.4010	1.1456	1.0274	1.0690	1.8828	1.1915	1.2303	3.2549	1.1004
1.0308	1.3663	1.1217	1.0252	1.0609	1.9101	1.1935	1.2305	3.6078	1.1159

PG16_118	PG16_118	PG18_018	PG18_018	PG18_020	PG18_118	PG18_118	PI16_018_	PI18_018_	PI18_020_
1.1337	1.1711	2.5148	1.0864	1.0298	3.3196	1.1165	2.1026	3.2601	1.1649
1.0837	1.0879	2.2877	1.0695	1.0121	2.4633	1.0808	1.8985	2.8625	1.1031
1.1098	1.1732	2.9083	1.0859	1.0341	4.3663	1.1386	1.5789	2.3778	1.0855
1.0871	1.1217	3.0403	1.0722	1.0275	4.5822	1.1106	1.4351	2.2407	1.0644
1.1250	1.1255	2.9125	1.1207	1.0359	3.1861	1.1311	1.5612	2.4872	1.1221
1.0984	1.1097	2.6987	1.0955	1.0275	3.0292	1.1073	1.7003	2.9292	1.1238
#NULL!	#NULL!	#NULL!	#NULL!	#NULL!	#NULL!	#NULL!	#NULL!	#NULL!	#NULL!
1.1045	1.1220	3.1681	1.1160	1.0407	4.1196	1.1511	1.4951	2.8420	1.1191
1.1347	1.1603	2.7883	1.0904	1.0301	3.6930	1.1316	1.5196	2.0679	1.0783
1.1030	1.1218	3.0528	1.0906	1.0489	3.9615	1.1224	1.4817	1.9848	1.1101
#NULL!	#NULL!	#NULL!	#NULL!	#NULL!	#NULL!	#NULL!	#NULL!	#NULL!	#NULL!
1.1093	1.1536	3.1580	1.1295	1.0365	4.5616	1.1967	1.3460	1.9638	1.0919
#NULL!	#NULL!	#NULL!	#NULL!	#NULL!	#NULL!	#NULL!	#NULL!	#NULL!	#NULL!
#NULL!	#NULL!	#NULL!	#NULL!	#NULL!	#NULL!	#NULL!	#NULL!	#NULL!	#NULL!
1.1201	1.1783	2.6284	1.1137	1.0298	4.0987	1.1840	1.5989	2.4249	1.1156
1.1092	1.1207	2.6922	1.0863	1.0234	3.1087	1.0997	1.5740	2.3201	1.0808
1.1373	1.1586	3.6556	1.1593	1.0614	4.3002	1.1786	1.3696	2.1480	1.1151
1.1516	1.2128	3.1080	1.1948	1.0542	4.1907	1.2696	1.3850	2.0721	1.1089
1.0869	1.1119	2.9376	1.1372	1.0318	4.0893	1.2069	1.5006	2.5111	1.1171
1.1000	1.1479	2.9965	1.1798	1.0309	4.2052	1.2739	1.4500	2.4303	1.1114
1.1112	1.1453	2.6686	1.1146	1.0412	3.6977	1.1724	1.6061	2.8647	1.1596
1.0589	1.0832	2.1383	1.0974	1.0290	3.1661	1.1875	1.4793	2.4258	1.1251
1.0950	1.1198	2.4041	1.0689	1.0197	2.9697	1.0975	1.5917	2.1049	1.0820
1.1236	1.1233	2.9559	1.1055	1.0345	3.3761	1.1153	1.7101	3.2198	1.0942
1.1474	1.1585	3.2670	1.1222	1.0486	3.8976	1.1506	1.4313	2.2565	1.0919
1.1208	1.1070	3.0627	1.0974	1.0364	3.1016	1.1002	1.6679	3.3243	1.1447
1.1023	1.1691	2.4367	1.1085	1.0187	3.7128	1.1920	1.5422	2.4221	1.1335
1.1212	1.1816	2.7921	1.2129	1.0241	4.5077	1.3721	1.3863	2.2436	1.1058
#NULL!	#NULL!	#NULL!	#NULL!	#NULL!	#NULL!	#NULL!	#NULL!	#NULL!	#NULL!
#NULL!	#NULL!	#NULL!	#NULL!	#NULL!	#NULL!	#NULL!	#NULL!	#NULL!	#NULL!
1.1572	1.1168	2.8923	1.0684	1.0261	2.7724	1.0597	1.5032	2.6993	1.0955
1.1111	1.1288	3.1520	1.1342	1.0389	3.9588	1.1723	1.4575	2.3221	1.0819
1.1193	1.1395	2.9582	1.1216	1.0385	3.7061	1.1686	1.5375	2.7332	1.1135
1.0846	1.1359	2.7602	1.1647	1.0330	4.3150	1.3009	1.3087	1.8382	1.0704
1.0820	1.0778	2.7904	1.1016	1.0166	3.0463	1.1102	1.2062	1.6464	1.0325
1.0665	1.0668	3.3411	1.1323	1.0514	3.7018	1.1426	1.4121	2.5129	1.1095
1.1115	1.1233	2.8334	1.0911	1.0251	3.2920	1.1135	1.5427	2.2185	1.0481
1.0821	1.1096	2.7429	1.0742	1.0235	3.7196	1.1101	1.4753	2.1355	1.0441
1.0577	1.0903	2.6126	1.1052	1.0307	3.5442	1.1469	1.6478	2.6360	1.1359
#NULL!	#NULL!	#NULL!	#NULL!	#NULL!	#NULL!	#NULL!	#NULL!	#NULL!	#NULL!
1.1278	1.1019	3.4675	1.1011	1.0496	3.2965	1.0905	1.3471	2.2197	1.0794
1.0958	1.1156	2.5362	1.0777	1.0253	2.9760	1.0906	1.4926	2.1654	1.1006
1.0867	1.1018	2.6773	1.0745	1.0286	3.2966	1.0911	1.3941	2.0838	1.0751
1.1257	1.1454	2.8860	1.1167	1.0480	3.2093	1.1327	1.4654	1.9696	1.0883
1.1297	1.1515	2.5100	1.1223	1.0382	2.8470	1.1557	1.5070	2.4719	1.0760
1.1485	1.1238	3.4871	1.1811	1.0488	3.3736	1.1682	1.2842	1.9677	1.0983

1.1165	1.1484	3.7278	1.2118	1.0482	4.9673	1.3164	1.2337	1.9171	1.1032
1.0618	1.0614	2.0944	1.0477	1.0157	2.4067	1.0463	2.0684	3.2002	1.1693
1.0672	1.0585	2.2922	1.0543	1.0144	2.3498	1.0563	1.8398	3.0515	1.1433
1.1686	1.1497	2.7654	1.0849	1.0502	3.0354	1.1026	1.6325	2.4696	1.1331
1.1160	1.0821	2.4722	1.0533	1.0196	2.2263	1.0498	1.4393	2.2254	1.0555
1.1229	1.1113	2.8282	1.0822	1.0372	3.0108	1.0886	1.4729	2.1590	1.0899
1.0685	1.0634	2.5810	1.0706	1.0231	2.7907	1.0794	1.4174	2.3438	1.0900
1.0930	1.1149	2.6795	1.0709	1.0272	3.4796	1.1070	1.5762	2.3335	1.0643
1.1338	1.1594	2.5230	1.0564	1.0233	3.1045	1.0728	1.5177	2.3081	1.0590
1.0673	1.0767	2.4299	1.1086	1.0191	2.9267	1.1368	1.5880	2.4941	1.0703
1.0450	1.0435	1.9287	1.0616	1.0168	2.1819	1.0761	1.8445	4.1771	1.1212
#NULL!	#NULL!	#NULL!	#NULL!	#NULL!	#NULL!	#NULL!	#NULL!	#NULL!	#NULL!
1.0584	1.0867	2.6612	1.1521	1.0374	3.2985	1.2176	1.2546	1.8642	1.0545
1.0628	1.0675	3.0997	1.1376	1.0353	3.6350	1.1709	1.2561	1.9376	1.0814
1.0858	1.0857	2.9562	1.0868	1.0351	3.6568	1.1166	1.2606	1.9761	1.0882
1.1798	1.1642	2.7432	1.1228	1.0373	2.8994	1.1229	1.5377	2.3267	1.1349
1.1598	1.1303	2.8163	1.1141	1.0357	2.6378	1.1079	1.3805	1.9651	1.1358
1.0541	1.0755	2.4593	1.0914	1.0240	3.1801	1.1265	1.4144	2.3630	1.1180
1.1132	1.1211	2.6610	1.0920	1.0323	2.9718	1.1117	1.4564	2.5297	1.1338
1.1089	1.0914	2.5253	1.0524	1.0246	2.5607	1.0559	1.5991	2.6590	1.1028
1.1276	1.1373	2.3242	1.0669	1.0253	2.7419	1.0815	1.7294	2.6373	1.1124
1.1282	1.1034	2.3945	1.0556	1.0175	2.3583	1.0494	2.0738	3.3387	1.1368

PI18_118_	SMd18_11	SMd18_11	SMd18_11	SMd18_11	SMd18_11	SMd18_11	SMd18_11	SMd18_11	HexCer
5.2964	1.0017	1.0237	1.0019	1.2442	1.0201	1.0043	1.0246	1.0063	########
3.8052	1.0021	1.0295	1.0016	1.2796	1.0210	1.0056	1.0252	1.0057	########
4.0070	1.0010	1.0205	1.0011	1.2997	1.0179	1.0069	1.0522	1.0061	########
3.3855	1.0007	1.0153	1.0012	1.1921	1.0138	1.0056	1.0240	1.0044	########
3.1170	1.0008	1.0148	1.0008	1.1265	1.0087	1.0030	1.0187	1.0040	########
3.6913	1.0008	1.0134	1.0011	1.1065	1.0078	1.0026	1.0144	1.0030	########
#NULL!	#NULL!	#NULL!	#NULL!	#NULL!	#NULL!	#NULL!	#NULL!	#NULL!	#NULL!
4.2342	1.0012	1.0128	1.0012	1.1231	1.0091	1.0022	1.0156	1.0035	########
3.0671	1.0008	1.0121	1.0013	1.0994	1.0090	1.0024	1.0129	1.0031	########
2.7944	1.0013	1.0214	1.0014	1.1853	1.0125	1.0106	1.0302	1.0045	########
#NULL!	#NULL!	#NULL!	#NULL!	#NULL!	#NULL!	#NULL!	#NULL!	#NULL!	#NULL!
2.8322	1.0016	1.0194	1.0013	1.1729	1.0124	1.0035	1.0176	1.0040	########
#NULL!	#NULL!	#NULL!	#NULL!	#NULL!	#NULL!	#NULL!	#NULL!	#NULL!	#NULL!
#NULL!	#NULL!	#NULL!	#NULL!	#NULL!	#NULL!	#NULL!	#NULL!	#NULL!	#NULL!
3.8703	1.0008	1.0197	1.0009	1.2009	1.0151	1.0029	1.0166	1.0037	########
2.9910	1.0040	1.0503	1.0023	1.5305	1.0227	1.0154	1.2211	1.0282	########
2.6814	1.0020	1.0214	1.0018	1.1495	1.0141	1.0036	1.0223	1.0052	########
2.9711	1.0013	1.0228	1.0006	1.2150	1.0173	1.0054	1.0227	1.0052	########
3.8203	1.0009	1.0140	1.0008	1.1426	1.0114	1.0030	1.0170	1.0040	########
3.8608	1.0012	1.0149	1.0010	1.1543	1.0118	1.0029	1.0182	1.0034	########
4.7220	1.0008	1.0234	1.0011	1.2936	1.0228	1.0048	1.0299	1.0070	########
4.0531	1.0033	1.1109	1.0028	3.5213	1.0628	1.0849	1.4941	1.0362	########
2.8811	1.0009	1.0145	1.0007	1.1698	1.0080	1.0023	1.0212	1.0033	########
4.3679	1.0008	1.0177	1.0011	1.2227	1.0185	1.0044	1.0251	1.0050	########
3.0943	1.0013	1.0144	1.0013	1.1574	1.0121	1.0031	1.0241	1.0052	########
3.6845	1.0009	1.0140	1.0010	1.1383	1.0091	1.0030	1.0179	1.0032	########
3.7620	1.0009	1.0173	1.0008	1.2249	1.0143	1.0043	1.0342	1.0048	########
3.3589	1.0009	1.0135	1.0011	1.1454	1.0104	1.0024	1.0235	1.0035	########
#NULL!	#NULL!	#NULL!	#NULL!	#NULL!	#NULL!	#NULL!	#NULL!	#NULL!	#NULL!
#NULL!	#NULL!	#NULL!	#NULL!	#NULL!	#NULL!	#NULL!	#NULL!	#NULL!	#NULL!
3.0554	1.0017	1.0182	1.0014	1.1604	1.0092	1.0030	1.0181	1.0031	########
3.3267	1.0011	1.0155	1.0013	1.1346	1.0115	1.0025	1.0171	1.0044	########
4.0735	1.0011	1.0148	1.0012	1.1488	1.0114	1.0027	1.0192	1.0045	########
2.6499	1.0010	1.0192	1.0008	1.1907	1.0137	1.0032	1.0201	1.0040	########
1.9057	1.0015	1.0204	1.0012	1.1839	1.0120	1.0040	1.0252	1.0038	########
3.0802	1.0022	1.0141	1.0012	1.1149	1.0087	1.0018	1.0194	1.0032	########
3.1980	1.0009	1.0128	1.0009	1.1401	1.0093	1.0025	1.0194	1.0038	########
3.3455	1.0008	1.0119	1.0011	1.1395	1.0094	1.0021	1.0190	1.0033	########
3.7775	1.0006	1.0128	1.0009	1.1947	1.0108	1.0032	1.0202	1.0037	########
#NULL!	#NULL!	#NULL!	#NULL!	#NULL!	#NULL!	#NULL!	#NULL!	#NULL!	#NULL!
2.3454	1.0007	1.0142	1.0008	1.1640	1.0104	1.0037	1.0226	1.0035	########
2.8966	1.0011	1.0203	1.0014	1.1750	1.0122	1.0029	1.0270	1.0048	########
2.7907	1.0010	1.0214	1.0011	1.2235	1.0133	1.0071	1.0310	1.0059	########
2.5345	1.0016	1.0191	1.0010	1.1936	1.0155	1.0034	1.0168	1.0039	########
3.2849	1.0012	1.0169	1.0008	1.1958	1.0144	1.0030	1.0172	1.0038	########
2.1491	1.0020	1.0223	1.0010	1.2199	1.0142	1.0041	1.0217	1.0041	########

2.5364	1.0012	1.0171	1.0013	1.1649	1.0142	1.0030	1.0174	1.0042	########
4.4701	1.0006	1.0161	1.0000	1.2387	1.0110	1.0049	1.0319	1.0050	########
3.3433	1.0014	1.0230	1.0010	1.2635	1.0131	1.0054	1.0271	1.0046	########
3.4141	1.0014	1.0237	1.0007	1.2837	1.0109	1.0081	1.0426	1.0050	########
2.2884	1.0015	1.0158	1.0009	1.1528	1.0069	1.0028	1.0249	1.0035	########
2.6830	1.0012	1.0135	1.0008	1.1241	1.0078	1.0033	1.0221	1.0036	########
2.8759	1.0039	1.0838	1.0037	1.8002	1.0594	1.0244	1.1397	1.0367	########
3.4026	1.0012	1.0150	1.0009	1.1345	1.0101	1.0031	1.0164	1.0033	########
3.3264	1.0010	1.0134	1.0008	1.1161	1.0083	1.0028	1.0167	1.0033	########
3.3700	1.0016	1.0141	1.0006	1.2231	1.0078	1.0056	1.0280	1.0031	########
5.4006	1.0020	1.0187	1.0007	1.2022	1.0082	1.0029	1.0163	1.0019	########
#NULL!	#NULL!	#NULL!	#NULL!	#NULL!	#NULL!	#NULL!	#NULL!	#NULL!	#NULL!
2.4714	1.0011	1.0175	1.0008	1.1408	1.0084	1.0028	1.0145	1.0028	########
2.3931	1.0006	1.0159	1.0007	1.1638	1.0085	1.0035	1.0183	1.0030	########
2.7663	1.0010	1.0149	1.0008	1.1629	1.0092	1.0034	1.0206	1.0035	########
2.7914	1.0014	1.0232	1.0010	1.2111	1.0119	1.0048	1.0265	1.0036	########
2.0561	1.0013	1.0187	1.0010	1.1445	1.0104	1.0025	1.0170	1.0035	########
3.2299	1.0008	1.0164	1.0008	1.1624	1.0086	1.0027	1.0211	1.0030	########
3.0994	1.0018	1.0193	1.0011	1.1676	1.0122	1.0031	1.0207	1.0033	########
3.2094	1.0011	1.0195	1.0008	1.2380	1.0122	1.0039	1.0219	1.0038	########
3.7796	1.0021	1.0280	1.0012	1.2826	1.0202	1.0037	1.0231	1.0052	########
3.9427	1.0011	1.0284	1.0009	1.3283	1.0138	1.0133	1.0454	1.0055	########

LPC	LPE	PC	PE	PG	PI	SM	CNTN6	TREM2	TF
########	########	########	########	########	########	########	########	########	########
########	########	########	########	########	########	########	########	########	########
########	########	########	########	########	########	########	########	########	########
########	########	########	########	########	########	########	########	########	########
########	########	########	########	########	########	########	#NULL!	#NULL!	#NULL!
########	########	########	########	########	########	########	########	########	########
#NULL!	#NULL!	#NULL!	#NULL!	#NULL!	#NULL!	#NULL!	########	########	########
########	########	########	########	########	########	########	########	########	########
########	########	########	########	########	########	########	#NULL!	#NULL!	#NULL!
########	########	########	########	########	########	########	########	########	########
#NULL!	#NULL!	#NULL!	#NULL!	#NULL!	#NULL!	#NULL!	########	########	########
########	########	########	########	########	########	########	#NULL!	#NULL!	#NULL!
#NULL!	#NULL!	#NULL!	#NULL!	#NULL!	#NULL!	#NULL!	#NULL!	#NULL!	#NULL!
#NULL!	#NULL!	#NULL!	#NULL!	#NULL!	#NULL!	#NULL!	########	########	########
########	########	########	########	########	########	########	########	########	########
########	########	########	########	########	########	########	########	########	########
########	########	########	########	########	########	########	########	########	########
########	########	########	########	########	########	########	########	########	########
########	########	########	########	########	########	########	#NULL!	#NULL!	#NULL!
########	########	########	########	########	########	########	#NULL!	#NULL!	#NULL!
########	########	########	########	########	########	########	#NULL!	#NULL!	#NULL!
########	########	########	########	########	########	########	########	########	########
########	########	########	########	########	########	########	########	########	########
########	########	########	########	########	########	########	########	########	########
########	########	########	########	########	########	########	########	########	########
########	########	########	########	########	########	########	#NULL!	#NULL!	#NULL!
########	########	########	########	########	########	########	########	########	########
########	########	########	########	########	########	########	########	########	########
#NULL!	#NULL!	#NULL!	#NULL!	#NULL!	#NULL!	#NULL!	#NULL!	#NULL!	#NULL!
#NULL!	#NULL!	#NULL!	#NULL!	#NULL!	#NULL!	#NULL!	#NULL!	#NULL!	#NULL!
########	########	########	########	########	########	########	########	########	########
########	########	########	########	########	########	########	########	########	########
########	########	########	########	########	########	########	########	########	########
########	########	########	########	########	########	########	########	########	########
########	########	########	########	########	########	########	#NULL!	#NULL!	#NULL!
########	########	########	########	########	########	########	########	########	########
########	########	########	########	########	########	########	#NULL!	#NULL!	#NULL!
########	########	########	########	########	########	########	########	########	########
########	########	########	########	########	########	########	#NULL!	#NULL!	#NULL!
#NULL!	#NULL!	#NULL!	#NULL!	#NULL!	#NULL!	#NULL!	#NULL!	#NULL!	#NULL!
########	########	########	########	########	########	########	########	########	########
########	########	########	########	########	########	########	########	########	########
########	########	########	########	########	########	########	########	########	########
########	########	########	########	########	########	########	########	########	########
########	########	########	########	########	########	########	########	########	########
########	########	########	########	########	########	########	########	########	########

########	########	########	########	########	########	########	#NULL!	#NULL!	#NULL!
########	########	########	########	########	########	########	########	########	########
########	########	########	########	########	########	########	########	########	########
########	########	########	########	########	########	########	########	########	########
########	########	########	########	########	########	########	########	########	########
########	########	########	########	########	########	########	########	########	########
########	########	########	########	########	########	########	#NULL!	#NULL!	#NULL!
########	########	########	########	########	########	########	########	########	########
########	########	########	########	########	########	########	#NULL!	#NULL!	#NULL!
########	########	########	########	########	########	########	########	########	########
########	########	########	########	########	########	########	#NULL!	#NULL!	#NULL!
#NULL!	#NULL!	#NULL!	#NULL!	#NULL!	#NULL!	#NULL!	########	########	########
########	########	########	########	########	########	########	########	########	########
########	########	########	########	########	########	########	########	########	########
########	########	########	########	########	########	########	########	########	########
########	########	########	########	########	########	########	########	########	########
########	########	########	########	########	########	########	########	########	########
########	########	########	########	########	########	########	########	########	########
########	########	########	########	########	########	########	########	########	########
########	########	########	########	########	########	########	########	########	########
########	########	########	########	########	########	########	#NULL!	#NULL!	#NULL!
########	########	########	########	########	########	########	########	########	########

F2	FCGR3B	HPX	SERPINC1	CCL18	CRTAC1	SFTPD	SERPING1	PLXND1	CFI
########	########	########	########	########	########	########	########	209.5	########
########	########	########	########	########	########	########	########	177.6	########
########	########	########	########	########	########	########	########	193.8	########
########	########	########	########	########	########	########	########	195.7	########
#NULL!	#NULL!	#NULL!	#NULL!	#NULL!	#NULL!	#NULL!	#NULL!	#NULL!	#NULL!
########	########	########	########	########	########	########	########	257.2	########
########	########	########	########	########	########	########	########	218.4	########
########	########	########	########	########	########	########	########	227.3	########
#NULL!	#NULL!	#NULL!	#NULL!	#NULL!	#NULL!	#NULL!	#NULL!	#NULL!	#NULL!
########	########	########	########	########	########	########	########	200.0	########
########	########	########	########	########	########	########	########	213.3	########
#NULL!	#NULL!	#NULL!	#NULL!	#NULL!	#NULL!	#NULL!	#NULL!	#NULL!	#NULL!
#NULL!	#NULL!	#NULL!	#NULL!	#NULL!	#NULL!	#NULL!	#NULL!	#NULL!	#NULL!
########	########	########	########	########	########	########	########	215.8	########
########	########	########	########	########	########	########	########	197.0	########
########	########	########	########	########	########	########	########	191.6	########
########	########	########	########	########	########	########	########	218.8	########
########	########	########	########	########	########	########	########	213.8	########
#NULL!	#NULL!	#NULL!	#NULL!	#NULL!	#NULL!	#NULL!	#NULL!	#NULL!	#NULL!
#NULL!	#NULL!	#NULL!	#NULL!	#NULL!	#NULL!	#NULL!	#NULL!	#NULL!	#NULL!
#NULL!	#NULL!	#NULL!	#NULL!	#NULL!	#NULL!	#NULL!	#NULL!	#NULL!	#NULL!
########	########	########	########	########	########	########	########	200.8	########
########	########	########	########	########	########	########	########	226.5	########
########	########	########	########	########	########	########	########	189.7	########
########	########	########	########	########	########	########	########	202.0	########
#NULL!	#NULL!	#NULL!	#NULL!	#NULL!	#NULL!	#NULL!	#NULL!	#NULL!	#NULL!
########	########	########	########	########	########	########	########	201.9	########
########	########	########	########	########	########	########	########	218.9	########
#NULL!	#NULL!	#NULL!	#NULL!	#NULL!	#NULL!	#NULL!	#NULL!	#NULL!	#NULL!
#NULL!	#NULL!	#NULL!	#NULL!	#NULL!	#NULL!	#NULL!	#NULL!	#NULL!	#NULL!
########	########	########	########	########	########	########	########	223.2	########
########	########	########	########	########	########	########	########	197.1	########
########	########	########	########	########	########	########	########	204.3	########
########	########	########	########	########	########	########	########	202.3	########
#NULL!	#NULL!	#NULL!	#NULL!	#NULL!	#NULL!	#NULL!	#NULL!	#NULL!	#NULL!
########	########	########	########	########	########	########	########	210.6	########
#NULL!	#NULL!	#NULL!	#NULL!	#NULL!	#NULL!	#NULL!	#NULL!	#NULL!	#NULL!
########	########	########	########	########	########	########	########	197.9	########
#NULL!	#NULL!	#NULL!	#NULL!	#NULL!	#NULL!	#NULL!	#NULL!	#NULL!	#NULL!
#NULL!	#NULL!	#NULL!	#NULL!	#NULL!	#NULL!	#NULL!	#NULL!	#NULL!	#NULL!
########	########	########	########	########	########	########	########	205.5	########
########	########	########	########	########	########	########	########	235.2	########
########	########	########	########	########	########	########	########	220.2	########
########	########	########	########	########	########	########	########	209.7	########
########	########	########	########	########	########	########	########	203.9	########
########	########	########	########	########	########	########	########	199.2	########

#NULL!	#NULL!	#NULL!	#NULL!	#NULL!	#NULL!	#NULL!	#NULL!	#NULL!	#NULL!
########	########	########	########	########	########	########	########	183.9	########
########	########	########	########	########	########	########	########	199.7	########
########	########	########	########	########	########	########	########	225.8	########
########	########	########	########	########	########	########	########	228.3	########
########	########	########	########	########	########	########	########	190.7	########
#NULL!	#NULL!	#NULL!	#NULL!	#NULL!	#NULL!	#NULL!	#NULL!	#NULL!	#NULL!
########	########	########	########	########	########	########	########	209.4	########
#NULL!	#NULL!	#NULL!	#NULL!	#NULL!	#NULL!	#NULL!	#NULL!	#NULL!	#NULL!
########	########	########	########	########	########	########	########	208.6	########
#NULL!	#NULL!	#NULL!	#NULL!	#NULL!	#NULL!	#NULL!	#NULL!	#NULL!	#NULL!
########	########	########	########	########	########	########	########	212.6	########
########	########	########	########	########	########	########	########	207.0	########
########	########	########	########	########	########	########	########	198.0	########
########	########	########	########	########	########	########	########	195.4	########
########	########	########	########	########	########	########	########	207.8	########
########	########	########	########	########	########	########	########	206.1	########
########	########	########	########	########	########	########	########	202.7	########
########	########	########	########	########	########	########	########	189.2	########
########	########	########	########	########	########	########	########	207.8	########
#NULL!	#NULL!	#NULL!	#NULL!	#NULL!	#NULL!	#NULL!	#NULL!	#NULL!	#NULL!
########	########	########	########	########	########	########	########	221.7	########

BTD	LUM	VSIG4	PTPRS	FABP3	ALB	ITIH2	GSN	HPX_A	HPX_B
########	########	########	########	########	25622.0	########	########	########	########
########	########	########	########	########	19349.0	########	########	########	########
########	########	########	########	########	24153.0	########	########	########	########
########	########	########	########	########	18807.0	########	########	########	########
#NULL!	#NULL!	#NULL!	#NULL!	#NULL!	#NULL!	#NULL!	#NULL!	#NULL!	#NULL!
########	########	########	########	########	11332.0	########	########	########	########
########	########	########	########	########	12880.0	########	########	########	########
########	########	########	########	########	11784.0	########	########	########	########
#NULL!	#NULL!	#NULL!	#NULL!	#NULL!	#NULL!	#NULL!	#NULL!	#NULL!	#NULL!
########	########	########	########	########	17222.0	########	########	########	########
########	########	########	########	########	19706.0	########	########	########	########
#NULL!	#NULL!	#NULL!	#NULL!	#NULL!	#NULL!	#NULL!	#NULL!	#NULL!	#NULL!
#NULL!	#NULL!	#NULL!	#NULL!	#NULL!	#NULL!	#NULL!	#NULL!	#NULL!	#NULL!
########	########	########	########	########	21387.0	########	########	########	########
########	########	########	########	########	12928.0	########	########	########	########
########	########	########	########	########	12095.0	########	########	########	########
########	########	########	########	########	22425.0	########	########	########	########
########	########	########	########	########	16404.0	########	########	########	########
#NULL!	#NULL!	#NULL!	#NULL!	#NULL!	#NULL!	#NULL!	#NULL!	#NULL!	#NULL!
#NULL!	#NULL!	#NULL!	#NULL!	#NULL!	#NULL!	#NULL!	#NULL!	#NULL!	#NULL!
#NULL!	#NULL!	#NULL!	#NULL!	#NULL!	#NULL!	#NULL!	#NULL!	#NULL!	#NULL!
########	########	########	########	########	21227.0	########	########	########	########
########	########	########	########	########	21203.0	########	########	########	########
########	########	########	########	########	20331.0	########	########	########	########
########	########	########	########	########	14660.0	########	########	########	########
#NULL!	#NULL!	#NULL!	#NULL!	#NULL!	#NULL!	#NULL!	#NULL!	#NULL!	#NULL!
########	########	########	########	########	15781.0	########	########	########	########
########	########	########	########	########	13953.0	########	########	########	########
#NULL!	#NULL!	#NULL!	#NULL!	#NULL!	#NULL!	#NULL!	#NULL!	#NULL!	#NULL!
#NULL!	#NULL!	#NULL!	#NULL!	#NULL!	#NULL!	#NULL!	#NULL!	#NULL!	#NULL!
########	########	########	########	########	13878.0	########	########	########	########
########	########	########	########	########	11857.0	########	########	########	########
########	########	########	########	########	27896.0	########	########	########	########
########	########	########	########	########	16534.0	########	########	########	########
#NULL!	#NULL!	#NULL!	#NULL!	#NULL!	#NULL!	#NULL!	#NULL!	#NULL!	#NULL!
########	########	########	########	########	11431.0	########	########	########	########
#NULL!	#NULL!	#NULL!	#NULL!	#NULL!	#NULL!	#NULL!	#NULL!	#NULL!	#NULL!
########	########	########	########	########	13755.0	########	########	########	########
#NULL!	#NULL!	#NULL!	#NULL!	#NULL!	#NULL!	#NULL!	#NULL!	#NULL!	#NULL!
#NULL!	#NULL!	#NULL!	#NULL!	#NULL!	#NULL!	#NULL!	#NULL!	#NULL!	#NULL!
########	########	########	########	########	17082.0	########	########	########	########
########	########	########	########	########	10795.0	########	########	########	########
########	########	########	########	########	24010.0	########	########	########	########
########	########	########	########	########	25379.0	########	########	########	########
########	########	########	########	########	22585.0	########	########	########	########
########	########	########	########	########	13723.0	########	########	########	########

#NULL!	#NULL!	#NULL!	#NULL!	#NULL!	#NULL!	#NULL!	#NULL!	#NULL!	#NULL!
########	########	########	########	########	7645.5	########	########	########	########
########	########	########	########	########	17345.0	########	########	########	########
########	########	########	########	########	7489.3	########	########	########	########
########	########	########	########	########	14161.0	########	########	########	########
########	########	########	########	########	12292.0	########	########	########	########
#NULL!	#NULL!	#NULL!	#NULL!	#NULL!	#NULL!	#NULL!	#NULL!	#NULL!	#NULL!
########	########	########	########	########	8859.5	########	########	########	########
#NULL!	#NULL!	#NULL!	#NULL!	#NULL!	#NULL!	#NULL!	#NULL!	#NULL!	#NULL!
########	########	########	########	########	21888.0	########	########	########	########
#NULL!	#NULL!	#NULL!	#NULL!	#NULL!	#NULL!	#NULL!	#NULL!	#NULL!	#NULL!
########	########	########	########	########	20630.0	########	########	########	########
########	########	########	########	########	10754.0	########	########	########	########
########	########	########	########	########	16628.0	########	########	########	########
########	########	########	########	########	15276.0	########	########	########	########
########	########	########	########	########	17053.0	########	########	########	########
########	########	########	########	########	23303.0	########	########	########	########
########	########	########	########	########	18910.0	########	########	########	########
########	########	########	########	########	19955.0	########	########	########	########
########	########	########	########	########	20682.0	########	########	########	########
#NULL!	#NULL!	#NULL!	#NULL!	#NULL!	#NULL!	#NULL!	#NULL!	#NULL!	#NULL!
########	########	########	########	########	28824.0	########	########	########	########

C4AC4B	MRC1	C3	EFNA1	GC	AGT	SERPINA7	B4GALT1	CD55	EDIL3
########	########	########	########	########	########	########	########	########	########
########	########	########	########	########	########	########	########	########	########
########	########	########	########	########	########	########	########	########	########
########	########	########	########	########	########	########	########	########	########
#NULL!	#NULL!	#NULL!	#NULL!	#NULL!	#NULL!	#NULL!	#NULL!	#NULL!	#NULL!
########	########	########	########	########	########	########	########	########	########
########	########	########	########	########	########	########	########	########	########
########	########	########	########	########	########	########	########	########	########
#NULL!	#NULL!	#NULL!	#NULL!	#NULL!	#NULL!	#NULL!	#NULL!	#NULL!	#NULL!
########	########	########	########	########	########	########	########	########	########
########	########	########	########	########	########	########	########	########	########
#NULL!	#NULL!	#NULL!	#NULL!	#NULL!	#NULL!	#NULL!	#NULL!	#NULL!	#NULL!
#NULL!	#NULL!	#NULL!	#NULL!	#NULL!	#NULL!	#NULL!	#NULL!	#NULL!	#NULL!
########	########	########	########	########	########	########	########	########	########
########	########	########	########	########	########	########	########	########	########
########	########	########	########	########	########	########	########	########	########
########	########	########	########	########	########	########	########	########	########
########	########	########	########	########	########	########	########	########	########
#NULL!	#NULL!	#NULL!	#NULL!	#NULL!	#NULL!	#NULL!	#NULL!	#NULL!	#NULL!
#NULL!	#NULL!	#NULL!	#NULL!	#NULL!	#NULL!	#NULL!	#NULL!	#NULL!	#NULL!
#NULL!	#NULL!	#NULL!	#NULL!	#NULL!	#NULL!	#NULL!	#NULL!	#NULL!	#NULL!
########	########	########	########	########	########	########	########	########	########
########	########	########	########	########	########	########	########	########	########
########	########	########	########	########	########	########	########	########	########
########	########	########	########	########	########	########	########	########	########
#NULL!	#NULL!	#NULL!	#NULL!	#NULL!	#NULL!	#NULL!	#NULL!	#NULL!	#NULL!
########	########	########	########	########	########	########	########	########	########
########	########	########	########	########	########	########	########	########	########
#NULL!	#NULL!	#NULL!	#NULL!	#NULL!	#NULL!	#NULL!	#NULL!	#NULL!	#NULL!
#NULL!	#NULL!	#NULL!	#NULL!	#NULL!	#NULL!	#NULL!	#NULL!	#NULL!	#NULL!
########	########	########	########	########	########	########	########	########	########
########	########	########	########	########	########	########	########	########	########
########	########	########	########	########	########	########	########	########	########
########	########	########	########	########	########	########	########	########	########
#NULL!	#NULL!	#NULL!	#NULL!	#NULL!	#NULL!	#NULL!	#NULL!	#NULL!	#NULL!
########	########	########	########	########	########	########	########	########	########
#NULL!	#NULL!	#NULL!	#NULL!	#NULL!	#NULL!	#NULL!	#NULL!	#NULL!	#NULL!
########	########	########	########	########	########	########	########	########	########
#NULL!	#NULL!	#NULL!	#NULL!	#NULL!	#NULL!	#NULL!	#NULL!	#NULL!	#NULL!
#NULL!	#NULL!	#NULL!	#NULL!	#NULL!	#NULL!	#NULL!	#NULL!	#NULL!	#NULL!
########	########	########	########	########	########	########	########	########	########
########	########	########	########	########	########	########	########	########	########
########	########	########	########	########	########	########	########	########	########
########	########	########	########	########	########	########	########	########	########
########	########	########	########	########	########	########	########	########	########
########	########	########	########	########	########	########	########	########	########

#NULL!	#NULL!	#NULL!	#NULL!	#NULL!	#NULL!	#NULL!	#NULL!	#NULL!	#NULL!
########	########	########	########	########	########	########	########	########	########
########	########	########	########	########	########	########	########	########	########
########	########	########	########	########	########	########	########	########	########
########	########	########	########	########	########	########	########	########	########
########	########	########	########	########	########	########	########	########	########
#NULL!	#NULL!	#NULL!	#NULL!	#NULL!	#NULL!	#NULL!	#NULL!	#NULL!	#NULL!
########	########	########	########	########	########	########	########	########	########
#NULL!	#NULL!	#NULL!	#NULL!	#NULL!	#NULL!	#NULL!	#NULL!	#NULL!	#NULL!
########	########	########	########	########	########	########	########	########	########
#NULL!	#NULL!	#NULL!	#NULL!	#NULL!	#NULL!	#NULL!	#NULL!	#NULL!	#NULL!
########	########	########	########	########	########	########	########	########	########
########	########	########	########	########	########	########	########	########	########
########	########	########	########	########	########	########	########	########	########
########	########	########	########	########	########	########	########	########	########
########	########	########	########	########	########	########	########	########	########
########	########	########	########	########	########	########	########	########	########
########	########	########	########	########	########	########	########	########	########
########	########	########	########	########	########	########	########	########	########
########	########	########	########	########	########	########	########	########	########
#NULL!	#NULL!	#NULL!	#NULL!	#NULL!	#NULL!	#NULL!	#NULL!	#NULL!	#NULL!
########	########	########	########	########	########	########	########	########	########

RBP4	NOG	AHSG	C5	CFB	APOH	SOD3	IL6ST	HP	CFD
########	########	181.6	########	########	########	########	########	########	187.9
########	########	182.2	########	########	########	########	########	########	177.9
########	########	175.4	########	########	########	########	########	########	192.3
########	########	188.6	########	########	########	########	########	########	319.5
#NULL!	#NULL!	#NULL!	#NULL!	#NULL!	#NULL!	#NULL!	#NULL!	#NULL!	#NULL!
########	########	227.0	########	########	########	########	########	########	207.6
########	########	182.1	########	########	########	########	########	########	179.1
########	########	179.8	########	########	########	########	########	########	185.8
#NULL!	#NULL!	#NULL!	#NULL!	#NULL!	#NULL!	#NULL!	#NULL!	#NULL!	#NULL!
########	########	180.7	########	########	########	########	########	########	188.8
########	########	187.7	########	########	########	########	########	########	186.0
#NULL!	#NULL!	#NULL!	#NULL!	#NULL!	#NULL!	#NULL!	#NULL!	#NULL!	#NULL!
#NULL!	#NULL!	#NULL!	#NULL!	#NULL!	#NULL!	#NULL!	#NULL!	#NULL!	#NULL!
########	########	196.1	########	########	########	########	########	########	194.2
########	########	197.8	########	########	########	########	########	########	187.7
########	########	192.3	########	########	########	########	########	########	185.3
########	########	207.3	########	########	########	########	########	########	215.5
########	########	197.2	########	########	########	########	########	########	196.4
#NULL!	#NULL!	#NULL!	#NULL!	#NULL!	#NULL!	#NULL!	#NULL!	#NULL!	#NULL!
#NULL!	#NULL!	#NULL!	#NULL!	#NULL!	#NULL!	#NULL!	#NULL!	#NULL!	#NULL!
#NULL!	#NULL!	#NULL!	#NULL!	#NULL!	#NULL!	#NULL!	#NULL!	#NULL!	#NULL!
########	########	196.1	########	########	########	########	########	########	190.7
########	########	202.4	########	########	########	########	########	########	194.9
########	########	201.5	########	########	########	########	########	########	197.2
########	########	195.2	########	########	########	########	########	########	193.9
#NULL!	#NULL!	#NULL!	#NULL!	#NULL!	#NULL!	#NULL!	#NULL!	#NULL!	#NULL!
########	########	170.9	########	########	########	########	########	########	173.1
########	########	173.2	########	########	########	########	########	########	190.2
#NULL!	#NULL!	#NULL!	#NULL!	#NULL!	#NULL!	#NULL!	#NULL!	#NULL!	#NULL!
#NULL!	#NULL!	#NULL!	#NULL!	#NULL!	#NULL!	#NULL!	#NULL!	#NULL!	#NULL!
########	########	180.5	########	########	########	########	########	########	188.9
########	########	190.5	########	########	########	########	########	########	196.7
########	########	207.2	########	########	########	########	########	########	212.1
########	########	193.3	########	########	########	########	########	########	189.2
#NULL!	#NULL!	#NULL!	#NULL!	#NULL!	#NULL!	#NULL!	#NULL!	#NULL!	#NULL!
########	########	189.6	########	########	########	########	########	########	182.2
#NULL!	#NULL!	#NULL!	#NULL!	#NULL!	#NULL!	#NULL!	#NULL!	#NULL!	#NULL!
########	########	202.0	########	########	########	########	########	########	193.7
#NULL!	#NULL!	#NULL!	#NULL!	#NULL!	#NULL!	#NULL!	#NULL!	#NULL!	#NULL!
#NULL!	#NULL!	#NULL!	#NULL!	#NULL!	#NULL!	#NULL!	#NULL!	#NULL!	#NULL!
########	########	212.5	########	########	########	########	########	########	216.4
########	########	216.7	########	########	########	########	########	########	200.5
########	########	207.3	########	########	########	########	########	########	203.6
########	########	182.2	########	########	########	########	########	########	193.4
########	########	175.1	########	########	########	########	########	########	201.1
########	########	181.4	########	########	########	########	########	########	196.0

#NULL!	#NULL!	#NULL!	#NULL!	#NULL!	#NULL!	#NULL!	#NULL!	#NULL!	#NULL!
########	########	189.0	########	########	########	########	########	########	180.3
########	########	194.7	########	########	########	########	########	########	190.9
########	########	236.7	########	########	########	########	########	########	202.0
########	########	234.4	########	########	########	########	########	########	194.9
########	########	179.1	########	########	########	########	########	########	181.4
#NULL!	#NULL!	#NULL!	#NULL!	#NULL!	#NULL!	#NULL!	#NULL!	#NULL!	#NULL!
########	########	177.2	########	########	########	########	########	########	184.0
#NULL!	#NULL!	#NULL!	#NULL!	#NULL!	#NULL!	#NULL!	#NULL!	#NULL!	#NULL!
########	########	190.3	########	########	########	########	########	########	187.0
#NULL!	#NULL!	#NULL!	#NULL!	#NULL!	#NULL!	#NULL!	#NULL!	#NULL!	#NULL!
########	########	234.7	########	########	########	########	########	########	197.1
########	########	176.0	########	########	########	########	########	########	179.7
########	########	196.1	########	########	########	########	########	########	184.3
########	########	194.5	########	########	########	########	########	########	186.5
########	########	192.9	########	########	########	########	########	########	196.2
########	########	199.1	########	########	########	########	########	########	185.6
########	########	209.4	########	########	########	########	########	########	193.9
########	########	214.5	########	########	########	########	########	########	197.0
########	########	202.5	########	########	########	########	########	########	186.3
#NULL!	#NULL!	#NULL!	#NULL!	#NULL!	#NULL!	#NULL!	#NULL!	#NULL!	#NULL!
########	########	179.9	########	########	########	########	########	########	206.7

CEMIP2	CSF1R	NTM	NTM_A	NTM_B	FBP1	NLGN1
########	225.3	########	########	########	########	########
########	197.4	########	########	########	########	########
########	219.5	########	########	########	########	########
########	252.1	########	########	########	########	########
#NULL!	#NULL!	#NULL!	#NULL!	#NULL!	#NULL!	#NULL!
########	203.4	########	########	########	########	########
########	209.1	########	########	########	########	########
########	192.1	########	########	########	########	########
#NULL!	#NULL!	#NULL!	#NULL!	#NULL!	#NULL!	#NULL!
########	211.9	########	########	########	########	########
########	223.3	########	########	########	########	########
#NULL!	#NULL!	#NULL!	#NULL!	#NULL!	#NULL!	#NULL!
#NULL!	#NULL!	#NULL!	#NULL!	#NULL!	#NULL!	#NULL!
########	227.5	########	########	########	########	########
########	221.2	########	########	########	########	########
########	207.9	########	########	########	########	########
########	210.4	########	########	########	########	########
########	239.6	########	########	########	########	########
#NULL!	#NULL!	#NULL!	#NULL!	#NULL!	#NULL!	#NULL!
#NULL!	#NULL!	#NULL!	#NULL!	#NULL!	#NULL!	#NULL!
#NULL!	#NULL!	#NULL!	#NULL!	#NULL!	#NULL!	#NULL!
########	227.1	########	########	########	########	########
########	214.4	########	########	########	########	########
########	223.1	########	########	########	########	########
########	213.0	########	########	########	########	########
#NULL!	#NULL!	#NULL!	#NULL!	#NULL!	#NULL!	#NULL!
########	220.1	########	########	########	########	########
########	221.0	########	########	########	########	########
#NULL!	#NULL!	#NULL!	#NULL!	#NULL!	#NULL!	#NULL!
#NULL!	#NULL!	#NULL!	#NULL!	#NULL!	#NULL!	#NULL!
########	219.1	########	########	########	########	########
########	205.3	########	########	########	########	########
########	211.8	########	########	########	########	########
########	223.2	########	########	########	########	########
#NULL!	#NULL!	#NULL!	#NULL!	#NULL!	#NULL!	#NULL!
########	203.2	########	########	########	########	########
#NULL!	#NULL!	#NULL!	#NULL!	#NULL!	#NULL!	#NULL!
########	207.7	########	########	########	########	########
#NULL!	#NULL!	#NULL!	#NULL!	#NULL!	#NULL!	#NULL!
#NULL!	#NULL!	#NULL!	#NULL!	#NULL!	#NULL!	#NULL!
########	203.6	########	########	########	########	########
########	204.2	########	########	########	########	########
########	217.1	########	########	########	########	########
########	227.5	########	########	########	########	########
########	214.2	########	########	########	########	########
########	214.9	########	########	########	########	########

#NULL!	#NULL!	#NULL!	#NULL!	#NULL!	#NULL!	#NULL!
########	203.7	########	########	########	########	########
########	218.6	########	########	########	########	########
########	242.0	########	########	########	########	########
########	213.1	########	########	########	########	########
########	210.3	########	########	########	########	########
#NULL!	#NULL!	#NULL!	#NULL!	#NULL!	#NULL!	#NULL!
########	201.0	########	########	########	########	########
#NULL!	#NULL!	#NULL!	#NULL!	#NULL!	#NULL!	#NULL!
########	219.8	########	########	########	########	########
#NULL!	#NULL!	#NULL!	#NULL!	#NULL!	#NULL!	#NULL!
########	213.0	########	########	########	########	########
########	197.5	########	########	########	########	########
########	213.4	########	########	########	########	########
########	208.0	########	########	########	########	########
########	225.5	########	########	########	########	########
########	217.7	########	########	########	########	########
########	205.8	########	########	########	########	########
########	208.3	########	########	########	########	########
########	219.1	########	########	########	########	########
#NULL!	#NULL!	#NULL!	#NULL!	#NULL!	#NULL!	#NULL!
########	221.4	########	########	########	########	########
